# Specialized Metabolism of *Gordonia* Genus: An Integrated Survey on Chemodiversity Combined with a Comparative Genomics-Based Analysis

**DOI:** 10.3390/biotech11040053

**Published:** 2022-11-21

**Authors:** Jeysson Sánchez-Suárez, Luis Díaz, Ericsson Coy-Barrera, Luisa Villamil

**Affiliations:** 1 Doctoral Program in Biosciences, School of Engineering, Universidad de La Sabana, Chía 250001, Colombia; 2 Bioprospecting Research Group, School of Engineering, Universidad de La Sabana, Chía 250001, Colombia; 3 Bioorganic Chemistry Laboratory, Universidad Militar Nueva Granada, Cajicá 250247, Colombia

**Keywords:** *Actinobacteria*, *Mycobacteriales*, secondary metabolites, naturally occurring compounds, biosynthetic gene clusters

## Abstract

Members of the phylum *Actinomycetota* (formerly *Actinobacteria*) have historically been the most prolific providers of small bioactive molecules. Although the genus *Streptomyces* is the best-known member for this issue, other genera, such as *Gordonia*, have shown interesting potential in their specialized metabolism. Thus, we combined herein the result of a comprehensive literature survey on metabolites derived from *Gordonia* strains with a comparative genomic analysis to examine the potential of the specialized metabolism of the genus *Gordonia*. Thirty *Gordonia*-derived compounds of different classes were gathered (i.e., alkaloids, amides, phenylpropanoids, and terpenoids), exhibiting antimicrobial and cytotoxic activities, and several were also isolated from *Streptomyces* (e.g., actinomycin, nocardamin, diolmycin A1). With the genome data, we estimated an open pan-genome of 57,901 genes, most of them being part of the cloud genome. Regarding the BGCs content, 531 clusters were found, including Terpenes, RiPP-like, and NRPS clusters as the most frequent clusters. Our findings demonstrated that *Gordonia* is a poorly studied genus in terms of its specialized metabolism production and potential applications. Nevertheless, given their BGCs content, *Gordonia* spp. are a valuable biological resource that could expand the chemical spectrum of the phylum *Actinomycetota*, involving novel BGCs for inspiring innovative outlines for synthetic biology and further use in biotechnological initiatives. Therefore, further studies and more efforts should be made to explore different environments and evaluate other bioactivities.

## 1. Introduction

The phylum *Actinomycetota* is a priceless bioresource of active metabolites due to its diversified specialized metabolism [1]. Among over 250 genera described within the phylum, *Streptomyces* is the most prominent [1], especially for providing the most clinically used antibiotics [2]. Moreover, some studies have reported that species of this genus produce bioactivities other than antimicrobial, such as anticancer, immunomodulatory, and antioxidant [2,3]. Consequently, the research on streptomycetes’ bioprospecting has continued to attract interest. Although non-streptomyces actinomycetes (also known as rare actinomycetes) have been relegated [4], evidence has recently been growing for their potential to provide novel bioactive compounds [5]. Some rare actinomycetes genera showing a high chemodiversity in their specialized metabolism are *Corynebacterium*, *Nocardiopsis*, *Saccharomonospora*, *Pseudonocardia,* and *Gordonia* [6].

*Gordonia* genus was established in 1988 [7] and is characterized by mycelial growth with fragmentation into rod-shaped elements or cocci, pigmented colonies (e.g., yellow, orange, red), and a G + C content between 63 and 69% [8]. Although some strains are opportunistic human pathogens (e.g., *Gordonia sputi*, *Gordonia bronchialis*, and *Gordonia terrae* [9]), several species have been reported to play crucial ecological niches, notably as symbionts [10]. This aspect is relevant in the natural products field, considering the importance of symbiotic bacteria in the chemical ecology of their hosts [11].

Regarding the bioprospecting potential of *Gordonia* species, a large amount of literature on bioremediation has involved *Gordonia* strains [10]. However, their specialized metabolites’ isolation and bioactivity profiling remain poorly understood [12]. This fact constitutes a promising field for research, mainly due to the possibility of finding unique chemical structures or the opportunity to discover more sustainable sources of bioactive natural products (i.e., through microbial biotechnology) [13,14,15].

Natural products have been the primary drug source [16]. Recently, the role of microorganisms as producers of high-value-added products (e.g., bioactive compounds) has gained much attention [17]. Among the various reasons, it is worth emphasizing its contribution to the bioeconomy and its engagement with the Sustainable Development Goals (SDGs) [18,19]. Considering that pharmaceuticals are at the top of the bioeconomy value pyramid [20], microbial biotechnology is a crucial component in the development of a bio-based economy [21] and, therefore, to achieve sustainable economic development (i.e., SDG8) [19,22], while contributing to the good health and well-being (i.e., SDG3) [19,23]. In this context, exploring the available biological resources and their hidden potential as thoroughly as possible becomes a high-priority research topic.

As a result of new advances in genome mining tools, such as antiSMASH [24] and BiG-Scape [25], the massification of whole-genome sequencing projects and the resulting large volume of data has opened the possibility of innovative alternatives for natural product research [26]. The enzymes involved in specialized metabolite biosynthesis pathways are encoded in genes arranged in clusters, i.e., biosynthetic gene clusters (BGCs) [27]. The diversity and abundance of these BGCs (namely BGC space) are linked to a certain taxon-depending chemical space [1,28]. Therefore, mapping the arsenal of BGCs associated with an organism or group lets rational research prioritization toward the most promising bioresources of bioactive compounds. The information on *Gordonia* is very limited compared to *Streptomyces*, considering the ecological niches that have been described for them [10] and their average genome size (i.e., ca. 5.30 Mb [10], which is related to the number of BGCs [1]). However, it is captivating to comprehensively explore the diversity and potential of *Gordonia’s* specialized metabolism and its bioprospecting profile.

To our knowledge, there are four published reviews related to the bioprospecting potential of *Gordonia* [8,9,10,29]. These articles comprise narrative reviews mainly focused on *Gordonia* ecological roles. Notably, they agreed on their intriguing biotechnological potential. However, several critical questions for bioprospecting initiatives are not addressed in these compilations (e.g., What is the dimension of the chemical space described so far for *Gordonia*? Are *Gordonia*-derived metabolites like those of other actinomycetes, such as *Streptomyces*? How diverse is the specialized metabolite biosynthetic machinery of *Gordonia*?). Therefore, envisioning that a comprehensive and rigorous synthesis of evidence is required to conceive new and successful studies, we conducted a literature survey to address these issues and collect methodologically *Gordonia*-derived metabolites, including an in silico determination of the promising targets using genome mining.

## 2. Methods

### 2.1. PRISMA-Based Literature Collection

#### 2.1.1. Search Strategy and Study Selection

The literature search was performed following the PRISMA-S guide (Preferred Reporting Items for Systematic reviews and Meta-Analyses literature search extension) [30]. The literature search was carried out in Scopus and Web of Science since these are two of the most extensive databases in terms of scientific literature [31]. The following search equation was developed to find the available literature on compounds isolated from *Gordonia* strains, regardless of the evaluated biological potential: gordonia AND (extract* OR compound OR metabolite OR structure). The first search was conducted on 15 October 2021. A new search was performed on 16 March 2022 to examine those articles published afterward. In each search, the results were limited to original articles using the setting options of each database. Then, the reviews that reached the screening stage were manually excluded. The title and abstract of each article were screened independently and double-blind by two authors using the Rayyan web-based tool [32]. Matches were immediately selected for the full-text reading stage. Discrepancies were agreed upon between the two authors, and a third author made the final decision whenever necessary. Inclusion criteria considered the following: (i) the study involves an actinomycete strain of the genus *Gordonia,* and (ii) the study reports the identification of a compound from a *Gordonia* strain. Studies evaluating the biotransformation capacity of *Gordonia* strains and co-cultures with non-actinomycetes were excluded. The PRISMA-S-based protocol of this systematic review can be consulted in Table S1.

#### 2.1.2. Data Collection

An online form was developed to survey each paper that passed to the full-text reading stage for data collection. A preliminary version of the form was evaluated with seven randomly selected articles independently and double-blind by two authors to build the form. Once this phase was completed, the form was adjusted to incorporate as much data as possible for the aims of this study. The adjusted form was established as the final version to be applied. The articles were coded before being surveyed with the form. The structures of the chemical compounds reported in each paper were coded as follows: article code_*n*, where *n* corresponded to ascending Arabic numerals (i.e., 01, 02… *n*) depending on the number of compounds reported in each paper. A list of the variables incorporated into the final version of the form can be reviewed in Table S2.

### 2.2. Chemoinformatics Analysis

The retrieved chemical structures were converted into SMILES (simplified molecular-input line-entry system) annotation [33] for the subsequent chemical space exploration using the Osiris DataWarrior v5.5.0 software [34] and SwissADME [35]. The building blocks of selected metabolites classified the compounds into alkaloids, amides, terpenoids, and phenylpropanoids. The compounds were filtered and grouped according to the *FragFp* fingerprint descriptor for chemical structure similarity analyses using DataWarrior. The molecular weight (MW), octanol/water partition coefficient (cLogP), number of donor and acceptor hydrogens (*H*-donors, *H*-acceptors), and the drug-likeness were calculated in DataWarrior. Pharmacokinetics (e.g., gastrointestinal absorption, blood-brain barrier permeability, potential inhibition of CYP enzymes, bioavailability) and medicinal chemistry friendliness parameters (e.g., PAINS alerts, Brenk alerts, leadlikeness violations, synthetic accessibility) were estimated through SwissADME.

### 2.3. Comparative Genomics-Based Analysis

#### 2.3.1. Collection of Genome Sequences and Pan-Genome Estimation

All the *Gordonia* genomes used in this study were retrieved from the NCBI’s Genome database. At the time of the search (17 December 2021), the database contained 254 genome assemblies, of which 39 were reference genomes. Detailed information about this dataset can be found in Table S3. The genomes were downloaded in FASTA format (.fna) and annotated through Prokka software [36]. The pan-genome was estimated using the Roary pipeline [37] from the annotated assemblies in GFF3 format. The parameters for the run were as follows: the percentage of isolates in which a gene must be present to be the nucleus was set at 99%, the minimum percentage identity for sequence comparisons performed by BlastP was set at 70% [38] and a maximum number of clusters of 60,000. The Prokka and Roary tools were run using the web-based platform Galaxy at https://usegalaxy.org (accessed on 20 December 2021) [39]. According to Heaps’ law, the pan-genome was classified as open or closed [40,41]. For this, the outputs of (i) the total number of genes and (ii) the number of new genes were used. Roary shows the results of ten random iterations of the input files for each output. The values of κ and α of Heaps’ law equation were calculated by power-law regression analyses [40]. Finally, gene annotation and classification by functional subsystems were performed according to the RAST toolkit using the PATRIC service center [42].

#### 2.3.2. Phylogenomic Analysis

A maximum-likelihood-based tree was inferred from the multi-FASTA alignment of all core genes created in Roary. Gaps in aligned sequences were removed using Gap Strip/Squeeze v2.1.0 with 20% Gap tolerance [43]. The phylogenomic tree was generated in the platform Galaxy using FastTree v2.1.10 [44]. GTR + CAT was set as the nucleotide evolution model, and the other parameters were used as default. The analysis included 39 nucleotide sequences and 692,625 positions in the final data set. The resulting phylogenetic tree in Newick format was uploaded and edited in MEGA v11.0.10 [45]. Finally, from the phylogenetic tree and aligned multiple sequences, they were clustered according to bootstrap support (by default > 90%) and genetic distances (defined from an identity matrix of all core genome sequences computed in BioEdit v7.2.5) using ClusterPickerGUI_1.2.3 [46].

#### 2.3.3. BGC Identification and Similarity Comparison

*Gordonia* genomes were analyzed using antiSMASH (antibiotics and Secondary Metabolite Analysis Shell) v6.0 to predict and annotate BGCs [24]. The resulting GenBank files (.gbk extension) were then used as input for the BiG-SCAPE v1.1.2 pipeline using the default parameters [25]. BiG-SCAPE runs a pairwise analysis of the identified BGCs and defines gene cluster families (GCFs) from a calculated similarity matrix. The resulting networks were imported into Cytoscape v3.9.1 for visualization and analysis.

### 2.4. Data Analysis

Data were entered into Microsoft Excel v2203 spreadsheets for pre-filtering. Descriptive statistical analysis of the data (i.e., calculation of central tendency measures (i.e., mean, median) and dispersion) and figure construction (e.g., histograms, pie charts, boxes, and whiskers) were performed in Graph Pad Prism v9.0.

## 3. Results

### 3.1. General Findings

**Figure 1:**
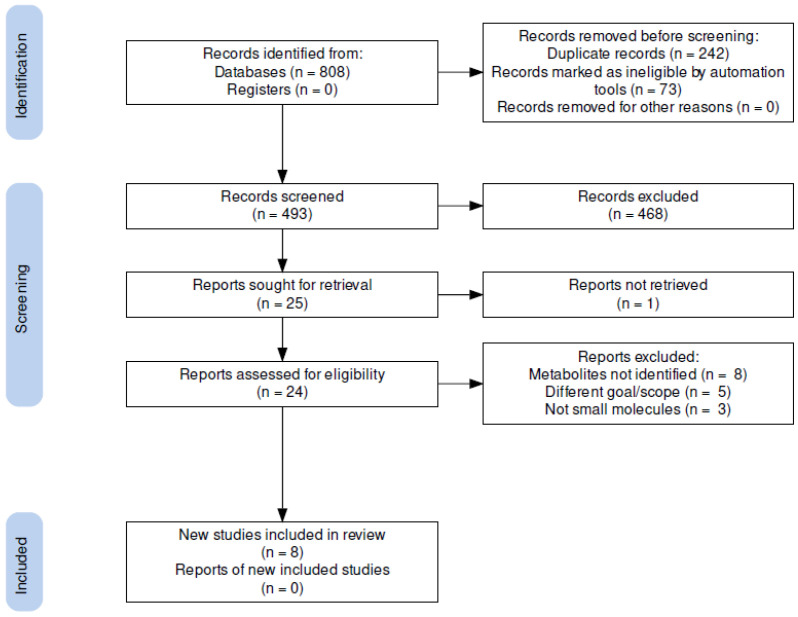
PRISMA flowchart. Flowchart of systematic literature search according to PRISMA-S guidelines. Modified from Page et al. [47]. The flow diagram was constructed using the PRISMA2020 online tool [48]. Detailed information on the literature search is presented in the PRISMA-S checklist in Table S1.

The initial literature search resulted in 493 documents after removing duplicates and other than original articles (Figure 1). After screening titles and abstracts, 468 papers were discarded according to the inclusion/exclusion criteria. Of the remaining twenty-five articles, one could not be retrieved for full-text evaluation, eight did not identify any metabolites, five had a different scope than the one proposed (e.g., optimization of culture conditions), and three involved the evaluation of oligosaccharides (i.e., they were not small molecules). Finally, the review identified eight studies involving specialized metabolites obtained from species of the genus *Gordonia* (Figure 1).

Table 1 provides an overview of the *Gordonia* strains used in the included studies. In five out of eight studies, the strains were defined at the species level, while the others were at the genus level. Although each study used different strains, we observed two strains related to *G. terrae* (i.e., AIST-1 and WA 4-31) and another one (i.e., 647 W.R.1a.05) closely related to *G. terrae* according to its 16S ribosomal gene sequence. 

Most strains (62.5%) were isolated as free-living forms, while the rest were isolated from a host (i.e., *Gordonia* sp. 647 W.R.1a.05, *Gordonia* sp. UA19 and *G. terrae* WA 4-31, isolated from a cone snail, sponge, and cockroach, respectively). The strains were isolated from different environments, a characteristic usually attributed to *Actinobacteria*. Interestingly, given the growing research attention on marine bacteria, three strains were isolated from marine ecosystems (i.e., *G. terrae* AIST-1, *Gordonia* sp. 647 W.R.1a.05, and *Gordonia* sp. UA19).

Regarding the bioprospecting potential, in 5 studies (62.5%), the biological activity was explored, and the antimicrobial capacity was the most frequent (Table 1). In most of the included studies (62.5%), at least one compound was isolated. In fact, in the studies of Schneider et al. [51], Ma et al. [56], Takaichi et al. [53], and Lin et al. [54], 3, 4, 7, and 12 metabolites were reported, respectively. The characteristics of the metabolites retrieved from the studies included in this review are presented in the following section.

### 3.2. Gordonia-Derived Metabolites

**Figure 2:**
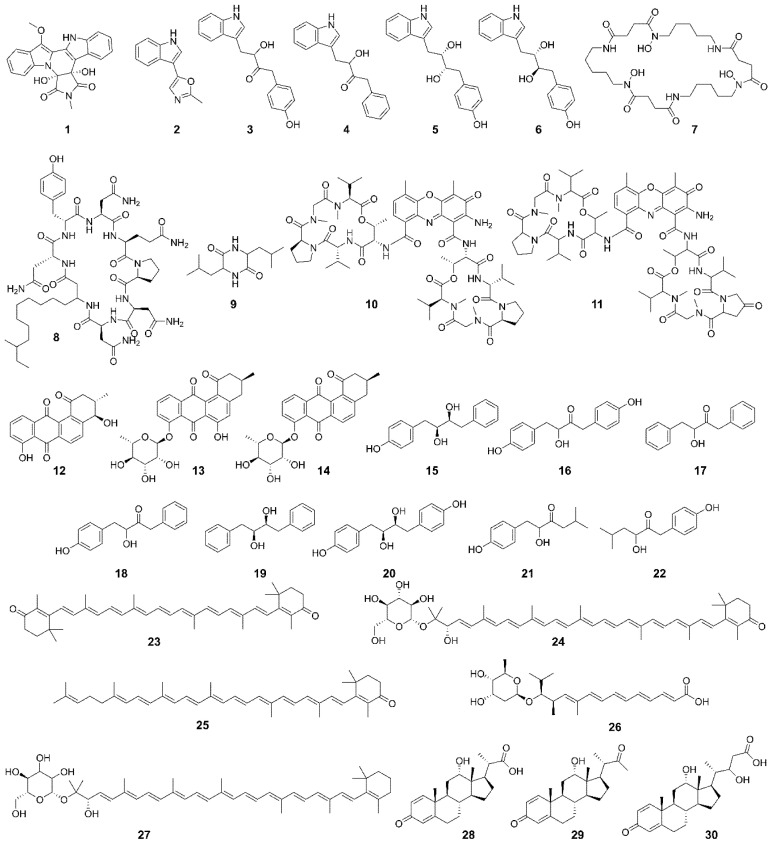
Chemical structures of *Gordonia*-derived compounds. The names, classification according to building blocks, and SMILES are listed in Table S4.

In total, we retrieved 34 metabolites. Four structures were related to carotenoids that were not completely elucidated [53]. Thus, they were not included in our subsequent analyses. The structures of the remaining 30 compounds are presented in Figure 2. According to their building blocks, we found nitrogen-containing compounds such as alkaloids (i.e., **1**–**6**) and amides (i.e., **7**–**11**), as well as phenylpropanoids (i.e., **12**–**20**) and terpenoids (i.e., **21**–**30**).

**Figure 3:**
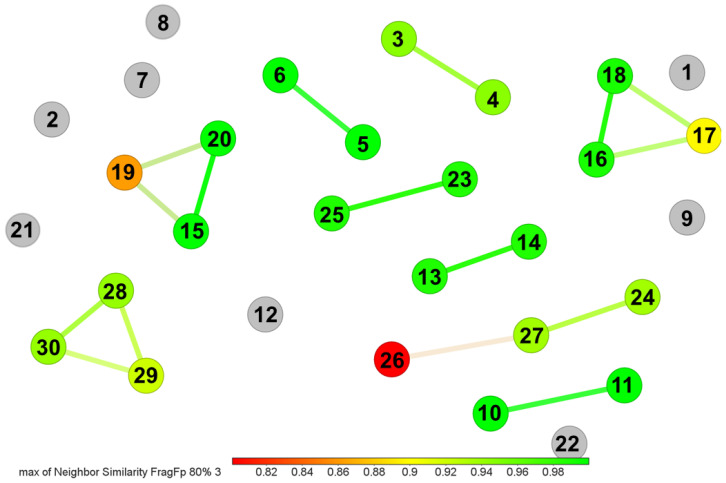
Similarity chart of *Gordonia*-derived compounds. The similarity between compounds was measured using the *FragFp* descriptor. Each dot represents a compound, and those joined by edges form a cluster. Gray dots represent compounds with unique fingerprints within the dataset (*n* = 30). The number at each dot represents the compound ID, as shown in Figure 2. The color scale bar indicates the level of similarity from red (0.8) to green (1.0).

The whole compound set (*n* = 30) was grouped by similarity relationships to explore the chemical diversity (Figure 3). Eight compounds (i.e., **1**, **2**, **7**, **8, 9**, **12**, **21**, and **22**) constitute unique fingerprints and, therefore, could not be clustered. The remaining compounds were distributed in five clusters of pairs, and four clusters comprised three compounds.

**Figure 4:**
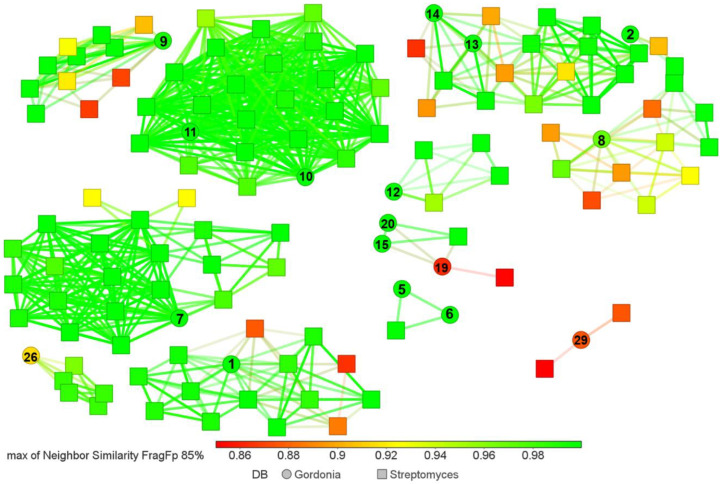
Similarity chart between *Gordonia*-derived compounds and *Streptomcyes*-derived compounds. The similarity between compounds was measured using the *FragFp* descriptor. The dots represent compounds derived from *Gordonia,* and the squares indicate those metabolites derived from *Streptomyces* (obtained from StreptomeDB v3.0 [58]). The color scale bar indicates the level of similarity from red (0.85) to green (1.00).

Since horizontal and vertical gene transfer are involved in the evolution of the biosynthetic pathways of specialized metabolites [57], we aimed to answer whether *Gordonia*-derived compounds are similar to those reported for *Streptomyces.* To this end, we conducted a similarity analysis within the StreptomeDB v3.0 database [58]. In total, 12 clusters involving 17 *Gordonia*-derived compounds and 111 *Streptomyces*-derived compounds were formed (Figure 4). These clusters included different compound types (i.e., alkaloids, amides, phenylpropanoids, and terpenoids). In Figure S1, we highlight some examples of compounds derived from NRPS pathways and indole alkaloids. Within the *Gordonia* compounds, **1**, **5**, **6**, **7**, **10**, **11**, **15**, and **20** have also been isolated from *Streptomyces*. Most of the *Streptomyces*-derived compounds are associated with various biological activities of high interest, such as antibacterial, antifungal, antioxidant, anti-inflammatory, antifouling, neuroprotective, cytotoxic, and enzyme inhibitors (Table S5). This fact indicates the broad untapped potential of *Gordonia’s* specialized metabolism.

**Figure 5:**
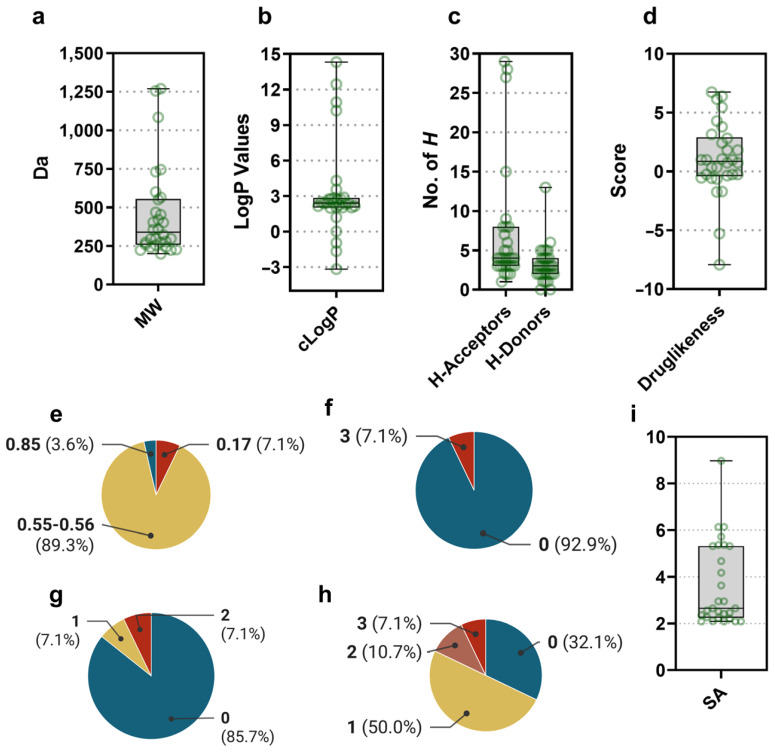
Physicochemical, pharmacokinetics, and medicinal chemistry friendliness evaluation of *Gordonia*-derived compounds. (**a**) Molecular weight (MW; units in Da), (**b**) octanol/water partition coefficient (cLogP; unitless), (**c**) number of hydrogen acceptors and hydrogen donors (*H*-acceptors and *H*-donors; units in numbers of *H*), and (**d**) drug-likeness (unitless; a positive value indicates the occurrence of fragments that are prevalent in commercial drugs) were calculated with Datawarrior v5.5.0. (**e**) Oral bioavailability, (**f**) PAINS alert, (**g**) Brenk alerts, (**h**) Leadlikeness, and (**i**) Synthetic accessibility (SA; unitless) were calculated with SwissADME.

Physicochemical parameters such as molecular mass (MW), octanol/water partition coefficient (clogP), and the number of hydrogen acceptor/donor groups (*H*-acceptors; *H*-donors) play a determining role in drug development [59]. For the *Gordonia*-derived compounds, we found a wide distribution in those values of the respective physicochemical parameters (i.e., MW ranged from 198.22 to 1269.42, cLogP ranged from −3.17 to 14.32, *H*-acceptors ranged 1 to 29, *H*-donors ranged 0 to 13; Figure 5a–c). However, the drug-likeness situated the druggable potential since most of those compounds gathered from *Gordonia* fell into positive values (Figure 5d), indicating its relationship with trade drugs. We also evaluated the pharmacokinetics and medicinal chemistry friendliness by predictive models (Figure 5e–i). Compounds **10** and **11** were excluded, as they exceeded the size limitations of SwissADME. Notably, most of the compounds (98.86%) showed a high probability of oral bioavailability (Figure 5e), which correlates with their degree of compliance with Lipinski’s rules (Table S6). Regarding medicinal chemistry friendliness, the compounds showed encouraging values in parameters such as PAINS, Brenk alerts, and lead-likeness (Figure 5f–h, respectively). However, from the point of view of their synthetic accessibility, no compound was below 2 (very easy), and several compounds (28.57%) scored above 5 (Figure 5i). This aspect is one of the most critical challenges in natural product research. One possible approach to address this issue is using microorganisms as small factories (microbial cell factories) for these high-value-added compounds [60]. Then, the comprehensive understanding of the latent diversity of a microorganism’s specialized metabolism becomes a key factor in advancing along this road. To provide evidence for the usefulness of *Gordonia* as a promising source of bioactive compounds, we further analyze its genomic diversity, emphasizing its specialized metabolism.

### 3.3. Gordonia Pan-Genome

**Figure 6:**
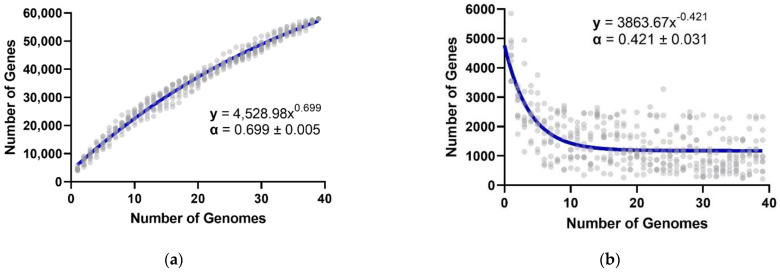
Estimation of the pan-genome for the genus *Gordonia*. (**a**) The number of total genes is plotted as a function of the number of genomes based on ten subsamples per genome. (**b**) The number of new genes is plotted as a function of the number of genomes based on ten subsamples per genome.

**Figure 7:**
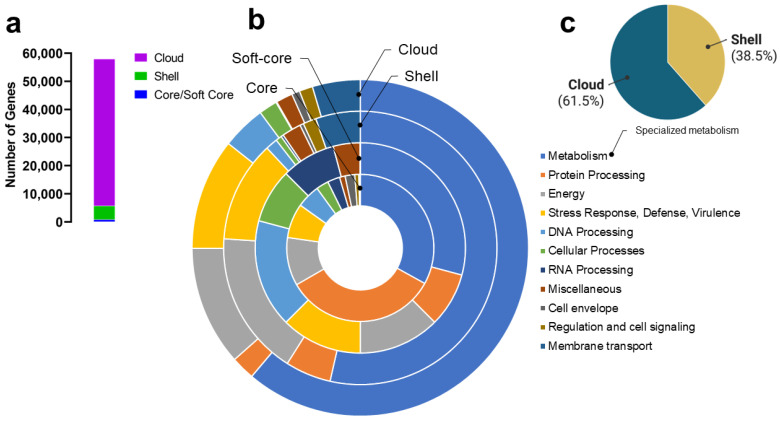
Overview of the assignments to functional subsystems of the *Gordonia* pan-genome. (**a**) Distribution of genes in core/softcore, shell, and cloud genomes. (**b**) Doughnut plot of the functional assignment of genes in the core, softcore, shell, and cloud genome (from the inner to the outer ring, respectively). (**c**) Pie chart of the distribution of genes assigned to specialized metabolism in core and shell genomes (no genes were found within core and softcore genomes).

The number of available genomes of *Gordonia* spp. (*n* = 39) made it possible to explore its gene repertoire. The pan-genome size was estimated at 57,901 genes, and since it is shown to follow Heaps’ law (i.e., α < 1, 0 < γ < 1, Figure 6) [40], it was classified as open. A total of 693 (1.20%) genes comprised the core genome, 170 (0.31%) the softcore, 4822 (8.33%) the shell, and 52,209 (90.17%) the cloud genome (Figure 7a). In the latter, remarkably, 38,209 genes (i.e., 73.18% of cloud genome) were unique, found only once in one of the analyzed genomes. The complete matrix of genes per genome (i.e., per species) can be found in Table S7.

Regarding the functional classification of the predicted genes, the outstanding roles in the core genome were those related to metabolism and protein processing (Figure 7b, 16% each). In the softcore, the distribution was slightly more homogeneous except for genes involved in metabolism. Concerning the shell and cloud genome, the genes assigned to metabolism were clearly preponderant. As expected, most of the genes related to specialized metabolism (i.e., genes that constitute the BGCs) were found in the cloud genome (Figure 7c). To comprehensively analyze the content of BGCs, we mined the *Gordonia* genomes with the specialized metabolism-dedicated antiSMASH v6.0 tool. The results are shown in the section below.

**Figure 8:**
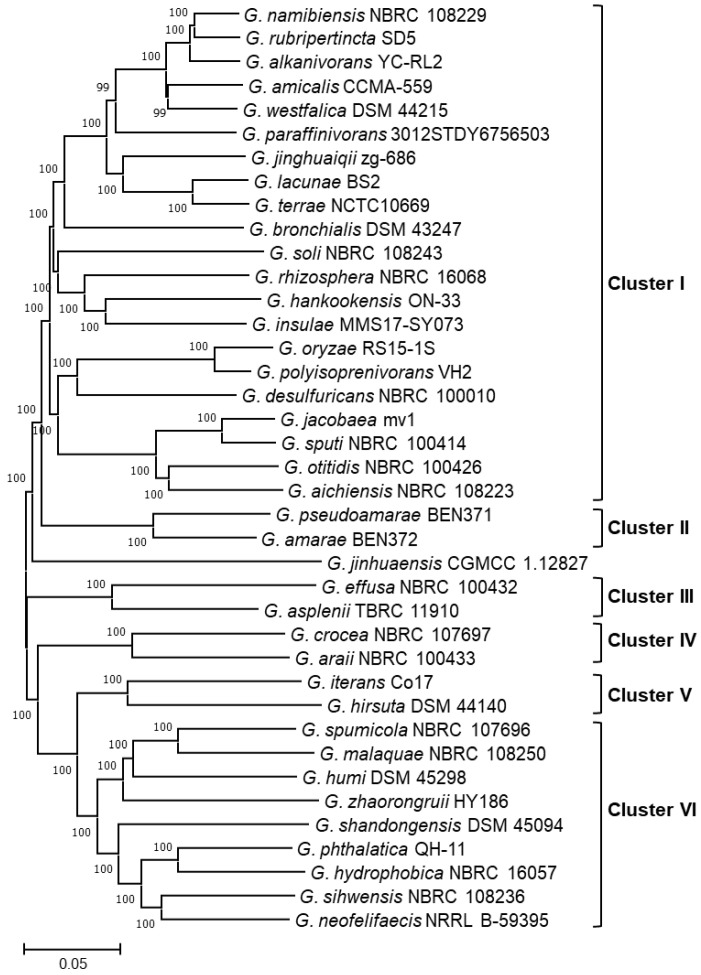
Phylogram of species of the genus *Gordonia*. The phylogenetic tree was inferred by maximum likelihood with FastTree 2 and drawn to scale, with branch lengths measured in the number of substitutions per site. The percentage of bootstrap that supported each node is shown. The analysis included 39 nucleotide sequences (from the 39 *Gordonia* species studied) and 693 markers (692,625 positions in total). Except for *G. jinhuaensis*, the species were grouped into seven clusters (i.e., I, II, III, IV, V, VI, and VII) using ClusterPickerGUI_1.2.3.

A phylogenetic tree was inferred by the maximum-likelihood method to explore the evolutionary relationship based on the core genome of the 39 species of *Gordonia*. According to the bootstrap values, the constructed tree showed strong support for every branch (Figure 8). Considering the similarity between the core genome sequences (Table S8), taxa with a support threshold > 90% and genetic distance < 20.7% were clustered. Seven groups were established as follows: cluster I with nine species, clusters II–V with two species each, cluster VI with seven species, and cluster VII with fourteen species. Interestingly, the species *G. jinhuaensis* did not group into any clusters, with *G. paraffinivorans* and *G. desulfuricans* being the species with the highest level of core genome similarity (i.e., 77.9% and 77.8%, respectively; Table S8).

### 3.4. Gordonia BGC Diversity

**Figure 9:**
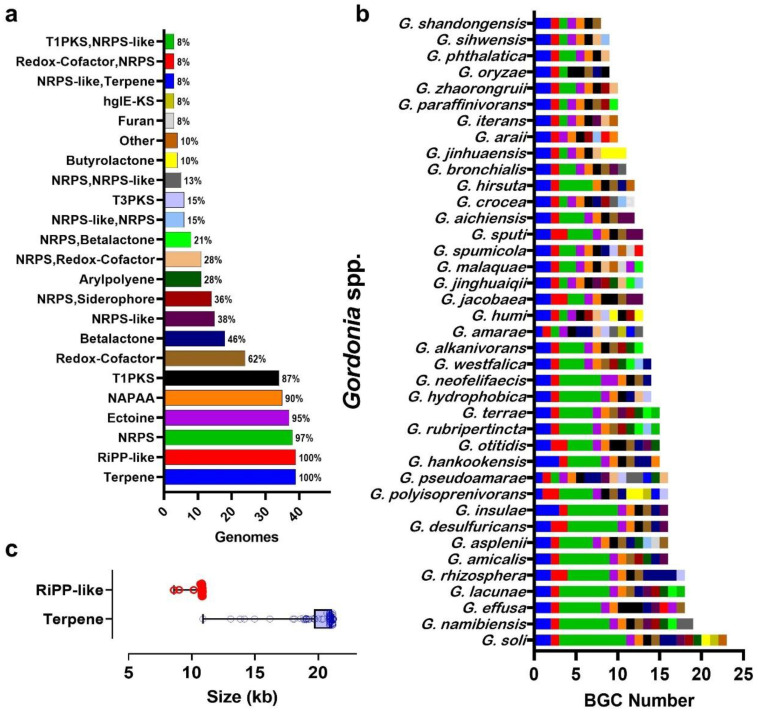
Biosynthetic gene cluster content of species of the genus *Gordonia*. (**a**) The 23 most frequent BGCs (at the front of each bar is the prevalence among the 39 species studied), (**b**) the number of predicted BGCs in the genome of each *Gordonia* species (the color code corresponds to the classification indicated in Figure 9a; for a detailed list see Table S9), (**c**) size in kb of the BGCs occurring in all the genomes studied (i.e., RiPP-like and Terpene).

*Gordonia* genome mining identified 531 clusters classified into 39 different BGC types (Table S9). Figure 9a,b show the 23 most frequent BGCs and the number/type of BGCs detected for each *Gordonia* species, respectively. Regarding the BGC richness, *Gordonia shandongensis* showed the lowest number (i.e., 8), contrasting with *Gordonia soli*, with the highest content, exhibiting finally 23 BGCs (Figure 9b). Among some noteworthy data, the median and mode were 13 BGCs, RiPP-like and Terpenes BGCs were found in all genomes surveyed, and NRPS was the BGCs with the highest prevalence (i.e., 22.6%). Moreover, comparing the size of the RiPP-like and Terpenes clusters (the clusters identified in all the genomes analyzed), the Terpenes clusters varied largely in contrast to RiPP-like (Figure 9c).

**Figure 10:**
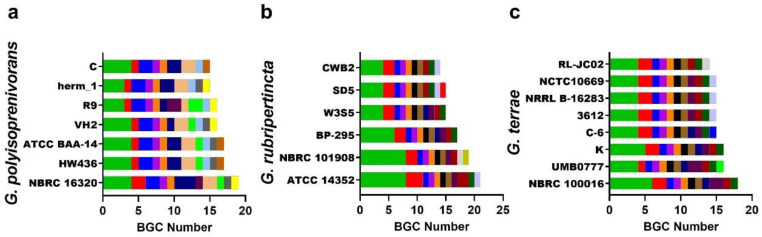
Intraspecies variability of BGC content. (**a**) *G. polyisoprenivorans* strains NBRC 1632, HW436, ATCC BAA-14, VH2, R9, herm_1 and C; (**b**) *G. rubripertincta* strains ATCC 14352, NBRC 101908, BP-295, W3S5, and cwb2; (**c**) *G. terrae* strains NBRC 100016, UMB0777, K, C-6, 3612, NRRL B-16283, NCTC10669, and RL-JC02. The color code corresponds to the same classification indicated in Figure 9a; for a detailed list, see Table S10.

Since BGC content varied considerably (i.e., 15 clusters range) among *Gordonia* species, we interrogated different strains of the same species to see whether BGC content remained relatively stable at the intraspecies level. We selected the species *Gordonia polyisoprenivorans* (seven strains), *G. rubripertincta* (six strains), and *G. terrae* (eight strains), as they were among those with the highest number of genomes reported from different strains. Besides the fact that strains vary drastically less in terms of their content of BGCs (i.e., ranges of four, seven, and four, for *G. polyisoprenivorans*, *G. rubripertincta*, and *G. terrae*, respectively), the types of BGCs are more homogeneous within each species (Figure 10). However, some BGCs are found only in some strains but not in all strains within each species. For instance, *G. rubripertincta* strains NBRC 101908 and SD5 contain unique NRPS hybrid BGCs (Table S10). Notably, NRPS was the cluster with the most copies in all strains (Figure 10).

Finally, we aimed to discover the level of diversity among the related clusters to provide insights into the diversity of the chemical space associated with *Gordonia*. To this end, we use BiG-Scape, which groups those similar BGCs into Gene Cluster Families (GCF). In our analysis, among the 531 BGCs identified, 326 GCFs were defined (i.e., 171 of NRPS, 34 of Terpene, 16 of PKS other, 14 of RiPP, 7 of PKS-I, 6 of PKS-NRPS hybrids, and 78 classifieds as Others), and most of these were singletons (79.75%). BiG-Scape also establishes networks between similar BGCs, which would be involved in the biosynthesis of closely related chemotypes. The established networks comprised 304 nodes (i.e., 57.25% of the identified BGCs) and 1139 edges (Figure 8). The largest number of edges were formed between the nodes of the Others network (i.e., 517 edges including Arypolyene, Betalactone, Ectoine, Redox-Cofactor, and hybrid BGCs; see details in Table S11). The other networks ordered by size were RiPPs (i.e., 243), NRPS (i.e., 144), PKS-I (i.e., 114), Terpene (i.e., 112), other PKSs (i.e., 5) and hybrid PKS-NRP (i.e., 4). Some edges were formed between NRPS clusters and PKS/NRP hybrids. The species contributing the most BGCs to the network nodes were *G. namibiensis* (i.e., 17), *G. rubripertincta* (i.e., 15), *G. amicalis* (i.e., 14), *G. terrae* (i.e., 14), and *G. westfalica* (i.e., 14). In contrast, *G. bronchialis*, *G. crocea,* and *G. araii* contributed the least (i.e., 3, 3, and 2, respectively). Interestingly, despite containing BGCs such as Terpene, ectoine, butyrolactone, RiPP-like, PKS-I, and NRPS, only singletons were identified in *G. jinhuaensis*. This result is consistent with the phylogenetic distance shown by this species (Figure 8).

## 4. Discussion

Natural resource has historically been the primary source of bioactive compounds for developing products with industrial applications, mainly in drug discovery [16]. The research on marine organisms (e.g., sponges, corals, algae) has recently shown exciting potential for new compounds. However, it has been reported that the discovery of novel compounds is more associated with new source organisms [12], among which microorganisms play a preponderant role [61]. Herein we have rigorously scrutinized the bioprospecting potential of actinomycetes of the genus *Gordonia* based on a systematic review of the literature and comparative genomic analysis of 39 species. The review showed that *Gordonia* species are poorly explored bioresources in terms of their specialized metabolism, contrasting with the potential shown in the analysis of their biosynthetic machinery (i.e., BGC content diversity).

The compounds recovered from the literature included structurally diverse metabolites from the main biosynthetic pathways (i.e., nitrogen-containing, phenylpropanoids, and terpenoids), despite coming from only eight strains (Table 1). Interestingly, the strains came from different environments (e.g., marine, terrestrial), including free-living and host-associated forms, which is an indicator of adaptive success and, therefore, the plasticity of their genotype. This diversity of ecological niches of *Gordonia* has already been recognized [10] and, as in other microorganisms, critically relies on developing a sophisticated specialized metabolism [62].

Considering the small number of published articles found, the bioactivity profile of *Gordonia* has been minimal. Nevertheless, compounds that have also been isolated from *Streptomyces,* such as compound **7** (nocardamin), with trypanocidal effect [63], and compound **10** (actinomycin D), which inhibits (Proto-oncogene tyrosine-protein kinase homology and Collagen/ Growth factor receptor-bound protein 2) Shc/Grb2 interaction [64]), serve as examples of bioactivities that remain to be explored further. Additionally, the compounds iturin A (similar to **8**), reported as biosulfurant [65], and (6S,3S)-6-isobutyl-3-methyl-2,5-diketopiperazine (similar to **9**), reported to have antifouling activity [66], contribute to elevating the bioprospecting value of *Gordonia*.

Regarding the drug-like physicochemical characteristics of the *Gordonia*-derived compounds, encouraging features and values were noticed (Figure 5). This is consistent with the fact that several actinomycete-derived compounds have led to approved drugs (e.g., tigecycline, everolimus, telithromycin, miglustat, daptomycin, amrubicin, biapenem, ertapenem, pimecrolimus, and gemtuzumab ozogamicin [67]). Although most of them have been isolated from *Streptomyces*, other genera can also produce highly interesting bioactive compounds. For instance, telithromycin has been isolated from *Saccharopolyspora erythraea* and calicheamicin γ1 (the cytotoxic agent in gemtuzumab ozogamicin) from *Micromonospora echinospora* [61]. Other rare actinomycetes from which bioactive compounds have been isolated are the genera *Actinoallomurus*, *Allostreptomyces*, *Streptosporangium*, *Polymorphospora*, *Lechevalieria*, *Mumia*, *Actinomadura*, and *Amycolatopsis* [68]. This condition makes the rare actinomycetes *Gordonia* spp. another valuable source for discovering small bioactive molecules with these characteristics suitable for drug development. However, as with most natural products, their synthesis is challenging (inferred from the SwissAMDE analysis; Figure 5i). In this regard, the identification/availability of potential microbial factories becomes a critical issue in overcoming this experimental barrier. Therefore, defining the true potential of *Gordonia* to find bioactive specialized metabolites could contribute significantly to the discovery of renewable bioresources of compounds with pharmaceutical interest.

Concerning the pan-genome that we estimated for the genus *Gordonia*, the alpha (α = 0.421 ± 0.031) and gamma (γ = 0.631 ± 0.006) values are like those reported for *Streptomyces* in the work of Caicedo-Montoya et al. (i.e., α = 0.45 ± 0.009 and γ = 0.60 ± 0.002) [38]. However, it should be clarified that our work was not intended to establish the *Gordonia* pan-genome accurately but to assess the diversity of its genotypic repertoire, especially sizing their cloud genome, where specialized metabolism is understood to be contained. Indeed, while no genes involved in specialized metabolism were found in the core or softcore genome, most were found in the cloud genome and some in the shell genome. Thus, given the open pan-genome of *Gordonia*, it was intriguing to explore the richness of the specialized metabolites that might be potentially linked to this genus.

**Figure 11:**
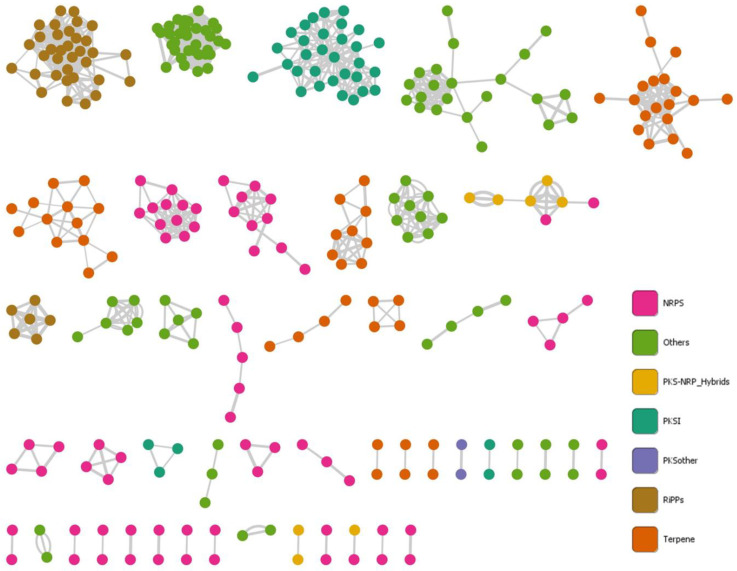
Molecular networks of predicted BGCs in genomes of the genus *Gordonia*. Each node represents a BGC and colors to BGC class. The similarity matrix among BGCs was calculated using BiG-Scape, and the final networks were edited in Cytoscape v3.9.1. A complete and detailed list is available in Table S12.

Tools such as antiSMASH and BiG-Scape have allowed genome mining to become a promising strategy for exploring the latent natural product diversity in each bioresource [25]. Our results revealed that *Gordonia* harbors a high diversity of BGCs (Figure 9). Even though many are closely related (Figure 11), suggesting predominant chemotypes such as terpenoids and RiPP-like, species with unique BGCs (i.e., singletons) were also found. The high prevalence of terpenoid-type BGCs in *Gordonia* is not surprising, given its characteristic carotenoid production [10]. As for RiPP-type BGCs, their abundance in actinomycetes, including *Gordonia*, has recently been reported [69]. Most species we analyzed had a single cluster except for *G. desulfuricans*, *G. jacobaea*, *G. otitidis*, *G. polyisoprenivorans*, *G. rhizosphera,* and *G. sputi*, which involved two detected clusters. Notably, they were all grouped into cluster I (Figure 8) and were located in closely related clades (e.g., *G. jacobaea* and *G. sputi* are on the same branch). Since RiPPs represent a diverse family of compounds with high pharmaceutical interest, they constitute another important metabolite group that could be obtained from *Gordonia* strains.

Regarding unique BGCs, genome mining in *Gordonia* found promising results. When classifying the 531 detected BGCs into families, 23.93% were classified as Other (i.e., different from the well-known NRPS, Terpenes, and PKS, and most of these were singletons). For instance, the 11 BGCs found in *G. jinhuaensis* were singletons. Interestingly, *G. jinhuaensis* was the most distant species among the group analyzed (Figure 8). This outcome supports the fact that, in addition to horizontal transfer, other evolutionary forces, such as functional divergence and de novo assembly, play an essential role in the diversification of BGCs and their consequent chemodiversity [70]. This fact also supports the idea that focusing on more distant (or rare) clades could be a valid strategy for finding novel compounds [12]. Additionally, 227 BGCs did not establish networks, indicating their uniqueness. This event is associated with the similarity of the retrieved compounds since several (26.67%) comprise unique fingerprints. Moreover, when different strains of the same species were analyzed, strains with unique BGCs were found, and the diversity could even be sustained at the intraspecies level. This fact has been reported in other genera, including *Streptomyces* [1].

## 5. Conclusions

Although there is a lack of bioprospecting research on the genus *Gordonia*, it represents a promising bioresource for discovering high-value-added microbial natural products. Several *Gordonia*-derived compounds had also been obtained from *Streptomyces* and showed diverse bioactivity potential. In addition to demonstrating applications that remain to be evaluated, *Gordonia* may be a source for challenging *Streptomyces*-derived compounds. Genome mining findings showed that species of the genus *Gordonia* harbor diverse types of BGCs, which in addition to Terpenes, RiPP, NRPS, and PKS, included novel motifs which could be associated with innovative compounds or scaffolds. *Gordonia* is a rare actinomycete of high value for bioprospecting type studies. It is important to note that *Gordonia* is a relatively new genus, and most species have been described since 2000 (74.47%), so as new species are reported, the chemical space associated with *Gordonia* could be substantially expanded in further studies.

## Figures and Tables

**Table 1:** Summary of data collected from the articles included.

*Gordonia* Strain	Isolation Features		Natural Product Type	Bioactivity Potential	Ref. ^b^
	Habitat ^a^	Country			
*G. jacobaea* CECT 5282	T_FL_	Spain	Extract	N/A ^c^	[49]
*G. rubripertincta* CWB2 (DSM 46758)	T_FL_	Germany	Compound	N/A	[50]
*G. australis* Acta 2299	FW_FL_	Australia	Compound	Steroid receptors binding	[51]
*Gordonia* sp. KMC005	FW_FL_	Republic of Korea	Compound	Antimicrobial	[52]
*G. terrae* AIST-1	M_FL_	Japan	Compound	N/A	[53]
*Gordonia* sp. 647 W.R.1a.05	M_HA_	Philippines	Compound	Neuroactive	[54]
*Gordonia* sp. UA19	M_HA_	Egypt	Extract	Antimicrobial	[55]
*G. terrae* WA 4-31	T_HA_	China	Compound	Antimicrobial, Cytotoxic	[56]

^a^ Terrestrial free-living (T_FL_), Freshwater free-living (F_FL_), Marine free-living (M_FL_), Marine host-associated (M_HA_), and Terrestrial host-associated (T_HA_). ^b^ Reference. ^c^ Not available.

## Data Availability

The data used to support the findings of this study are provided within this article. However, any required further information can be provided by the corresponding author upon request.

## Supplementary Materials

The following supporting information can be downloaded at: https://www.mdpi.com/article/10.3390/biotech11040053/s1, Figure S1: *Gordonia*-derived compounds similar to *Streptomyces*-derived compounds; Table S1: PRISMA-S Checklist; Table S2: List of variables used in the form to survey each article that passed the screening phase; Table S3: Database of *Gordonia* genomes used in this study; Table S4: Data on *Gordonia*-derived compounds retrieved from the articles included in the systematic review; Table S5: Clustering of *Gordonia*-derived compounds by similarity to *Streptomyces*-derived compounds; Table S6: Datawarrior and SwissADME physicochemical parameters; Table S7: Roary presence/absence table; Table S8: Similarity matrix between the core genome sequences; Table S9: Results of mining *Gordonia* reference genomes by the antiSMASH tool; Table S10: Results of mining *G. polyisoprenivorans, G. rubripertincta,* and *G. terrae* genomes by the antiSMASH tool; Table S11: Clustering by family of BGCs detected in *Gordonia* genomes by the BiG-Scape tool; Table S12: Network data by similarity of BGCs detected in *Gordonia* genomes built by the BiG-Scape tool.

## References

[1] Wei B., Du A.Q., Zhou Z.Y., Lai C., Yu W.C., Yu J.B., Yu Y.L., Chen J.W., Zhang H.W., Xu X.W. (2021). An Atlas of Bacterial Secondary Metabolite Biosynthesis Gene Clusters. Environ. Microbiol..

[2] El-Naggar N.E.-A. (2021). Streptomyces-Based Cell Factories for Production of Biomolecules and Bioactive Metabolites. Microbial Cell Factories Engineering for Production of Biomolecules.

[3] Sánchez-Suárez J., Coy-Barrera E., Villamil L., Díaz L. (2020). Streptomyces-Derived Metabolites with Potential Photoprotective Properties—A Systematic Literature Review and Meta-Analysis on the Reported Chemodiversity. Molecules.

[4] Choi S.-S., Kim H.-J., Lee H.-S., Kim P., Kim E.-S. (2015). Genome Mining of Rare Actinomycetes and Cryptic Pathway Awakening. Process Biochem..

[5] Jose P.A., Sivakala K.K., Jha B. (2019). Non-Streptomyces Actinomycetes and Natural Products: Recent Updates. Studies in Natural Products Chemistry.

[6] Subramani R., Sipkema D. (2019). Marine Rare Actinomycetes: A Promising Source of Structurally Diverse and Unique Novel Natural Products. Mar. Drugs.

[7] Stackebrandt E., Smida J., Collins M.D. (1988). Evidence of Phylogenetic Heterogeneity within the Genus Rhodococcus: Revival of the Genus Gordona (Tsukamura). J. Gen. Appl. Microbiol..

[8] Arenskötter M., Bröker D., Steinbüchel A. (2004). Biology of the Metabolically Diverse Genus Gordonia. Appl. Environ. Microbiol..

[9] Andalibi F., Fatahi-Bafghi M. (2017). Gordonia: Isolation and Identification in Clinical Samples and Role in Biotechnology. Folia Microbiol..

[10] Sowani H., Kulkarni M., Zinjarde S. (2018). An Insight into the Ecology, Diversity and Adaptations of Gordonia Species. Crit. Rev. Microbiol..

[11] O’Brien P.A., Webster N.S., Miller D.J., Bourne D.G. (2019). Host-Microbe Coevolution: Applying Evidence from Model Systems to Complex Marine Invertebrate Holobionts. MBio.

[12] Voser T.M., Campbell M.D., Carroll A.R. (2022). How Different Are Marine Microbial Natural Products Compared to Their Terrestrial Counterparts?. Nat. Prod. Rep..

[13] Hug J.J., Krug D., Müller R. (2020). Bacteria as Genetically Programmable Producers of Bioactive Natural Products. Nat. Rev. Chem..

[14] Rosero-Chasoy G., Rodríguez-Jasso R.M., Aguilar C.N., Buitrón G., Chairez I., Ruiz H.A. (2021). Microbial Co-Culturing Strategies for the Production High Value Compounds, a Reliable Framework towards Sustainable Biorefinery Implementation—An Overview. Bioresour. Technol..

[15] Verstraete W., Yanuka-Golub K., Driesen N., De Vrieze J. (2022). Engineering Microbial Technologies for Environmental Sustainability: Choices to Make. Microb. Biotechnol..

[16] Newman D.J., Cragg G.M. (2020). Natural Products as Sources of New Drugs over the Nearly Four Decades from 01/1981 to 09/2019. J. Nat. Prod..

[17] McCauley E.P., Piña I.C., Thompson A.D., Bashir K., Weinberg M., Kurz S.L., Crews P. (2020). Highlights of Marine Natural Products Having Parallel Scaffolds Found from Marine-Derived Bacteria, Sponges, and Tunicates. J. Antibiot..

[18] Aguilar A., Twardowski T., Wohlgemuth R. (2019). Bioeconomy for Sustainable Development. Biotechnol. J..

[19] Akinsemolu A.A. (2018). The Role of Microorganisms in Achieving the Sustainable Development Goals. J. Clean. Prod..

[20] Haines A. (2021). Health in the Bioeconomy. Lancet Planet. Heal..

[21] Wohlgemuth R., Twardowski T., Aguilar A. (2021). Bioeconomy Moving forward Step by Step—A Global Journey. N. Biotechnol..

[22] Timmis K., de Lorenzo V., Verstraete W., Ramos J.L., Danchin A., Brüssow H., Singh B.K., Timmis J.K. (2017). The Contribution of Microbial Biotechnology to Economic Growth and Employment Creation. Microb. Biotechnol..

[23] Neergheen-Bhujun V., Awan A.T., Baran Y., Bunnefeld N., Chan K., dela Cruz T.E., Egamberdieva D., Elsässer S., Johnson M.V., Komai S. (2017). Biodiversity, Drug Discovery, and the Future of Global Health: Introducing the Biodiversity to Biomedicine Consortium, a Call to Action. J. Glob. Health.

[24] Blin K., Shaw S., Kloosterman A.M., Charlop-Powers Z., van Wezel G.P., Medema M.H., Weber T. (2021). AntiSMASH 6.0: Improving Cluster Detection and Comparison Capabilities. Nucleic Acids Res..

[25] Navarro-Muñoz J.C., Selem-Mojica N., Mullowney M.W., Kautsar S.A., Tryon J.H., Parkinson E.I., De Los Santos E.L.C., Yeong M., Cruz-Morales P., Abubucker S. (2020). A Computational Framework to Explore Large-Scale Biosynthetic Diversity. Nat. Chem. Biol..

[26] Chevrette M.G., Gavrilidou A., Mantri S., Selem-Mojica N., Ziemert N., Barona-Gómez F. (2021). The Confluence of Big Data and Evolutionary Genome Mining for the Discovery of Natural Products. Nat. Prod. Rep..

[27] Medema M.H., Kottmann R., Yilmaz P., Cummings M., Biggins J.B., Blin K., de Bruijn I., Chooi Y.H., Claesen J., Coates R.C. (2015). Minimum Information about a Biosynthetic Gene Cluster. Nat. Chem. Biol..

[28] Soldatou S., Eldjarn G.H., Huerta-Uribe A., Rogers S., Duncan K.R. (2019). Linking Biosynthetic and Chemical Space to Accelerate Microbial Secondary Metabolite Discovery. FEMS Microbiol. Lett..

[29] Drzyzga O. (2012). The Strengths and Weaknesses of Gordonia: A Review of an Emerging Genus with Increasing Biotechnological Potential. Crit. Rev. Microbiol..

[30] Rethlefsen M.L., Kirtley S., Waffenschmidt S., Ayala A.P., Moher D., Page M.J., Koffel J.B. (2021). PRISMA-S: An Extension to the PRISMA Statement for Reporting Literature Searches in Systematic Reviews. Syst. Rev..

[31] Aghaei Chadegani A., Salehi H., Md Yunus M.M., Farhadi H., Fooladi M., Farhadi M., Ale Ebrahim N. (2013). A Comparison between Two Main Academic Literature Collections: Web of Science and Scopus Databases. Asian Soc. Sci..

[32] Ouzzani M., Hammady H., Fedorowicz Z., Elmagarmid A. (2016). Rayyan—A Web and Mobile App for Systematic Reviews. Syst. Rev..

[33] Weininger D. (1988). SMILES, a Chemical Language and Information System. 1. Introduction to Methodology and Encoding Rules. J. Chem. Inf. Model..

[34] Sander T., Freyss J., Von Korff M., Rufener C. (2015). DataWarrior: An Open-Source Program for Chemistry Aware Data Visualization and Analysis. J. Chem. Inf. Model..

[35] Daina A., Michielin O., Zoete V. (2017). SwissADME: A Free Web Tool to Evaluate Pharmacokinetics, Drug-Likeness and Medicinal Chemistry Friendliness of Small Molecules. Sci. Rep..

[36] Seemann T. (2014). Prokka: Rapid Prokaryotic Genome Annotation. Bioinformatics.

[37] Page A.J., Cummins C.A., Hunt M., Wong V.K., Reuter S., Holden M.T.G., Fookes M., Falush D., Keane J.A., Parkhill J. (2015). Roary: Rapid Large-Scale Prokaryote Pan Genome Analysis. Bioinformatics.

[38] Caicedo-Montoya C., Manzo-Ruiz M., Ríos-Estepa R. (2021). Pan-genome of the genus *Streptomyces* and prioritization of biosynthetic gene clusters with potential to produce antibiotic compounds. Front. Microbiol..

[39] Afgan E., Baker D., Batut B., van den Beek M., Bouvier D., Čech M., Chilton J., Clements D., Coraor N., Grüning B.A. (2018). The Galaxy Platform for Accessible, Reproducible and Collaborative Biomedical Analyses: 2018 Update. Nucleic Acids Res..

[40] Tettelin H., Riley D., Cattuto C., Medini D. (2008). Comparative Genomics: The Bacterial Pan-Genome. Curr. Opin. Microbiol..

[41] Costa S.S., Guimarães L.C., Silva A., Soares S.C., Baraúna R.A. (2020). First Steps in the Analysis of Prokaryotic Pan-Genomes. Bioinform. Biol. Insights.

[42] Davis J.J., Wattam A.R., Aziz R.K., Brettin T., Butler R., Butler R.M., Chlenski P., Conrad N., Dickerman A., Dietrich E.M. (2020). The PATRIC Bioinformatics Resource Center: Expanding Data and Analysis Capabilities. Nucleic Acids Res..

[43] Los Alamos National Laboratory Gap Strip/Squeeze v2.1.0. http://www.hiv.lanl.gov/content/sequence/GAPSTREEZE/gap.html.

[44] Price M.N., Dehal P.S., Arkin A.P. (2010). FastTree 2—Approximately Maximum-Likelihood Trees for Large Alignments. PLoS ONE.

[45] Tamura K., Stecher G., Kumar S. (2021). MEGA11: Molecular Evolutionary Genetics Analysis Version 11. Mol. Biol. Evol..

[46] Ragonnet-Cronin M., Hodcroft E., Hué S., Fearnhill E., Delpech V., Brown A.J.L., Lycett S. (2013). Automated Analysis of Phylogenetic Clusters. BMC Bioinform..

[47] Page M.J., McKenzie J.E., Bossuyt P.M., Boutron I., Hoffmann T.C., Mulrow C.D., Shamseer L., Tetzlaff J.M., Akl E.A., Brennan S.E. (2021). The PRISMA 2020 Statement: An Updated Guideline for Reporting Systematic Reviews. BMJ.

[48] Haddaway N.R., Pritchard C.C., McGuinness L.A. (2021). PRISMA2020: R Package and ShinyApp for Producing PRISMA 2020 Compliant Flow Diagrams, Version 0.0.2. http://irep.ntu.ac.uk/id/eprint/43397/.

[49] De Miguel T., Sieiro C., Poza M., Villa T.G. (2001). Analysis of Canthaxanthin and Related Pigments from Gordonia Jacobaea Mutants. J. Agric. Food Chem..

[50] Schwabe R., Senges C.H.R., Bandow J.E., Heine T., Lehmann H., Wiche O., Schlömann M., Levicán G., Tischler D. (2020). Cultivation Dependent Formation of Siderophores by Gordonia Rubripertincta CWB2. Microbiol. Res..

[51] Schneider K., Graf E., Irran E., Nicholson G., Stainsby F.M., Goodfellow M., Borden S.A., Keller S., Süssmuth R.D., Fiedler H.P. (2008). Bendigoles A∼C, New Steroids from Gordonia Australis Acta 2299. J. Antibiot..

[52] Park H.B., Park J.-S., Lee S.I., Shin B., Oh D.-C., Kwon H.C. (2017). Gordonic Acid, a Polyketide Glycoside Derived from Bacterial Coculture of Streptomyces and Gordonia Species. J. Nat. Prod..

[53] Takaichi S., Maoka T., Akimoto N., Carmona M.L., Yamaoka Y. (2008). Carotenoids in a Corynebacterineae, Gordonia Terrae AIST-1: Carotenoid Glucosyl Mycoloyl Esters. Biosci. Biotechnol. Biochem..

[54] Lin Z., Marett L., Hughen R.W., Flores M., Forteza I., Ammon M.A., Concepcion G.P., Espino S., Olivera B.M., Rosenberg G. (2013). Neuroactive Diol and Acyloin Metabolites from Cone Snail-Associated Bacteria. Bioorganic Med. Chem. Lett..

[55] Shamikh Y.I., El Shamy A.A., Gaber Y., Abdelmohsen U.R., Madkour H.A., Horn H., Hassan H.M., Elmaidomy A.H., Alkhalifah D.H.M., Hozzein W.N. (2020). Actinomycetes from the Red Sea Sponge Coscinoderma Mathewsi: Isolation, Diversity, and Potential for Bioactive Compounds Discovery. Microorganisms.

[56] Ma Y., Xu M., Liu H., Yu T., Guo P., Liu W., Jin X. (2021). Antimicrobial Compounds Were Isolated from the Secondary Metabolites of Gordonia, a Resident of Intestinal Tract of Periplaneta Americana. AMB Express.

[57] Osbourn A. (2010). Secondary Metabolic Gene Clusters: Evolutionary Toolkits for Chemical Innovation. Trends Genet..

[58] Moumbock A.F.A., Gao M., Qaseem A., Li J., Kirchner P.A., Ndingkokhar B., Bekono B.D., Simoben C.V., Babiaka S.B., Malange Y.I. (2021). StreptomeDB 3.0: An Updated Compendium of Streptomycetes Natural Products. Nucleic Acids Res..

[59] Gleeson M.P., Hersey A., Montanari D., Overington J. (2011). Probing the Links between in vitro Potency, ADMET and Physicochemical Parameters. Nat. Rev. Drug Discov..

[60] Gohil N., Bhattacharjee G., Singh V. (2021). An Introduction to Microbial Cell Factories for Production of Biomolecules. Microbial Cell Factories Engineering for Production of Biomolecules.

[61] Abdel-Razek A.S., El-Naggar M.E., Allam A., Morsy O.M., Othman S.I. (2020). Microbial Natural Products in Drug Discovery. Processes.

[62] O’Brien J., Wright G.D. (2011). An Ecological Perspective of Microbial Secondary Metabolism. Curr. Opin. Biotechnol..

[63] Conti R., Chagas F.O., Caraballo-Rodriguez A.M., da Paixão Melo W.G., do Nascimento A.M., Cavalcanti B.C., de Moraes M.O., Pessoa C., Costa-Lotufo L.V., Krogh R. (2016). Endophytic Actinobacteria from the Brazilian Medicinal Plant Lychnophora *Ericoides* Mart. and the Biological Potential of Their Secondary Metabolites. Chem. Biodivers..

[64] Kim H.-K., Jeong M.-J., Kong M.-Y., Han M.Y., Son K.-H., Kim H.M., Hong S.H., Kwon B.-M. (2005). Inhibition of Shc/Grb2 Protein–Protein Interaction Suppresses Growth of B104-1-1 Tumors Xenografted in Nude Mice. Life Sci..

[65] Rodrigues L., Banat I.M., Teixeira J., Oliveira R. (2006). Biosurfactants: Potential Applications in Medicine. J. Antimicrob. Chemother..

[66] Cho J.Y., Kang J.Y., Hong Y.K., Baek H.H., Shin H.W., Kim M.S. (2012). Isolation and Structural Determination of the Antifouling Diketopiperazines from Marine-Derived Streptomyces Praecox 291-11. Biosci. Biotechnol. Biochem..

[67] Ma R., Karthik L. (2022). Pharmacology of FDA-Approved Medicines from Actinobacteria. Actinobacteria.

[68] Takahashi Y., Nakashima T. (2018). Actinomycetes, an Inexhaustible Source of Naturally Occurring Antibiotics. Antibiotics.

[69] Poorinmohammad N., Bagheban-Shemirani R., Hamedi J. (2019). Genome Mining for Ribosomally Synthesised and Post-Translationally Modified Peptides (RiPPs) Reveals Undiscovered Bioactive Potentials of Actinobacteria. Antonie Van Leeuwenhoek.

[70] Rokas A., Mead M.E., Steenwyk J.L., Raja H.A., Oberlies N.H. (2020). Biosynthetic Gene Clusters and the Evolution of Fungal Chemodiversity. Nat. Prod. Rep..

