# Supplementary material for: Specialized Metabolism of Gordonia Genus: An Integrated Survey on Chemodiversity Combined with a Comparative Genomics-Based Analysis
Source: BioTech (Basel). 2022 Nov 21;11(4):53. doi: 10.3390/biotech11040053 (PMC9680422; doi:10.3390/biotech11040053)
Supplement: Supplementary file 1 [file biotech-11-00053-s001.zip › biotech-1979252-supplementary.pdf]

Supplementary Materials: Specialized Metabolism of *Gordonia* Genus: An Integrated Survey on Chemodiversity Combined with a Comparative Genomics-Based Analysis

Jeysson Sánchez-Suárez, Luis Díaz, Ericsson Coy-Barrera, and Luisa Villamil

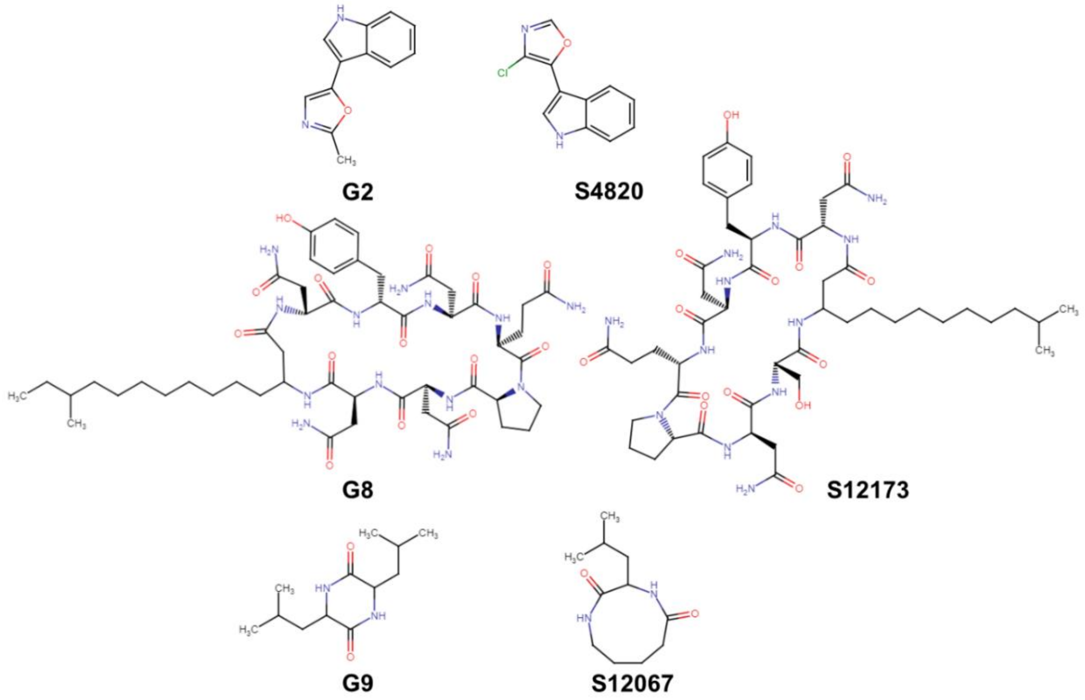

Figure S1. *Gordonia*-derived compounds similar to *Streptomyces*-derived compounds. The preceding letter of each ID indicates the isolation source (i.e., G=Gordonia, S=Streptomyces). G2= Pimprinine, G8= Mojavensin A, and G9= cyclo(Leu-Leu). S4820= Streptochlorin, S12174= Iturin A, and S12067= 1,4-Diaza-2,5-dioxo-3-isobutylbicyclo[4.3.0]nonane.

Table S1. PRISMA-S Checklist.

Section/topic	#	Checklist item	Location(s) Reported
**INFORMATION SOURCES AND METHODS**			
Database name	1	Name each individual database searched, stating the platform for each.	3
Multi-database searching	2	If databases were searched simultaneously on a single platform, state the name of the platform, listing all of the databases searched.	N/A
Study registries	3	List any study registries searched.	N/A
Online resources and browsing	4	Describe any online or print source purposefully searched or browsed (e.g., tables of contents, print conference proceedings, web sites), and how this was done.	N/A
Citation searching	5	Indicate whether cited references or citing references were examined, and describe any methods used for locating cited/citing references (e.g., browsing reference lists, using a citation index, setting up email alerts for references citing included studies).	N/A
Contacts	6	Indicate whether additional studies or data were sought by contacting authors, experts, manufacturers, or others.	N/A
Other methods	7	Describe any additional information sources or search methods used.	N/A
**SEARCH STRATEGIES**			
Full search strategies	8	Include the search strategies for each database and information source, copied and pasted exactly as run.	3
Limits and restrictions	9	Specify that no limits were used, or describe any limits or restrictions applied to a search (e.g., date or time period, language, study design) and provide justification for their use.	3
Search filters	10	Indicate whether published search filters were used (as originally designed or modified), and if so, cite the filter(s) used.	N/A
Prior work	11	Indicate when search strategies from other literature reviews were adapted or reused for a substantive part or all of the search, citing the previous review(s).	N/A
Updates	12	Report the methods used to update the search(es) (e.g., rerunning searches, email alerts).	3
Dates of searches	13	For each search strategy, provide the date when the last search occurred.	3
**PEER REVIEW**			
Peer review	14	Describe any search peer review process.	N/A

MANAGING RECORDS			
Total Records	15	Document the total number of records identified from each database and other information sources.	5
Deduplication	16	Describe the processes and any software used to deduplicate records from multiple database searches and other information sources.	3

PRISMA-S: An Extension to the PRISMA Statement for Reporting Literature Searches in Systematic Reviews

Rethlefsen ML, Kirtley S, Waffenschmidt S, Ayala AP, Moher D, Page MJ, Koffel JB, PRISMA-S Group.

Last updated February 27, 2020.

Table S2. List of variables used in the form to survey each article that passed the screening phase.

No.	Variable
1	Journal name
2	Year of publication
3	Name of paper
4	Last name of corresponding author
5	First name of corresponding author
6	Country of corresponding author
7	Institute of corresponding author
8	Country of sample collection
9	Isolation source
10	Name of macroorganism source
11	Gordonia species ID name
12	Identification method
13	NCBI Accession number
14	Culture type
15	Culture medium name
16	Described metabolite extraction process?
17	Extraction solvent
18	Metabolite isolation
19	Number of compounds isolated
20	Isolation methodology
21	Metabolite ID
22	Metabolite name
23	Bioactivity evaluated?
24	What bioactivities?
25	Assay type
26	Methodology notes (related to bioactivity).
27	Biosynthesis pathway
28	Biosynthesis gene cluster suggestion

Table S3. Database of Gordonia genomes used in this study.

No.	Organism Name	Assembly Accession	Assembly Stats Total Sequence Length	Assembly Level	Assembly Submission Date	Reference Genome
1	Gordonia aichiensis NBRC 108223	GCF_000332975.1	5092029	Contig	11/01/2013	Yes
2	Gordonia aichiensis NBRC 108223	GCA_000332975.1	5092029	Contig	11/01/2013	No
3	Gordonia ajococcus	GCF_012974285.1	5090254	Complete Genome	5/05/2020	No
4	Gordonia ajococcus	GCA_012974285.1	5090254	Complete Genome	5/05/2020	No
5	Gordonia alkanivorans	GCF_004011905.1	4979656	Complete Genome	11/01/2019	Yes
6	Gordonia alkanivorans	GCA_004011905.1	4979656	Complete Genome	11/01/2019	No
7	Gordonia alkanivorans CGMCC 6845	GCF_000503935.1	5025131	Scaffold	4/12/2013	No
8	Gordonia alkanivorans CGMCC 6845	GCA_000503935.1	5025131	Scaffold	4/12/2013	No
9	Gordonia alkanivorans NBRC 16433	GCF_000225505.1	5071550	Contig	16/08/2011	No
10	Gordonia alkanivorans NBRC 16433	GCA_000225505.1	5071550	Contig	16/08/2011	No
11	Gordonia alkanivorans s104	GCF_000529675.1	5180580	Scaffold	15/01/2014	No
12	Gordonia alkanivorans s104	GCA_000529675.1	5180580	Scaffold	15/01/2014	No
13	Gordonia amarae	GCF_009914515.1	5291543	Complete Genome	22/01/2020	Yes
14	Gordonia amarae	GCF_009914495.1	5395088	Complete Genome	22/01/2020	No
15	Gordonia amarae	GCA_009914495.1	5395088	Complete Genome	22/01/2020	No
16	Gordonia amarae	GCA_009914515.1	5291543	Complete Genome	22/01/2020	No
17	Gordonia amarae NBRC 15530	GCF_000241345.1	5306375	Contig	25/11/2011	No
18	Gordonia amarae NBRC 15530	GCA_000241345.2	5306375	Contig	25/11/2011	No
19	Gordonia amicalis	GCF_012395955.1	4925831	Contig	15/04/2020	No
20	Gordonia amicalis	GCF_014050135.1	4976699	Scaffold	3/08/2020	No
21	Gordonia amicalis	GCA_012395955.1	4925831	Contig	15/04/2020	No

No.	Organism Name	Assembly Accession	Assembly Stats Total Sequence Length	Assembly Level	Assembly Submission Date	Reference Genome
22	Gordonia amicalis	GCA_014050135.1	4976699	Scaffold	3/08/2020	No
23	Gordonia amicalis CCMA-559	GCF_000472025.1	5179574	Contig	27/09/2013	Yes
24	Gordonia amicalis CCMA-559	GCA_000472025.1	5179574	Contig	27/09/2013	No
25	Gordonia amicalis NBRC 100051 = JCM 11271	GCF_000332995.1	4915471	Contig	11/01/2013	No
26	Gordonia amicalis NBRC 100051 = JCM 11271	GCA_000332995.1	4915471	Contig	11/01/2013	No
27	Gordonia araii NBRC 100433	GCF_000241265.1	3906554	Contig	25/11/2011	Yes
28	Gordonia araii NBRC 100433	GCF_013004535.1	3911123	Contig	10/05/2020	No
29	Gordonia araii NBRC 100433	GCA_000241265.2	3906554	Contig	25/11/2011	No
30	Gordonia araii NBRC 100433	GCA_013004535.1	3911123	Contig	10/05/2020	No
31	Gordonia asplenii	GCF_012933505.1	6646044	Contig	29/04/2020	Yes
32	Gordonia asplenii	GCA_012933505.1	6646044	Contig	29/04/2020	No
33	Gordonia bronchialis	GCF_009730435.1	5298960	Complete Genome	5/12/2019	No
34	Gordonia bronchialis	GCF_019930645.1	5307108	Complete Genome	12/09/2021	No
35	Gordonia bronchialis	GCF_020731255.1	5288120	Scaffold	2/11/2021	No
36	Gordonia bronchialis	GCF_900450805.1	5326567	Contig	1/08/2018	No
37	Gordonia bronchialis	GCA_009730435.1	5298960	Complete Genome	5/12/2019	No
38	Gordonia bronchialis	GCA_019930645.1	5307108	Complete Genome	12/09/2021	No
39	Gordonia bronchialis	GCA_020731255.1	5288120	Scaffold	2/11/2021	No
40	Gordonia bronchialis	GCA_900450805.1	5326567	Contig	1/08/2018	No
41	Gordonia bronchialis DSM 43247	GCF_000024785.1	5290012	Complete Genome	27/10/2009	Yes
42	Gordonia bronchialis DSM 43247	GCA_000024785.1	5290012	Complete Genome	27/10/2009	No
43	Gordonia crocea	GCF_009932435.1	3782613	Contig	6/01/2020	Yes
44	Gordonia crocea	GCA_009932435.1	3782613	Contig	6/01/2020	No

No.	Organism Name	Assembly Accession	Assembly Stats Total Sequence Length	Assembly Level	Assembly Submission Date	Reference Genome
45	Gordonia desulfuricans	GCF_010119475.1	5549881	Contig	3/02/2020	No
46	Gordonia desulfuricans	GCA_010119475.1	5549881	Contig	3/02/2020	No
47	Gordonia desulfuricans NBRC 100010	GCF_001485495.1	5428634	Contig	22/12/2015	Yes
48	Gordonia desulfuricans NBRC 100010	GCA_001485495.1	5428634	Contig	22/12/2015	No
49	Gordonia effusa NBRC 100432	GCF_000241305.1	4703019	Contig	2/12/2011	Yes
50	Gordonia effusa NBRC 100432	GCA_000241305.2	4703019	Contig	2/12/2011	No
51	Gordonia hankookensis	GCF_014673215.1	5221505	Contig	21/09/2020	Yes
52	Gordonia hankookensis	GCA_014673215.1	5221505	Contig	21/09/2020	No
53	Gordonia hirsuta DSM 44140 = NBRC 16056	GCF_000333015.1	3489460	Contig	11/01/2013	Yes
54	Gordonia hirsuta DSM 44140 = NBRC 16056	GCF_000420685.1	3489460	Contig	9/07/2013	No
55	Gordonia hirsuta DSM 44140 = NBRC 16056	GCA_000333015.1	3489460	Contig	11/01/2013	No
56	Gordonia hirsuta DSM 44140 = NBRC 16056	GCA_000420685.1	3489460	Contig	9/07/2013	No
57	Gordonia humi	GCF_014197435.1	5088437	Contig	14/08/2020	Yes
58	Gordonia humi	GCA_014197435.1	5088437	Contig	14/08/2020	No
59	Gordonia hydrophobica	GCF_016909615.1	4578665	Contig	19/02/2021	No
60	Gordonia hydrophobica	GCA_016909615.1	4578665	Contig	19/02/2021	No
61	Gordonia hydrophobica NBRC 16057	GCF_001592365.1	4579443	Contig	4/03/2016	Yes
62	Gordonia hydrophobica NBRC 16057	GCA_001592365.1	4579443	Contig	4/03/2016	No
63	Gordonia insulae	GCF_003855095.1	5962176	Complete Genome	2/12/2018	Yes
64	Gordonia insulae	GCA_003855095.1	5962176	Complete Genome	2/12/2018	No
65	Gordonia iterans	GCF_002993285.1	4006485	Complete Genome	11/03/2018	Yes
66	Gordonia iterans	GCA_002993285.1	4006485	Complete Genome	11/03/2018	No
67	Gordonia jacobaea	GCF_001186365.1	4925537	Contig	23/07/2015	Yes

No.	Organism Name	Assembly Accession	Assembly Stats Total Sequence Length	Assembly Level	Assembly Submission Date	Reference Genome
68	Gordonia jacobaea	GCA_001186365.1	4925537	Contig	23/07/2015	No
69	Gordonia jinghuaiqii	GCF_014041935.1	5184113	Complete Genome	2/08/2020	Yes
70	Gordonia jinghuaiqii	GCF_014622905.1	5199293	Contig	17/09/2020	No
71	Gordonia jinghuaiqii	GCA_014041935.1	5184113	Complete Genome	2/08/2020	No
72	Gordonia jinghuaiqii	GCA_014622905.1	5199293	Contig	17/09/2020	No
73	Gordonia jinhuaensis	GCF_014639795.1	4447633	Contig	12/09/2020	Yes
74	Gordonia jinhuaensis	GCA_014639795.1	4447633	Contig	12/09/2020	No
75	Gordonia lacunae	GCF_002149015.1	5756417	Contig	18/05/2017	Yes
76	Gordonia lacunae	GCA_002149015.1	5756417	Contig	18/05/2017	No
77	Gordonia malaquae	GCF_900105435.1	4714353	Contig	22/10/2016	No
78	Gordonia malaquae	GCA_900105435.1	4714353	Contig	22/10/2016	No
79	Gordonia malaquae NBRC 108250	GCF_000344135.1	4465464	Contig	27/02/2013	Yes
80	Gordonia malaquae NBRC 108250	GCA_000344135.1	4465464	Contig	27/02/2013	No
81	Gordonia namibiensis NBRC 108229	GCF_000298235.1	4941607	Contig	7/09/2012	Yes
82	Gordonia namibiensis NBRC 108229	GCA_000298235.1	4941607	Contig	7/09/2012	No
83	Gordonia neofelifaecis NRRL B-59395	GCF_000192435.1	4257286	Contig	15/03/2011	Yes
84	Gordonia neofelifaecis NRRL B-59395	GCA_000192435.2	4257286	Contig	15/03/2011	No
85	Gordonia oryzae	GCF_003797185.1	4519536	Contig	19/11/2018	Yes
86	Gordonia oryzae	GCA_003797185.1	4519536	Contig	19/11/2018	No
87	Gordonia otitidis	GCF_020735545.1	5108829	Complete Genome	3/11/2021	No
88	Gordonia otitidis	GCA_020735545.1	5108829	Complete Genome	3/11/2021	No
89	Gordonia otitidis NBRC 100426	GCF_000248075.1	5295895	Contig	18/02/2012	Yes
90	Gordonia otitidis NBRC 100426	GCA_000248075.2	5295895	Contig	18/02/2012	No

No.	Organism Name	Assembly Accession	Assembly Stats Total Sequence Length	Assembly Level	Assembly Submission Date	Reference Genome
91	Gordonia paraffinivorans	GCF_900683595.1	4511003	Contig	21/02/2019	Yes
92	Gordonia paraffinivorans	GCF_003121315.1	4744413	Contig	11/05/2018	No
93	Gordonia paraffinivorans	GCF_012494185.1	4368946	Contig	17/04/2020	No
94	Gordonia paraffinivorans	GCF_012494195.1	4336980	Contig	17/04/2020	No
95	Gordonia paraffinivorans	GCF_012498505.1	4314372	Contig	17/04/2020	No
96	Gordonia paraffinivorans	GCF_012498525.1	4447680	Contig	17/04/2020	No
97	Gordonia paraffinivorans	GCF_012498535.1	4333866	Contig	17/04/2020	No
98	Gordonia paraffinivorans	GCF_012498545.1	4263553	Contig	17/04/2020	No
99	Gordonia paraffinivorans	GCF_012498585.1	4316110	Contig	17/04/2020	No
100	Gordonia paraffinivorans	GCF_012498595.1	4313952	Contig	17/04/2020	No
101	Gordonia paraffinivorans	GCF_012498665.1	4467653	Contig	17/04/2020	No
102	Gordonia paraffinivorans	GCF_019788915.1	4889674	Contig	27/08/2021	No
103	Gordonia paraffinivorans	GCF_021026155.1	4452968	Scaffold	1/12/2021	No
104	Gordonia paraffinivorans	GCA_003121315.1	4744413	Contig	11/05/2018	No
105	Gordonia paraffinivorans	GCA_012494185.1	4368946	Contig	17/04/2020	No
106	Gordonia paraffinivorans	GCA_012494195.1	4336980	Contig	17/04/2020	No
107	Gordonia paraffinivorans	GCA_012498505.1	4314372	Contig	17/04/2020	No
108	Gordonia paraffinivorans	GCA_012498525.1	4447680	Contig	17/04/2020	No
109	Gordonia paraffinivorans	GCA_012498535.1	4333866	Contig	17/04/2020	No
110	Gordonia paraffinivorans	GCA_012498545.1	4263553	Contig	17/04/2020	No
111	Gordonia paraffinivorans	GCA_012498585.1	4316110	Contig	17/04/2020	No
112	Gordonia paraffinivorans	GCA_012498595.1	4313952	Contig	17/04/2020	No
113	Gordonia paraffinivorans	GCA_012498665.1	4467653	Contig	17/04/2020	No

No.	Organism Name	Assembly Accession	Assembly Stats Total Sequence Length	Assembly Level	Assembly Submission Date	Reference Genome
114	Gordonia paraffinivorans	GCA_019788915.1	4889674	Contig	27/08/2021	No
115	Gordonia paraffinivorans	GCA_021026155.1	4452968	Scaffold	1/12/2021	No
116	Gordonia paraffinivorans	GCA_900683595.1	4511003	Contig	21/02/2019	No
117	Gordonia paraffinivorans NBRC 108238	GCF_000344155.1	4632332	Contig	27/02/2013	No
118	Gordonia paraffinivorans NBRC 108238	GCA_000344155.1	4632332	Contig	27/02/2013	No
119	Gordonia phthalatica	GCF_001305675.1	4428727	Complete Genome	2/10/2015	Yes
120	Gordonia phthalatica	GCA_001305675.1	4428727	Complete Genome	2/10/2015	No
121	Gordonia polyisoprenivorans	GCF_002257745.1	6180512	Contig	17/08/2017	No
122	Gordonia polyisoprenivorans	GCF_012396285.1	6287369	Contig	15/04/2020	No
123	Gordonia polyisoprenivorans	GCF_017654315.1	6033879	Complete Genome	31/03/2021	No
124	Gordonia polyisoprenivorans	GCF_018135605.1	5925347	Complete Genome	23/04/2021	No
125	Gordonia polyisoprenivorans	GCA_002257745.1	6180512	Contig	17/08/2017	No
126	Gordonia polyisoprenivorans	GCA_012396285.1	6287369	Contig	15/04/2020	No
127	Gordonia polyisoprenivorans	GCA_017654315.1	6033879	Complete Genome	31/03/2021	No
128	Gordonia polyisoprenivorans	GCA_018135605.1	5925347	Complete Genome	23/04/2021	No
129	Gordonia polyisoprenivorans HW436	GCF_000385355.1	6328722	Contig	26/04/2013	No
130	Gordonia polyisoprenivorans HW436	GCA_000385355.1	6328722	Contig	26/04/2013	No
131	Gordonia polyisoprenivorans NBRC 16320 = JCM 10675	GCF_000241325.1	6285478	Contig	2/12/2011	No
132	Gordonia polyisoprenivorans NBRC 16320 = JCM 10675	GCA_000241325.2	6285478	Contig	2/12/2011	No
133	Gordonia polyisoprenivorans VH2	GCF_000247715.1	5844299	Complete Genome	16/02/2012	Yes
134	Gordonia polyisoprenivorans VH2	GCA_000247715.1	5844299	Complete Genome	16/02/2012	No

No.	Organism Name	Assembly Accession	Assembly Stats Total Sequence Length	Assembly Level	Assembly Submission Date	Reference Genome
135	Gordonia pseudoamarae	GCF_009914535.1	5306277	Complete Genome	22/01/2020	Yes
136	Gordonia pseudoamarae	GCA_009914535.1	5306277	Complete Genome	22/01/2020	No
137	Gordonia rhizosphera NBRC 16068	GCF_000298195.1	6434991	Contig	5/09/2012	Yes
138	Gordonia rhizosphera NBRC 16068	GCA_000298195.1	6434991	Contig	5/09/2012	No
139	Gordonia rubripertincta	GCF_014070455.1	5263177	Complete Genome	5/08/2020	Yes
140	Gordonia rubripertincta	GCF_003568625.1	5332073	Complete Genome	2/08/2017	No
141	Gordonia rubripertincta	GCF_007489265.1	4857317	Contig	25/07/2019	No
142	Gordonia rubripertincta	GCF_012396225.1	5699719	Contig	15/04/2020	No
143	Gordonia rubripertincta	GCF_016905985.1	5155927	Scaffold	17/02/2021	No
144	Gordonia rubripertincta	GCA_003568625.1	5332073	Complete Genome	2/08/2017	No
145	Gordonia rubripertincta	GCA_007489265.1	4857317	Contig	25/07/2019	No
146	Gordonia rubripertincta	GCA_012396225.1	5699719	Contig	15/04/2020	No
147	Gordonia rubripertincta	GCA_014070455.1	5263177	Complete Genome	5/08/2020	No
148	Gordonia rubripertincta	GCA_016905985.1	5155927	Scaffold	17/02/2021	No
149	Gordonia rubripertincta NBRC 101908	GCF_000327325.1	5204225	Contig	5/09/2012	No
150	Gordonia rubripertincta NBRC 101908	GCA_000327325.1	5204225	Contig	5/09/2012	No
151	Gordonia shandongensis DSM 45094	GCF_000423025.1	3330647	Scaffold	11/07/2013	Yes
152	Gordonia shandongensis DSM 45094	GCA_000423025.1	3330647	Scaffold	11/07/2013	No
153	Gordonia sihwensis	GCF_000960465.1	4155606	Contig	23/03/2015	No
154	Gordonia sihwensis	GCF_019788935.1	3979219	Contig	27/08/2021	No
155	Gordonia sihwensis	GCA_000960465.1	4155606	Contig	23/03/2015	No
156	Gordonia sihwensis	GCA_019788935.1	3979219	Contig	27/08/2021	No
157	Gordonia sihwensis NBRC 108236	GCF_000333035.1	4140890	Contig	11/01/2013	Yes

No.	Organism Name	Assembly Accession	Assembly Stats Total Sequence Length	Assembly Level	Assembly Submission Date	Reference Genome
158	Gordonia sihwensis NBRC 108236	GCA_000333035.1	4140890	Contig	11/01/2013	No
159	Gordonia soli NBRC 108243	GCF_000334455.1	5375815	Contig	25/01/2013	Yes
160	Gordonia soli NBRC 108243	GCA_000334455.1	5375815	Contig	25/01/2013	No
161	Gordonia sp. 135	GCF_009720185.1	5204790	Complete Genome	28/11/2019	No
162	Gordonia sp. 135	GCA_009720185.1	5204790	Complete Genome	28/11/2019	No
163	Gordonia sp. 1D	GCF_002327125.1	5151623	Chromosome	21/09/2017	No
164	Gordonia sp. 1D	GCA_002327125.1	5151623	Chromosome	21/09/2017	No
165	Gordonia sp. 852002-10350_SCH5691597	GCF_001665715.1	5109980	Contig	17/06/2016	No
166	Gordonia sp. 852002-10350_SCH5691597	GCA_001665715.1	5109980	Contig	17/06/2016	No
167	Gordonia sp. 852002-50395_SCH5434458	GCF_001665905.1	5001909	Contig	17/06/2016	No
168	Gordonia sp. 852002-50395_SCH5434458	GCA_001665905.1	5001909	Contig	17/06/2016	No
169	Gordonia sp. 852002-50816_SCH5313054-a	GCF_001665745.1	5135204	Contig	17/06/2016	No
170	Gordonia sp. 852002-50816_SCH5313054-a	GCA_001665745.1	5135204	Contig	17/06/2016	No
171	Gordonia sp. 852002-50816_SCH5313054-c	GCF_001665925.1	5114218	Contig	17/06/2016	No
172	Gordonia sp. 852002-50816_SCH5313054-c	GCA_001665925.1	5114218	Contig	17/06/2016	No
173	Gordonia sp. 852002-51296_SCH5728562-b	GCF_001665475.1	5140530	Contig	17/06/2016	No
174	Gordonia sp. 852002-51296_SCH5728562-b	GCA_001665475.1	5140530	Contig	17/06/2016	No
175	Gordonia sp. ALPHA1B1	GCF_005502615.1	5204864	Contig	17/05/2019	No
176	Gordonia sp. ALPHA1B1	GCA_005502615.1	5204864	Contig	17/05/2019	No
177	Gordonia sp. ALPHA2A	GCF_005502605.1	5192981	Contig	17/05/2019	No
178	Gordonia sp. ALPHA2A	GCA_005502605.1	5192981	Contig	17/05/2019	No
179	Gordonia sp. BP-119	GCF_016924115.1	5278186	Contig	23/02/2021	No
180	Gordonia sp. BP-119	GCA_016924115.1	5278186	Contig	23/02/2021	No

No.	Organism Name	Assembly Accession	Assembly Stats Total Sequence Length	Assembly Level	Assembly Submission Date	Reference Genome
181	Gordonia sp. BP-94	GCF_016924075.1	5278873	Contig	23/02/2021	No
182	Gordonia sp. BP-94	GCA_016924075.1	5278873	Contig	23/02/2021	No
183	Gordonia sp. CNJ-863	GCF_001942325.1	5398721	Scaffold	11/01/2017	No
184	Gordonia sp. CNJ-863	GCA_001942325.1	5398721	Scaffold	11/01/2017	No
185	Gordonia sp. GAMMA	GCF_005502535.1	5343593	Contig	17/05/2019	No
186	Gordonia sp. GAMMA	GCA_005502535.1	5343593	Contig	17/05/2019	No
187	Gordonia sp. HNM0687	GCF_009828195.1	5365300	Scaffold	2/01/2020	No
188	Gordonia sp. HNM0687	GCA_009828195.1	5365300	Scaffold	2/01/2020	No
189	Gordonia sp. HS-NH1	GCF_001418765.1	5429264	Scaffold	28/10/2015	No
190	Gordonia sp. HS-NH1	GCA_001418765.1	5429264	Scaffold	28/10/2015	No
191	Gordonia sp. IITR100	GCF_002009645.1	5285492	Scaffold	2/03/2017	No
192	Gordonia sp. IITR100	GCA_002009645.1	5285492	Scaffold	2/03/2017	No
193	Gordonia sp. JH63	GCF_009856645.1	5338743	Complete Genome	11/01/2020	No
194	Gordonia sp. JH63	GCA_009856645.1	5338743	Complete Genome	11/01/2020	No
195	Gordonia sp. KTR9	GCF_000143885.2	5885710	Complete Genome	10/09/2012	No
196	Gordonia sp. KTR9	GCA_000143885.2	5885710	Complete Genome	10/09/2012	No
197	Gordonia sp. LAM0048	GCF_001659625.1	5654747	Contig	7/06/2016	No
198	Gordonia sp. LAM0048	GCA_001659625.1	5654747	Contig	7/06/2016	No
199	Gordonia sp. NB4-1Y	GCF_000347295.2	5626047	Contig	10/09/2015	No
200	Gordonia sp. NB4-1Y	GCA_000347295.2	5626047	Contig	10/09/2015	No
201	Gordonia sp. OPL2	GCF_003797825.1	5795898	Contig	19/11/2018	No
202	Gordonia sp. OPL2	GCA_003797825.1	5795898	Contig	19/11/2018	No
203	Gordonia sp. PDNC005	GCF_016919385.1	4260791	Complete Genome	23/02/2021	No

No.	Organism Name	Assembly Accession	Assembly Stats Total Sequence Length	Assembly Level	Assembly Submission Date	Reference Genome
204	Gordonia sp. PDNC005	GCA_016919385.1	4260791	Complete Genome	23/02/2021	No
205	Gordonia sp. QH-12	GCF_001578675.1	3901461	Scaffold	2/03/2016	No
206	Gordonia sp. QH-12	GCA_001578675.1	3901461	Scaffold	2/03/2016	No
207	Gordonia sp. SCSIO 19800	GCF_018132775.1	5459426	Scaffold	22/04/2021	No
208	Gordonia sp. SCSIO 19800	GCA_018132775.1	5459426	Scaffold	22/04/2021	No
209	Gordonia sp. SGD-V-85	GCF_001456905.1	5439079	Contig	4/12/2015	No
210	Gordonia sp. SGD-V-85	GCA_001456905.1	5439079	Contig	4/12/2015	No
211	Gordonia sp. SID5947	GCF_009862785.1	5099185	Contig	13/01/2020	No
212	Gordonia sp. SID5947	GCA_009862785.1	5099185	Contig	13/01/2020	No
213	Gordonia sp. UCD-TK1	GCF_001691435.1	5464889	Contig	21/07/2016	No
214	Gordonia sp. UCD-TK1	GCA_001691435.1	5464889	Contig	21/07/2016	No
215	Gordonia sp. WA4-43	GCF_020520305.1	5438735	Complete Genome	18/10/2021	No
216	Gordonia sp. WA4-43	GCA_020520305.1	5438735	Complete Genome	18/10/2021	No
217	Gordonia sp. YC-JH1	GCF_002848445.1	4193324	Complete Genome	28/12/2017	No
218	Gordonia sp. YC-JH1	GCA_002848445.1	4193324	Complete Genome	28/12/2017	No
219	Gordonia sp. YY1	GCF_009872315.1	5132514	Scaffold	14/01/2020	No
220	Gordonia sp. YY1	GCA_009872315.1	5132514	Scaffold	14/01/2020	No
221	Gordonia sp. i37	GCF_002043085.1	6228158	Contig	20/03/2017	No
222	Gordonia sp. i37	GCA_002043085.1	6228158	Contig	20/03/2017	No
223	Gordonia sp. v-85	GCF_900094715.1	5439079	Contig	4/08/2016	No
224	Gordonia sp. v-85	GCA_900094715.1	5439079	Contig	4/08/2016	No
225	Gordonia spumicola	GCF_009932475.1	4886396	Contig	6/01/2020	Yes
226	Gordonia spumicola	GCA_009932475.1	4886396	Contig	6/01/2020	No

No.	Organism Name	Assembly Accession	Assembly Stats Total Sequence Length	Assembly Level	Assembly Submission Date	Reference Genome
227	Gordonia sputi	GCF_012396255.1	4961688	Contig	15/04/2020	No
228	Gordonia sputi	GCA_012396255.1	4961688	Contig	15/04/2020	No
229	Gordonia sputi NBRC 100414	GCF_000248055.1	4952979	Contig	18/02/2012	Yes
230	Gordonia sputi NBRC 100414	GCA_000248055.2	4952979	Contig	18/02/2012	No
231	Gordonia terrae	GCF_901542405.1	5706840	Contig	2/06/2019	Yes
232	Gordonia terrae	GCF_000716975.1	5673297	Contig	1/07/2014	No
233	Gordonia terrae	GCF_001698225.1	5701501	Complete Genome	2/08/2016	No
234	Gordonia terrae	GCF_002847865.1	5703010	Scaffold	28/12/2017	No
235	Gordonia terrae	GCF_003183825.1	5708950	Complete Genome	3/06/2018	No
236	Gordonia terrae	GCF_005502725.1	5190646	Contig	17/05/2019	No
237	Gordonia terrae	GCF_011290605.1	5312854	Complete Genome	15/03/2020	No
238	Gordonia terrae	GCA_000716975.1	5673297	Contig	1/07/2014	No
239	Gordonia terrae	GCA_001698225.1	5701501	Complete Genome	2/08/2016	No
240	Gordonia terrae	GCA_002847865.1	5703010	Scaffold	28/12/2017	No
241	Gordonia terrae	GCA_003183825.1	5708950	Complete Genome	3/06/2018	No
242	Gordonia terrae	GCA_005502725.1	5190646	Contig	17/05/2019	No
243	Gordonia terrae	GCA_011290605.1	5312854	Complete Genome	15/03/2020	No
244	Gordonia terrae	GCA_901542405.1	5706840	Contig	2/06/2019	No
245	Gordonia terrae C-6	GCF_000390025.1	5170241	Contig	8/05/2013	No
246	Gordonia terrae C-6	GCA_000390025.1	5170241	Contig	8/05/2013	No
247	Gordonia terrae NBRC 100016	GCF_000248035.1	5669149	Contig	18/02/2012	No
248	Gordonia terrae NBRC 100016	GCA_000248035.2	5669149	Contig	18/02/2012	No
249	Gordonia westfalica	GCF_900105725.1	6405003	Contig	22/10/2016	Yes

No.	Organism Name	Assembly Accession	Assembly Stats Total Sequence Length	Assembly Level	Assembly Submission Date	Reference Genome
250	Gordonia westfalica	GCA_900105725.1	6405003	Contig	22/10/2016	No
251	Gordonia zhaorongruii	GCF_007559005.1	3233079	Complete Genome	28/07/2019	Yes
252	Gordonia zhaorongruii	GCF_004353055.1	3277954	Contig	15/03/2019	No
253	Gordonia zhaorongruii	GCA_004353055.1	3277954	Contig	15/03/2019	No
254	Gordonia zhaorongruii	GCA_007559005.1	3233079	Complete Genome	28/07/2019	No

Table S4. Data on *Gordonia*-derived compounds retrieved from the articles included in the systematic review.

ID	Type	Name	Class	SMILES
**1**	Annoated	Cladoniamide C	Alkoloid	CN1C(=O)[C@@]2(C3=C(C4=C(C5=CC=CC=C5N4[C@@]2(C1=O)O)OC)NC6=CC=CC=C63)O
**2**	Annoated	Pimprinine	Alkoloid	CC1=NC=C(O1)C2=CNC3=CC=CC=C32
**3**	Isolated	soraphinol A	Alkaloid	OC(CC1=CNC2=C1C=CC=C2)C(=O)CC1=CC=C(O)C=C1
**4**	Isolated	kurasoin B	Alkaloid	OC(CC1=CNC2=C1C=CC=C2)C(=O)CC1=CC=CC=C1
**5**	Isolated	diolmycin A2	Alkaloid	O[C@@H](CC1=CNC2=C1C=CC=C2)[C@@H](O)CC1=CC=C(O)C=C1
**6**	Isolated	diolmycin A1	Alkaloid	O[C@@H](CC1=CNC2=C1C=CC=C2)[C@H](O)CC1=CC=C(O)C=C1
**7**	Isolated	Deferrioxamine E	Amide	C1CCNC(=O)CCC(=O)N(CCCCCNC(=O)CCC(=O)N(CCCCCNC(=O)CCC(=O)N(CC1)O)O)O
**8**	Isolated	Mojavensin A	Amide	CCC(C)CCCCCCCCC1CC(=O)N[C@@H](C(=O)N[C@@H](C(=O)N[C@H](C(=O)N[C@H](C(=O)N2CCC[C@H]2C(=O)N[C@H](C(=O)N[C@H](C(=O)N1)CC(=O)N)CC(=O)N)CCC(=O)N)CC(=O)N)CC3=CC=C(C=C3)O)CC(=O)N
**9**	Isolated	cyclo(Leu-Leu)	Amide	CC(C)CC1NC(=O)C(CC(C)C)NC1=O
**10**	Isolated	Actinomycin D	Amide	C[C@@H]1[C@@H](C(=O)N[C@@H](C(=O)N2CCC[C@H]2C(=O)N(CC(=O)N([C@H](C(=O)O1)C(C)C)C)C)C(C)C)NC(=O)C3=C4C(=C(C=C3)C)OC5=C(C(=O)C(=C(C5=N4)C(=O)

ID	Type	Name	Class	SMILES
				N[C@H]6[C@H](OC(=O)[C@@H](N(C(=O)CN(C(=O)[C@@H]7CCCN7C(=O)[C@H](NC6=O)C(C)C)C)C)C(C)C)N)C
**11**	Isolated	Actinomycin X2	Amide	CC1C(C(=O)NC(C(=O)N2CCCC2C(=O)N(CC(=O)N(C(C(=O)O1)C(C)C)C)C)C(C)C)NC(=O)C3=C4C(=C(C=C3)C)OC5=C(C(=O)C(=C(C5=N4)C(=O)NC6C(OC(=O)C(N(C(=O)CN(C(=O)C7CC(=O)CN7C(=O)C(NC6=O)C(C)C)C)C)C(C)C)N)C
**12**	Annoated	Fujianmycin A	Phenylpropanoid	C[C@H]1CC(=O)C2=C([C@@H]1O)C=CC3=C2C(=O)C4=C(C3=O)C(=CC=C4)O
**13**	Annoated	Atramycin A	Phenylpropanoid	C[C@H]1CC2=CC(=C3C(=C2C(=O)C1)C(=O)C4=C(C3=O)C(=CC=C4)O[C@H]5[C@@H]([C@@H]([C@H]([C@@H](O5)C)O)O)O)O
**14**	Annoated	Atramycin B	Phenylpropanoid	C[C@H]1CC2=C(C(=O)C1)C3=C(C=C2)C(=O)C4=C(C3=O)C=CC=C4O[C@H]5[C@@H]([C@@H]([C@H]([C@@H](O5)C)O)O)O
**15**	Isolated	Circumcin A	Phenylpropanoid	O[C@@H](CC1=CC=CC=C1)[C@@H](O)CC1=CC=C(O)C=C1
**16**	Isolated	Circumcin B	Phenylpropanoid	OC(CC1=CC=C(O)C=C1)C(=O)CC1=CC=C(O)C=C1
**17**	Isolated	Circumcin C	Phenylpropanoid	OC(CC1=CC=CC=C1)C(=O)CC1=CC=CC=C1
**18**	Isolated	kurasoin A	Phenylpropanoid	OC(CC1=CC=C(O)C=C1)C(=O)CC1=CC=CC=C1
**19**	Isolated	1,4-diphenyl-2,3-butanediol	Phenylpropanoid	O[C@@H](CC1=CC=CC=C1)[C@@H](O)CC1=CC=CC=C1
**20**	Isolated	diolmycin B2	Phenylpropanoid	O[C@@H](CC1=CC=C(O)C=C1)[C@@H](O)CC1=CC=C(O)C=C1
**21**	Isolated	4-hydroxysattabacin	Terpenoid	CC(C)CC(=O)C(O)CC1=CC=C(O)C=C1
**22**	Isolated	soraphinol C	Terpenoid	CC(C)CC(O)C(=O)CC1=CC=C(O)C=C1
**23**	Annoated	Canthaxanthin	Terpenoid	CC1=C(C(CCC1=O)(C)C)/C=C/C(=C/C=C/C(=C/C=C/C=C(/C=C/C=C(/C=C/C2=C(C(=O)CCC2(C)C)C)\C)\C)/C)/C
**24**	Isolated	Ketodeoxymyxol glucoside	Terpenoid	CC1=C(C(CCC1=O)(C)C)/C=C/C(=C/C=C/C(=C/C=C/C=C(\C)/C=C/C=C(\C)/C=C/C=C(\C)/C=C/[C@@H](C(C)(C)O[C@H]2[C@@H]([C@H]([C@@H]([C@H](O2)CO)O)O)O)O)/C)/C

ID	Type	Name	Class	SMILES
**25**	Isolated	4-Keto-gamma-carotene	Terpenoid	CC1=C(C(CCC1=O)(C)C)/C=C/C(=C/C=C/C(=C/C=C/C=C(\C)/C=C/C=C(\C)/C=C/C=C(\C)/CCC=C(C)C)/C)/C
**26**	Isolated	Gordonic acid	Terpenoid	C[C@@H]1[C@H]([C@H](C[C@@H](O1)O[C@H]([C@H](C)/C=C(\C)/C=C/C=C/C=C/C=C/C(=O)O)C(C)C)O)O
**27**	Isolated	Deoxymyxol glucoside	Terpenoid	CC1=C(C(CCC1)(C)C)/C=C/C(=C/C=C/C(=C/C=C/C=C(\C)/C=C/C=C(\C)/C=C/C=C(\C)/C=C/[C@@H](C(C)(C)O[C@H]2C(C([C@@H]([C@H](O2)CO)O)O)O)O)/C)/C
**28**	Isolated	Bendigol A	Terpenoid	C[C@@H]([C@H]1CC[C@@H]2[C@@]1([C@H](C[C@H]3[C@H]2CCC4=CC(=O)C=C[C@]34C)O)C)C(=O)O
**29**	Isolated	Bendigol B	Terpenoid	C[C@@H]([C@H]1CC[C@@H]2[C@@]1([C@H](C[C@H]3[C@H]2CCC4=CC(=O)C=C[C@]34C)O)C)C(=O)C
**30**	Isolated	Bendigol C	Terpenoid	C[C@@H]([C@H]1CC[C@@H]2[C@@]1([C@H](C[C@H]3[C@H]2CCC4=CC(=O)C=C[C@]34C)O)C)C(CC(=O)O)O

Table S5. Clustering of Gordonia-derived compounds by similarity to Streptomyces-derived compounds..

Genus	Comp ID	Name	Neighbor Similarity FragFp 85%	Neighbor Count	Neighbor	Neighbor Analysis X	Neighbor Analysis Y
Gordonia	G1	Cladoniamide C	1 (max of 14)	14	S1201; S9103; S9529; S10248; S10249; S10252; S10475; S11343; S11344; S11345; S11346; S11573; S11924; S11980	-0.5573528	0.7212385
Gordonia	G10	Actinomycin D	1 (max of 24)	24	G11; S784; S4774; S5020; S8829; S9167; S9492; S10713; S10830; S11253; S12312; S12313; S12314; S12315; S12484; S12704; S12705; S12720; S12816; S13167; S13168; S13169; S13170; S13171	-0.6881992	-0.2810932
Gordonia	G11	Actinomycin X2	1 (max of 24)	24	S784; S4774; S5020; S8829; S9167; S9492; S10713; S10830; S11253; S12312; S12313; S12314; S12315; S12484; S12704; S12705; S12720; S12816; S13167; S13168; S13169; S13170; S13171; G10	-0.4081382	-0.20047481

Genus	Comp ID	Name	Neighbor Similarity FragFp 85%	Neighbor Count	Neighbor	Neighbor Analysis X	Neighbor Analysis Y
Gordonia	G12	Fujianmycin A	1 (max of 4)	4	S2987; S2988; S11316; S11317	0.29076543	-0.7472032
Gordonia	G13	Atramycin A	1 (max of 9)	9	G14; S3045; S3046; S4497; S10808; S11752; S12637; S13094; S13652	0.86065096	-0.27398786
Gordonia	G14	Atramycin B	1 (max of 9)	9	S3045; S3046; S4497; S10808; S11752; S12637; S13094; S13652; G13	0.66279924	-0.26118183
Gordonia	G15	Circumcin A	1 (max of 3)	3	G20; G19; S3837	0.8799941	0.16236462
Gordonia	G19	1,4-diphenyl-2,3-butanediol	0.8625 (max of 4)	4	G20; S3837; S11248; G15	0.7996641	0.28032556
Gordonia	G2	Pimprinine	1 (max of 2)	2	S2944; S4820	0.26714557	0.007862226
Gordonia	G20	diolmycin B2	1 (max of 3)	3	S3837; G15; G19	0.6856618	0.21190915
Gordonia	G26	Gordonic acid	0.91534 (max of 5)	5	S12583; S12584; S12585; S12586; S12587	0.65447026	0.72304666
Gordonia	G29	Bendigol B	0.875 (max of 2)	2	S5050; S10587	0.6290969	-0.75525
Gordonia	G5	diolmycin A2	1 (max of 2)	2	S3838; G6	-0.22656251	-0.8347393
Gordonia	G6	diolmycin A1	1 (max of 2)	2	G5; S3838	-0.3419507	-0.73550284
Gordonia	G7	Deferrioxamine E	1 (max of 15)	15	S3; S4747; S9861; S10124; S10158; S11442; S11443; S11444; S11729; S11841; S11873; S12329; S12332; S12337; S13750	-0.45083806	0.41100547
Gordonia	G8	Mojavensin A	0.97 (max of 11)	11	S2960; S5079; S9880; S11139; S11140; S11424; S12173; S12393; S12435; S12676; S13629	0.39515775	0.30940273
Gordonia	G9	cyclo(Leu-Leu)	0.99457 (max of 11)	11	S3704; S4070; S4535; S4896; S9704; S9853; S10155; S11693; S11821; S12067; S13104	0.29447567	-0.42450133
Streptomyces	S10124	Dehydroxynocardamine	1 (max of 15)	15	G7; S13750; S12337; S12332; S12329; S11873; S11841; S11729; S11444; S11443; S11442; S10158; S3; S4747; S9861	0.0764843	0.24095628
Streptomyces	S10155	CHEMBL1957401	1 (max of 11)	11	G9; S13104; S12067; S11821; S11693; S3704; S4070; S4535; S4896; S9704; S9853	0.37356967	-0.28679296

Genus	Comp ID	Name	Neighbor Similarity FragFp 85%	Neighbor Count	Neighbor	Neighbor Analysis X	Neighbor Analysis Y
Streptomyces	S10158	Desmethylenylnocardamine	0.97778 (max of 14)	14	G7; S13750; S12337; S12332; S11873; S11841; S11729; S11444; S11443; S11442; S3; S4747; S9861; S10124	-0.21470055	0.20092246
Streptomyces	S10248	CHEBI:84366	0.99284 (max of 6)	6	G1; S10475; S10252; S10249; S9103; S9529	-0.3478083	0.69048804
Streptomyces	S10249	CHEBI:84368	1 (max of 14)	14	G1; S11980; S11924; S11573; S11346; S11345; S11344; S11343; S10475; S10252; S1201; S9103; S9529; S10248	-0.63790256	0.6154629
Streptomyces	S10252	CHEBI:84367	0.99284 (max of 6)	6	G1; S10475; S9103; S9529; S10248; S10249	-0.56106365	0.5176044
Streptomyces	S10475	UNII-T7972EPW8P	0.99043 (max of 6)	6	G1; S9103; S9529; S10248; S10249; S10252	-0.4184628	0.5633561
Streptomyces	S10587	(1R,3aS,3bS,9aR,11aS)-1-[(1S)-1,2-Dihydroxyethyl]-1,10-dihydroxy-9a,11a-dimethyl-1H,2H,3H,3aH,3bH,4H,5H,7H,9aH,9bH,10H,11H,11aH-cyclopenta[a]phenanthren-7-one	0.85027	1	G29	0.5298342	-0.8002052
Streptomyces	S10713	Actinomycin D	1 (max of 24)	24	G11; G10; S13171; S13170; S13169; S13168; S13167; S12816; S12720; S12705; S12704; S12484; S12315; S12314; S12313; S12312; S11253; S10830; S784; S4774; S5020; S8829; S9167; S9492	-0.55526215	-0.23737225
Streptomyces	S10808	Brasiliquinone A	0.86447 (max of 4)	4	G14; G13; S3045; S3046	0.7531602	-0.36845252
Streptomyces	S10830	Actinomycin V	1 (max of 24)	24	G11; G10; S13171; S13170; S13169; S13168; S13167; S12816; S12720; S12705; S12704;	-0.639764	0.021827398

Genus	Comp ID	Name	Neighbor Similarity FragFp 85%	Neighbor Count	Neighbor	Neighbor Analysis X	Neighbor Analysis Y
					S12484; S12315; S12314; S12313; S12312; S11253; S784; S4774; S5020; S8829; S9167; S9492; S10713		
Streptomyces	S11126	Daidzein-7-alpha-L-rhamnoside	1 (max of 9)	9	S13094; S11974; S11752; S11746; S11745; S11744; S11127; S4497; S4500	0.5184798	-0.5397491
Streptomyces	S11127	Genistein-7-alpha-L-rhamnoside	1 (max of 9)	9	S13094; S11974; S11752; S11746; S11745; S11744; S4497; S4500; S11126	0.61818993	-0.71331525
Streptomyces	S11139	Provipeptide A	0.93929 (max of 10)	10	G8; S13629; S12676; S12435; S12173; S11424; S11140; S2960; S5079; S9880	0.55640054	0.33945614
Streptomyces	S11140	Provipeptide B	0.93929 (max of 4)	4	G8; S12173; S5079; S11139	0.29624796	0.44067225
Streptomyces	S11248	(2S,3S)-4-Methyl-1-phenylpentane-2,3-diol	0.85185	1	G19	0.8393033	0.37607795
Streptomyces	S11253	Actinomycin X0(beta)	0.99274 (max of 23)	23	G11; G10; S13171; S13170; S13169; S13168; S13167; S12816; S12720; S12704; S12484; S12315; S12314; S12313; S12312; S784; S4774; S5020; S8829; S9167; S9492; S10713; S10830	-0.48958367	0.06832806
Streptomyces	S11316	Donghaesulfin A	1 (max of 4)	4	G12; S11317; S2987; S2988	0.20317037	-0.84963506
Streptomyces	S11317	Donghaesulfin B	1 (max of 4)	4	G12; S2987; S2988; S11316	0.050695956	-0.8590875
Streptomyces	S11343	Cyclomarin B	0.98744 (max of 10)	10	G1; S11980; S11573; S11346; S11345; S11344; S1201; S9103; S9529; S10249	-0.66693616	0.6846386
Streptomyces	S11344	Cyclomarin C	1 (max of 7)	7	G1; S11573; S11346; S11345; S1201; S10249; S11343	-0.74159074	0.5774962
Streptomyces	S11345	Cyclomarin D	1 (max of 7)	7	G1; S11573; S11346; S1201; S10249; S11343; S11344	-0.7561983	0.63391507
Streptomyces	S11346	Desoxyclomarin C	0.99217 (max of 7)	7	G1; S11980; S1201; S10249; S11343; S11344; S11345	-0.67449826	0.7254344

Genus	Comp ID	Name	Neighbor Similarity FragFp 85%	Neighbor Count	Neighbor	Neighbor Analysis X	Neighbor Analysis Y
Streptomyces	S11424	Carmabin A	0.87255 (max of 4)	4	G8; S12173; S5079; S11139	0.6950376	0.37043694
Streptomyces	S11442	Ferrioxamine D1	0.99248 (max of 14)	14	G7; S13750; S12337; S12332; S11873; S11841; S11729; S11444; S11443; S3; S4747; S9861; S10124; S10158	-0.32466248	0.105525546
Streptomyces	S11443	Ferrioxamine B	1 (max of 15)	15	G7; S13751; S13750; S12337; S12332; S11873; S11841; S11729; S11444; S3; S4747; S9861; S10124; S10158; S11442	-0.45468163	0.22948441
Streptomyces	S11444	Ferrioxamine B+CH2	1 (max of 15)	15	G7; S13751; S13750; S12337; S12332; S11873; S11841; S11729; S3; S4747; S9861; S10124; S10158; S11442; S11443	-0.32959467	0.3250432
Streptomyces	S11573	IDM F	0.88642 (max of 6)	6	G1; S11924; S10249; S11343; S11344; S11345	-0.804556	0.45395276
Streptomyces	S11693	cyclo(Val-Leu)	1 (max of 4)	4	G9; S4070; S9853; S10155	0.29814982	-0.14187758
Streptomyces	S11729	Proferrioxamine A1	0.98529 (max of 14)	14	G7; S13750; S12337; S12332; S11873; S11841; S3; S4747; S9861; S10124; S10158; S11442; S11443; S11444	0.026792007	0.10276373
Streptomyces	S11744	Daidzein-4',7-di-alpha-L-rhamnoside	1 (max of 9)	9	S13094; S11974; S11752; S11746; S11745; S4497; S4500; S11126; S11127	0.7124288	-0.660344
Streptomyces	S11745	5-(1",3"-Dimethyl-2"-hydroxyl-butyl)-2-en-2-methyl-3-rhamnosyl-valerolactone	1 (max of 9)	9	S13094; S11974; S11752; S11746; S4497; S4500; S11126; S11127; S11744	0.4402832	-0.6756676
Streptomyces	S11746	4'-O-Methyl-7-O-alpha-L-rhamnopyranosylgenistein	1 (max of 9)	9	S13094; S11974; S11752; S4497; S4500; S11126; S11127; S11744; S11745	0.68937767	-0.6025814

Genus	Comp ID	Name	Neighbor Similarity FragFp 85%	Neighbor Count	Neighbor	Neighbor Analysis X	Neighbor Analysis Y
Streptomyces	S11752	Aturanoside B	0.89641 (max of 12)	12	G14; G13; S13094; S12637; S11974; S3045; S3046; S11126; S11127; S11744; S11745; S11746	0.6589258	-0.5256835
Streptomyces	S11784	DFOB-(SS)1[001]	0.92414 (max of 2)	2	S11873; S3	0.18909363	0.56023896
Streptomyces	S11821	cyclo(Gly-Leu)	1 (max of 6)	6	G9; S13104; S12067; S4070; S4535; S10155	0.45989105	-0.428062
Streptomyces	S11841	Desferrioxamine D1	1 (max of 15)	15	G7; S13750; S12337; S12332; S12329; S11873; S3; S4747; S9861; S10124; S10158; S11442; S11443; S11444; S11729	-0.28910843	0.47796068
Streptomyces	S11873	dDesferrioxamine[00-]	1 (max of 18)	18	G7; S13750; S12337; S12332; S11886; S3; S4747; S6330; S9605; S9861; S10124; S10158; S11442; S11443; S11444; S11729; S11784; S11841	0.13468412	0.40751773
Streptomyces	S11886	dDesferrioxamine[-00]	1 (max of 6)	6	S13751; S12329; S12168; S3; S6330; S11873	-0.018241685	0.7921406
Streptomyces	S11924	FR900452 C	0.86408 (max of 3)	3	G1; S10249; S11573	-0.70599055	0.48441726
Streptomyces	S11974	Flavoside A	0.91791 (max of 9)	9	S13094; S4497; S4500; S11126; S11127; S11744; S11745; S11746; S11752	0.5793186	-0.64809644
Streptomyces	S11980	Fumiquinazoline C	0.87674 (max of 7)	7	G1; S1201; S9103; S9529; S10249; S11343; S11346	-0.4781372	0.81846267
Streptomyces	S1201	Cyclomarin A	0.98744 (max of 9)	9	G1; S11980; S11346; S11345; S11344; S11343; S10249; S9529; S9103	-0.5817795	0.77306074
Streptomyces	S12067	1,4-Diaza-2,5-dioxo-3-isobutylbicyclo[4.3.0]nonane	0.92632 (max of 7)	7	G9; S13104; S3704; S4070; S4535; S10155; S11821	0.22168803	-0.574938
Streptomyces	S12168	N-Hydroxy-N-succinylcadaverin	0.97222 (max of 4)	4	S13751; S12329; S6330; S11886	-0.17490122	0.7580717

Genus	Comp ID	Name	Neighbor Similarity FragFp 85%	Neighbor Count	Neighbor	Neighbor Analysis X	Neighbor Analysis Y
Streptomyces	S1217 3	Iturin A	0.97 (max of 8)	8	G8; S12676; S12435; S12393; S5079; S11139; S11140; S11424	0.45022452	0.4632398
Streptomyces	S1231 2	Actinomycin Z6	0.99277 (max of 23)	23	G11; G10; S13171; S13170; S13169; S13168; S13167; S12816; S12720; S12704; S12484; S12315; S12314; S12313; S784; S4774; S5020; S8829; S9167; S9492; S10713; S10830; S11253	-0.55859	-0.606983
Streptomyces	S1231 3	Actinomycin Z1	1 (max of 23)	23	G11; G10; S13171; S13170; S13169; S13168; S13167; S12816; S12720; S12704; S12484; S12315; S12314; S784; S4774; S5020; S8829; S9167; S9492; S10713; S10830; S11253; S12312	- 0.34314618	-0.4656578
Streptomyces	S1231 4	Actinomycin Z3	0.99518 (max of 23)	23	G11; G10; S13171; S13170; S13169; S13168; S13167; S12816; S12720; S12704; S12484; S12315; S784; S4774; S5020; S8829; S9167; S9492; S10713; S10830; S11253; S12312; S12313	- 0.48973635	-0.48744428
Streptomyces	S1231 5	Actinomycin Z5	0.99518 (max of 22)	22	G11; G10; S13171; S13170; S13169; S13168; S13167; S12816; S12720; S12484; S784; S4774; S5020; S8829; S9167; S9492; S10713; S10830; S11253; S12312; S12313; S12314	- 0.18690304	-0.44089746
Streptomyces	S1232 9	Danoxamine	0.97931 (max of 9)	9	G7; S13751; S12332; S4747; S6330; S10124; S11841; S11886; S12168	-0.0726198	0.65349615
Streptomyces	S1233 2	Bisucaberin	1 (max of 15)	15	G7; S13750; S12337; S3; S4747; S9861; S10124; S10158; S11442; S11443; S11444; S11729; S11841; S11873; S12329	- 0.02050724 6	0.39206478
Streptomyces	S1233 7	Desferrioxamine H	0.99248 (max of 14)	14	G7; S13750; S3; S4747; S9861; S10124; S10158; S11442; S11443; S11444; S11729; S11841; S11873; S12332	- 0.06785153 6	0.2275679
Streptomyces	S1239 3	Violacin A	0.89552 (max of 4)	4	G8; S12676; S5079; S12173	0.34192657	0.5813548
Streptomyces	S1243 5	cyclo(3-Hydroxy-L-Pro-L-Tyr)	0.88176 (max of 6)	6	G8; S13629; S2960; S9880; S11139; S12173	0.3387859	0.15595469

Genus	Comp ID	Name	Neighbor Similarity FragFp 85%	Neighbor Count	Neighbor	Neighbor Analysis X	Neighbor Analysis Y
Streptomyces	S12484	RSP 01	0.99754 (max of 24)	24	G11; G10; S13171; S13170; S13169; S13168; S13167; S12816; S12720; S12705; S12704; S784; S4774; S5020; S8829; S9167; S9492; S10713; S10830; S11253; S12312; S12313; S12314; S12315	-0.3799415	-0.047550067
Streptomyces	S12583	Pteridic acid C	0.98429 (max of 5)	5	G26; S12587; S12586; S12585; S12584	0.703108	0.703108
Streptomyces	S12584	Pteridic acid D	0.98429 (max of 5)	5	G26; S12587; S12586; S12585; S12583	0.78642	0.536245
Streptomyces	S12585	Pteridic acid E	0.98404 (max of 5)	5	G26; S12587; S12586; S12583; S12584	0.74860525	0.63106
Streptomyces	S12586	Pteridic acid F	0.98404 (max of 5)	5	G26; S12587; S12583; S12584; S12585	0.689049	0.6333651
Streptomyces	S12587	Pteridic acid G	0.96373 (max of 5)	5	G26; S12583; S12584; S12585; S12586	0.5786106	0.7253358
Streptomyces	S12637	Tetracenoquinocin A	0.9 (max of 7)	7	G14; G13; S3045; S3046; S4497; S4500; S11752	0.60452193	-0.40574428
Streptomyces	S12676	Chaxapeptin	0.89623 (max of 4)	4	G8; S11139; S12173; S12393	0.58592606	0.4815378
Streptomyces	S12704	Neo-actinomycin A	0.978 (max of 20)	20	G11; G10; S13171; S13170; S13169; S13168; S13167; S12720; S12705; S784; S4774; S5020; S8829; S10713; S10830; S11253; S12312; S12313; S12314; S12484	-0.7987305	-0.18698107
Streptomyces	S12705	Neo-actinomycin B	0.978 (max of 11)	11	G11; G10; S12720; S784; S4774; S5020; S8829; S10713; S10830; S12484; S12704	-0.7676029	-0.03180099
Streptomyces	S12720	Actinomycin C2	1 (max of 24)	24	G11; G10; S13171; S13170; S13169; S13168; S13167; S12816; S784; S4774; S5020; S8829; S9167; S9492; S10713; S10830; S11253; S12312; S12313; S12314; S12315; S12484; S12704; S12705	-0.6668176	-0.13326783

Genus	Comp ID	Name	Neighbor Similarity FragFp 85%	Neighbor Count	Neighbor	Neighbor Analysis X	Neighbor Analysis Y
Streptomyces	S12816	Actinomycin X0	0.95411 (max of 21)	21	G11; G10; S13171; S13170; S13169; S13168; S13167; S784; S4774; S5020; S8829; S9492; S10713; S10830; S11253; S12312; S12313; S12314; S12315; S12484; S12720	-0.39853755	-0.6030955
Streptomyces	S13094	6-O-Methyl-7-O-alpha-L-Rhamnopyranosyldaidzein	0.96457 (max of 13)	13	G14; G13; S3045; S3046; S4497; S4500; S11126; S11127; S11744; S11745; S11746; S11752; S11974	0.7619986	-0.49261183
Streptomyces	S13104	(S)-3-Isobutylpiperazine-2,5-dione	1 (max of 6)	6	G9; S4070; S4535; S10155; S11821; S12067	0.1333277	-0.4170768
Streptomyces	S13167	Actinomycin Y6	0.98798 (max of 23)	23	G11; G10; S13171; S13170; S13169; S13168; S784; S4774; S5020; S8829; S9167; S9492; S10713; S10830; S11253; S12312; S12313; S12314; S12315; S12484; S12704; S12720; S12816	-0.2569968	-0.16714546
Streptomyces	S13168	Actinomycin Y7	1 (max of 23)	23	G11; G10; S13171; S13170; S13169; S784; S4774; S5020; S8829; S9167; S9492; S10713; S10830; S11253; S12312; S12313; S12314; S12315; S12484; S12704; S12720; S12816; S13167	-0.58416134	-0.37910146
Streptomyces	S13169	Actinomycin Y8	0.99758 (max of 23)	23	G11; G10; S13171; S13170; S784; S4774; S5020; S8829; S9167; S9492; S10713; S10830; S11253; S12312; S12313; S12314; S12315; S12484; S12704; S12720; S12816; S13167; S13168	-0.24977517	-0.582982
Streptomyces	S13170	Actinomycin Y9	1 (max of 23)	23	G11; G10; S13171; S784; S4774; S5020; S8829; S9167; S9492; S10713; S10830; S11253; S12312; S12313; S12314; S12315; S12484; S12704; S12720; S12816; S13167; S13168; S13169	-0.28435573	-0.30956993
Streptomyces	S13171	Actinomycin Zp	1 (max of 23)	23	G11; G10; S784; S4774; S5020; S8829; S9167; S9492; S10713; S10830; S11253; S12312; S12313; S12314; S12315; S12484; S12704;	-0.444883	-0.34875205

Genus	Comp ID	Name	Neighbor Similarity FragFp 85%	Neighbor Count	Neighbor	Neighbor Analysis X	Neighbor Analysis Y
					S12720; S12816; S13167; S13168; S13169; S13170		
Streptomyces	S13629	cyclo(D- Pro- L-Tyr)	1 (max of 5)	5	G8; S2960; S9880; S11139; S12435	0.4452466	0.031679522
Streptomyces	S13652	6-O-alpha-L-Rhamnosyl-8-O-methyltetrangomycin	0.89399 (max of 4)	4	G14; G13; S3045; S3046	0.8808145	-0.1319048
Streptomyces	S13750	Ferrioxamine D	1 (max of 15)	15	G7; S13751; S3; S4747; S9861; S10124; S10158; S11442; S11443; S11444; S11729; S11841; S11873; S12332; S12337	-0.17576519	0.36160854
Streptomyces	S13751	Ferrioxamine G	0.9931 (max of 7)	7	S6330; S11443; S11444; S11886; S12168; S12329; S13750	-0.22965114	0.6221816
Streptomyces	S2944	Pimprinine	1 (max of 2)	2	G2; S4820	0.16401725	0.12021532
Streptomyces	S2960	Maculosin	1 (max of 5)	5	G8; S13629; S12435; S11139; S9880	0.51093715	0.18956713
Streptomyces	S2987	Fujianmycin A	1 (max of 4)	4	G12; S11317; S11316; S2988	-0.012991432	-0.7382032
Streptomyces	S2988	Fujianmycin B	0.95266 (max of 4)	4	G12; S11317; S11316; S2987	0.13960885	-0.69318783
Streptomyces	S3	Deferoxamine	1 (max of 18)	18	G7; S13750; S12337; S12332; S11886; S11873; S11841; S11784; S11729; S11444; S11443; S11442; S10158; S10124; S9861; S9605; S6330; S4747	0.032133173	0.550889
Streptomyces	S3045	Atramycin A	1 (max of 9)	9	G14; G13; S13652; S13094; S12637; S11752; S10808; S4497; S3046	0.8407525	-0.40194076
Streptomyces	S3046	Atramycin B	1 (max of 9)	9	G14; G13; S13652; S13094; S12637; S11752; S10808; S4497; S3045	0.78669053	-0.21571176
Streptomyces	S3704	L-Prolyl-L-Leucylglycinamide	0.90547 (max of 5)	5	G9; S12067; S10155; S4535; S4070	0.3712463	-0.55636734

Genus	Comp ID	Name	Neighbor Similarity FragFp 85%	Neighbor Count	Neighbor	Neighbor Analysis X	Neighbor Analysis Y
Streptomyces	S3837	Diolmycin B1	1 (max of 3)	3	G20; G19; G15	0.7917172	0.09231631
Streptomyces	S3838	Diolmycin A1	1 (max of 2)	2	G5; G6	-0.17413989	-0.71222836
Streptomyces	S4070	cyclo(Leu-Ala)	1 (max of 11)	11	G9; S13104; S12067; S11821; S11693; S10155; S9853; S9704; S4896; S4535; S3704	0.21320711	-0.28036204
Streptomyces	S4497	5,7,4'-Trihydroxyisoflavone 3'-O-alpha-L-rhamnopyranoside	0.99593 (max of 13)	13	G14; G13; S13094; S12637; S11974; S11746; S11745; S11744; S11127; S11126; S4500; S3045; S3046	0.7888281	-0.5534633
Streptomyces	S4500	7,4'-Dihydroxyisoflavone 3'-O-alpha-L-rhamnopyranoside	0.99593 (max of 9)	9	S13094; S12637; S11974; S11746; S11745; S11744; S11127; S11126; S4497	0.48838004	-0.7548216
Streptomyces	S4535	cyclo(L-Leu-L-Arg)	0.92 (max of 7)	7	G9; S13104; S12067; S11821; S10155; S3704; S4070	0.5266572	-0.28244478
Streptomyces	S4747	Nocardamin	1 (max of 15)	15	G7; S13750; S12337; S12332; S12329; S11873; S11841; S11729; S11444; S11443; S11442; S10158; S10124; S9861; S3	-0.1273219	0.5081587
Streptomyces	S4774	Actinomycin C3	1 (max of 24)	24	G11; G10; S13171; S13170; S13169; S13168; S13167; S12816; S12720; S12705; S12704; S12484; S12315; S12314; S12313; S12312; S11253; S10830; S10713; S9492; S9167; S8829; S5020; S784	-0.76391506	-0.37682572
Streptomyces	S4820	Streptochlorin	0.90517 (max of 2)	2	G2; S2944	0.032628205	-0.05191145
Streptomyces	S4896	ZINC04026203	0.86792 (max of 3)	3	G9; S10155; S4070	0.46832615	-0.13233568
Streptomyces	S5020	Actinomycin X2	1 (max of 24)	24	G11; G10; S13171; S13170; S13169; S13168; S13167; S12816; S12720; S12705; S12704; S12484; S12315; S12314; S12313; S12312;	-0.6677032	-0.49207962

Genus	Comp ID	Name	Neighbor Similarity FragFp 85%	Neighbor Count	Neighbor	Neighbor Analysis X	Neighbor Analysis Y
					S11253; S10830; S10713; S9492; S9167; S8829; S784; S4774		
Streptomyces	S5050	16-Hydroxyprogesterone	0.875	1	G29	0.7057764	-0.7057764
Streptomyces	S5079	Aspartocin	0.92532 (max of 6)	6	G8; S12393; S12173; S11424; S11140; S11139	0.49313006	0.6079512
Streptomyces	S6330	Deferrioxamine G	1 (max of 6)	6	S13751; S12329; S12168; S11886; S11873; S3	0.086262	0.6800615
Streptomyces	S784	Actinomycin	1 (max of 24)	24	G11; G10; S13171; S13170; S13169; S13168; S13167; S12816; S12720; S12705; S12704; S12484; S12315; S12314; S12313; S12312; S11253; S10830; S10713; S9492; S9167; S8829; S5020; S4774	-0.53071433	-0.088407926
Streptomyces	S8829	Actinomycin C	1 (max of 24)	24	G11; G10; S13171; S13170; S13169; S13168; S13167; S12816; S12720; S12705; S12704; S12484; S12315; S12314; S12313; S12312; S11253; S10830; S10713; S9492; S9167; S784; S4774; S5020	-0.22066319	-0.013414034
Streptomyces	S9103	O-Methyl-borregomycin A	0.99533 (max of 9)	9	G1; S11980; S11343; S10475; S10252; S10249; S10248; S9529; S1201	-0.4188316	0.77638626
Streptomyces	S9167	Methylated actinomycin D	0.97229 (max of 21)	21	G11; G10; S13171; S13170; S13169; S13168; S13167; S12720; S12484; S12315; S12314; S12313; S12312; S11253; S10830; S10713; S9492; S784; S4774; S5020; S8829	-0.109322764	-0.119851254
Streptomyces	S9492	C-Demethylactinomycin	0.97229 (max of 22)	22	G11; G10; S13171; S13170; S13169; S13168; S13167; S12816; S12720; S12484; S12315; S12314; S12313; S12312; S11253; S10830; S10713; S784; S4774; S5020; S8829; S9167	-0.13846156	-0.2869358
Streptomyces	S9529	Borregomycin A	0.99533 (max of 9)	9	G1; S11980; S11343; S10475; S10252; S10249; S10248; S1201; S9103	-0.5039797	0.6644496

Genus	Comp ID	Name	Neighbor Similarity FragFp 85%	Neighbor Count	Neighbor	Neighbor Analysis X	Neighbor Analysis Y
Streptomyces	S9605	Acyl-desferrioxamine	0.92414 (max of 2)	2	S11873; S3	0.23343231	0.27764782
Streptomyces	S9704	(3S,6S)-3,6-Bis(4-acetamidobutyl)piperazine-2,5-dione	0.86979 (max of 3)	3	G9; S10155; S4070	0.054900188	-0.27556005
Streptomyces	S9853	3-Isopropyl-6-(2-methyl-propyl)-2,5-piperazinedione	1 (max of 4)	4	G9; S11693; S10155; S4070	0.13221174	-0.12788527
Streptomyces	S9861	Ferrioxamine E	0.99248 (max of 14)	14	G7; S13750; S12337; S12332; S11873; S11841; S11729; S11444; S11443; S11442; S10158; S10124; S3; S4747	-0.11233599	0.07859782
Streptomyces	S9880	NSC646120	1 (max of 5)	5	G8; S13629; S12435; S11139; S2960	0.6182173	0.070890464

Table S6. Datawarrior and SwissADME physicochemical parameters.

Due to the size of this data, the table is not presented in this file. The table can be consulted and downloaded at the following link: https://osf.io/sp3w9.

Table S7. Roary presence/absence table.

Due to the size of this data, the table is not presented in this file. The table can be consulted and downloaded at the following link: https://osf.io/v6czp.

Table S8. Similarity matrix between the core genome sequences.

Due to the size of this data, the table is not presented in this file. The table can be consulted and downloaded at the following link: https://osf.io/xr2dh.

Table S9. Results of mining *Gordonia* reference genomes by the antiSMASH tool.

Due to the size of this data the table, is not presented in this file. The table can be consulted and downloaded at the following link: https://osf.io/6ymte.

Table S10. Results of mining *G. polyisoprenivorans, G. rubripertincta,* and *G. terrae* genomes by the antiSMASH tool.

Due to the size of this data the table, is not presented in this file. The table can be consulted and downloaded at the following link: https://osf.io/a7bj5.

Table S11. Clustering by family of BGCs detected in Gordonia genomes by the BiG-Scape tool.

BGC Family	NRPS	Others	PKS-I	PKS-NRSP	PKSOther	RiPPs	Terpene
**1**	1	0	0	0	0	0	0
**3**	0	0	0	0	0	0	1
**4**	1	0	0	0	0	0	0
**5**	0	0	0	0	0	0	1
**6**	1	0	0	0	0	0	0
**7**	0	1	0	0	0	0	0
**8**	1	0	0	0	0	0	0
**9**	1	0	0	0	0	0	0
**11**	0	1	0	0	0	0	0
**13**	0	0	0	0	0	1	0
**15**	1	0	0	0	0	0	0
**17**	0	1	0	0	0	0	0

BGC Family	NRPS	Others	PKS-I	PKS-NRSP	PKSOther	RiPPs	Terpene
**18**	1	1	0	0	0	0	1
**19**	1	0	0	0	0	0	0
**23**	0	0	0	0	1	0	0
**24**	1	0	0	0	0	0	0
**25**	0	0	0	0	0	0	1
**26**	0	1	0	0	0	0	0
**27**	0	0	0	0	0	0	1
**28**	1	0	0	0	0	0	0
**29**	0	0	0	0	0	0	13
**31**	1	0	0	0	0	0	0
**32**	2	0	0	0	0	0	0
**36**	0	1	0	0	0	0	0
**37**	0	1	0	0	0	0	0
**38**	0	1	0	0	0	0	0
**39**	1	0	0	0	0	0	0
**40**	1	0	0	0	0	0	0
**42**	0	0	0	0	0	0	1
**44**	1	0	0	0	0	0	0
**52**	1	0	0	0	0	0	0
**53**	3	0	0	0	0	0	0
**56**	2	2	0	0	0	0	0
**58**	1	0	0	0	0	0	0
**60**	0	5	0	0	0	0	0
**61**	0	0	0	0	0	0	6
**63**	8	0	0	0	0	0	0
**65**	1	1	0	0	0	0	0
**66**	1	1	0	0	0	0	0
**67**	1	0	0	0	0	0	0
**68**	1	0	0	0	0	0	0
**69**	0	1	0	0	0	0	0
**71**	0	0	0	0	0	1	0
**72**	0	0	0	0	0	0	2
**73**	0	0	0	0	0	1	0

BGC Family	NRPS	Others	PKS-I	PKS-NRSP	PKSOther	RiPPs	Terpene
**74**	0	0	0	0	0	0	1
**75**	1	0	0	0	0	0	0
**76**	1	0	0	0	0	0	0
**77**	1	0	0	0	0	0	0
**78**	1	0	0	0	0	0	0
**80**	0	1	0	0	0	0	0
**82**	0	1	0	0	0	0	0
**84**	1	0	0	0	0	0	0
**86**	1	0	0	0	0	0	0
**87**	0	0	0	1	1	0	0
**88**	1	0	0	0	0	0	0
**90**	1	0	0	0	0	0	0
**91**	0	0	1	0	0	0	0
**92**	0	0	1	0	0	0	0
**93**	0	0	0	0	0	6	0
**95**	1	0	0	0	0	0	0
**97**	1	0	0	0	0	0	0
**99**	0	1	0	0	0	0	0
**102**	1	0	0	0	0	0	0
**103**	0	1	0	0	0	0	0
**107**	0	0	0	0	0	0	1
**109**	4	0	0	0	0	0	0
**110**	0	0	0	0	0	1	0
**113**	0	11	0	0	0	0	0
**114**	1	0	0	0	0	0	0
**115**	1	0	0	0	0	0	0
**116**	0	20	0	0	0	0	0
**119**	1	0	0	0	0	0	0
**122**	0	0	0	0	0	2	0
**124**	1	0	0	0	0	0	0
**126**	1	0	0	0	0	0	0
**127**	0	0	6	0	0	0	0
**128**	0	1	0	0	0	0	0

BGC Family	NRPS	Others	PKS-I	PKS-NRSP	PKSOther	RiPPs	Terpene
**129**	1	0	0	0	0	0	0
**130**	0	1	0	0	0	0	0
**132**	1	0	0	0	0	0	0
**134**	0	0	0	0	1	0	0
**135**	1	0	0	0	0	0	0
**136**	0	0	0	0	0	0	1
**138**	0	1	0	0	0	0	0
**150**	1	0	0	0	0	0	0
**152**	2	0	0	0	0	0	0
**153**	1	0	0	0	0	0	0
**154**	2	0	0	0	0	0	0
**157**	12	0	0	0	0	0	0
**158**	3	0	0	0	0	0	0
**160**	1	0	0	0	0	0	0
**161**	1	0	0	0	0	0	0
**162**	0	0	0	0	0	0	1
**165**	1	0	0	0	0	0	0
**168**	4	0	0	0	0	0	0
**171**	0	1	0	0	0	0	0
**172**	1	0	0	0	0	0	0
**173**	1	0	0	0	0	0	0
**174**	0	3	0	0	0	0	0
**175**	0	1	0	0	0	0	0
**176**	2	0	0	0	0	0	0
**180**	1	0	0	0	0	0	0
**181**	1	0	0	0	0	0	0
**182**	0	0	0	0	0	0	4
**184**	1	0	0	0	0	0	0
**189**	1	0	0	0	0	0	0
**190**	5	0	0	0	0	0	0
**191**	1	0	0	0	0	0	0
**192**	1	0	0	0	0	0	0
**193**	0	0	0	0	0	0	1

BGC Family	NRPS	Others	PKS-I	PKS-NRSP	PKSOther	RiPPs	Terpene
195	0	1	0	0	0	0	0
196	0	1	0	0	0	0	0
197	1	0	0	0	0	0	0
199	0	0	0	0	1	0	0
200	1	0	0	0	0	0	0
203	0	1	0	0	0	0	0
204	0	0	0	0	1	0	0
206	1	1	0	0	0	0	0
207	1	0	0	0	0	0	0
209	1	0	0	0	0	0	0
210	1	0	0	0	0	0	0
211	1	0	0	0	0	0	0
212	1	0	0	0	0	0	0
213	1	0	0	0	0	0	0
214	1	1	0	0	0	0	0
216	1	0	0	0	0	0	0
218	0	1	0	0	0	0	0
220	0	0	0	0	0	0	1
222	1	0	0	0	0	0	0
224	0	0	0	0	0	1	0
225	1	0	0	0	0	0	0
226	0	1	0	0	0	0	0
229	1	0	0	0	0	0	0
231	1	0	0	0	0	0	0
233	0	1	0	0	0	0	0
234	1	0	0	0	0	0	0
235	0	0	0	0	0	1	0
237	1	0	0	0	0	0	0
238	0	0	0	0	0	0	1
239	1	0	0	0	0	0	0
240	1	0	0	0	0	0	0
241	1	0	0	0	0	0	0
242	0	0	25	0	0	0	0

BGC Family	NRPS	Others	PKS-I	PKS-NRSP	PKSOther	RiPPs	Terpene
**244**	1	0	0	0	0	0	0
**245**	0	0	0	0	0	5	0
**246**	1	1	0	0	0	0	0
**247**	1	0	0	0	0	0	0
**248**	0	1	0	0	0	0	0
**253**	1	0	0	0	0	0	0
**254**	0	0	0	0	1	0	0
**255**	1	0	0	0	0	0	0
**256**	1	0	0	0	0	0	0
**258**	0	0	2	0	0	0	0
**259**	1	1	0	0	0	0	0
**260**	1	0	0	0	0	0	0
**261**	0	1	0	0	0	0	0
**262**	1	0	0	0	0	0	0
**263**	0	0	0	0	0	1	0
**264**	0	1	0	0	0	0	0
**266**	0	0	0	0	0	0	1
**267**	1	0	0	0	0	0	0
**268**	1	1	0	0	0	0	0
**269**	1	1	0	0	0	0	0
**270**	0	2	0	0	0	0	0
**272**	0	1	0	0	0	0	0
**273**	1	0	0	0	0	0	0
**274**	1	0	0	0	0	0	0
**277**	0	0	0	0	0	0	1
**278**	0	0	0	0	1	0	0
**279**	1	0	0	0	0	0	0
**280**	0	1	0	0	0	0	0
**282**	0	0	0	0	0	0	1
**283**	0	0	0	0	0	0	1
**284**	0	1	0	0	0	0	0
**285**	0	1	0	0	0	0	0
**286**	1	0	0	0	0	0	0

BGC Family	NRPS	Others	PKS-I	PKS-NRSP	PKSOther	RiPPs	Terpene
**287**	0	0	0	0	0	1	0
**288**	1	1	0	0	0	0	0
**289**	0	0	1	0	0	0	0
**290**	1	0	0	0	0	0	0
**291**	0	1	0	0	0	0	0
**292**	0	1	0	0	0	0	0
**297**	1	0	0	0	0	0	0
**299**	1	1	0	0	0	0	0
**300**	1	0	0	0	0	0	0
**309**	1	0	0	0	0	0	0
**314**	1	0	0	0	0	0	0
**317**	1	0	0	0	0	0	0
**319**	0	0	0	0	0	0	3
**321**	0	1	0	0	0	0	0
**322**	1	1	0	0	0	0	0
**323**	1	0	0	0	0	0	0
**327**	0	0	0	0	0	0	1
**328**	1	0	0	0	0	0	0
**329**	1	0	0	0	0	0	0
**330**	1	0	0	0	0	0	0
**332**	1	0	0	0	0	0	0
**334**	0	0	0	0	0	0	1
**336**	1	0	0	0	0	0	0
**338**	1	0	0	0	0	0	0
**340**	2	0	0	0	0	0	0
**342**	1	0	0	0	0	0	0
**343**	1	1	0	0	0	0	0
**346**	1	0	0	0	0	0	0
**348**	1	1	0	0	0	0	0
**349**	1	0	0	0	0	0	0
**352**	1	0	0	0	0	0	0
**353**	0	1	0	0	0	0	0
**355**	0	0	0	0	1	0	0

BGC Family	NRPS	Others	PKS-I	PKS-NRSP	PKSOther	RiPPs	Terpene
**356**	2	2	0	0	0	0	2
**357**	1	0	0	0	0	0	0
**358**	0	0	0	0	0	0	2
**359**	1	1	0	0	0	0	0
**360**	0	0	0	0	2	0	0
**361**	0	2	0	0	0	0	0
**362**	2	0	0	1	1	0	0
**364**	0	0	0	0	0	9	0
**368**	1	0	0	0	0	0	0
**369**	0	1	0	0	0	0	0
**371**	0	0	0	0	1	0	0
**372**	1	0	0	0	0	0	0
**374**	1	0	0	0	0	0	0
**376**	0	1	0	0	0	0	0
**377**	1	1	0	0	0	0	0
**378**	1	0	0	0	0	0	0
**380**	1	0	0	0	0	0	0
**383**	9	9	0	0	0	0	0
**384**	1	0	0	0	0	0	0
**387**	0	1	0	0	0	0	0
**389**	1	0	0	0	0	0	0
**402**	2	0	0	0	0	0	0
**411**	5	6	0	0	0	0	0
**412**	2	0	0	0	0	0	0
**414**	7	0	0	3	3	0	0
**421**	4	0	0	0	0	0	0
**426**	1	0	0	0	0	0	0
**430**	1	0	0	0	0	0	0
**431**	0	0	0	0	0	0	4
**436**	1	0	0	0	0	0	0
**437**	0	1	0	0	0	0	0
**440**	0	2	0	0	0	0	0
**445**	0	0	0	0	0	0	3

BGC Family	NRPS	Others	PKS-I	PKS-NRSP	PKSOther	RiPPs	Terpene
**449**	1	0	0	0	0	0	0
**450**	1	1	0	0	0	0	0
**452**	1	0	0	0	0	0	0
**454**	0	0	3	0	0	0	0
**455**	0	0	0	0	0	0	1
**456**	2	0	0	0	0	0	0
**458**	2	0	0	0	0	0	0
**459**	0	1	0	0	0	0	0
**465**	1	1	0	0	0	0	0
**467**	1	0	0	0	0	0	0
**468**	1	1	0	0	0	0	0
**469**	1	1	0	0	0	0	0
**471**	1	0	0	1	1	0	0
**472**	0	0	0	0	1	0	0
**473**	0	0	0	0	0	0	1
**475**	0	1	0	0	0	0	0
**476**	1	0	0	0	0	0	0
**480**	0	0	0	2	2	0	0
**482**	2	0	0	0	0	0	0
**486**	1	0	0	0	0	0	0
**487**	2	0	0	0	0	0	0
**490**	2	0	0	0	0	0	0
**491**	1	0	0	0	0	0	0
**495**	0	4	0	0	0	0	0
**496**	1	0	0	0	0	0	0
**497**	1	0	0	0	0	0	0
**498**	0	0	0	0	0	18	0
**499**	0	0	0	0	0	0	1
**501**	1	0	0	0	0	0	0
**502**	0	0	0	0	0	1	0
**503**	0	1	0	0	0	0	0
**505**	1	0	0	0	0	0	0
**507**	0	0	0	0	0	0	16

BGC Family	NRPS	Others	PKS-I	PKS-NRSP	PKSOther	RiPPs	Terpene
**509**	0	14	0	0	0	0	0
**510**	1	0	0	0	0	0	0
**511**	0	11	0	0	0	0	0
**518**	0	1	0	0	0	0	0
**520**	1	0	0	0	0	0	0
**521**	2	0	0	1	1	0	0
**522**	0	0	0	0	0	0	2
**523**	1	0	0	0	0	0	0
**524**	0	0	0	0	0	0	1
**525**	1	0	0	0	0	0	0
**526**	0	1	0	0	0	0	0
**527**	1	0	0	0	0	0	0
**528**	1	0	0	0	0	0	0
**530**	0	1	0	0	0	0	0

Table S12. Network data by similarity of BGCs detected in Gordonia genomes built by the BiG-Scape tool.

BGC_1	BGC_2	Raw distance	Squared similarity	Jaccard index	DSS index	Adjacency index	raw DSS non-anchor	raw DSS anchor	Non-anchor domains	Anchor domains	Network
BGC_124_rhiz	BGC_397_rubr	0.270611	0.532008	0.40625	0.729389	0.294118	0.281238	0.236074	13	1	NRPS
BGC_303_alka	BGC_402_rubr	0.15924	0.706878	0.761905	0.84076	0.734694	0.211095	0.052936	41	5	NRPS
BGC_307_alka	BGC_439_lacu	0.161266	0.703475	0.758621	0.838734	0.65625	0.173075	0.090411	24	1	NRPS
BGC_445_lacu	BGC_518_para	0.231838	0.590073	0.861111	0.768162	0.76087	0.228312	0.237127	36	6	NRPS
BGC_155_nami	BGC_400_rubr	0.133101	0.751514	0.891304	0.866899	0.851852	0.074117	0.194353	54	13	NRPS
BGC_121_sput	BGC_435_jaco	0.135484	0.747388	0.884615	0.864516	0.6875	0.136441	0.128788	28	1	NRPS
BGC_158_nami	BGC_517_para	0.172804	0.684254	0.774194	0.827196	0.666667	0.174049	0.165333	24	1	NRPS
BGC_110_sput	BGC_425_jaco	0.199952	0.640076	0.916667	0.800048	0.844444	0.07609	0.280807	47	18	NRPS
BGC_314_alka	BGC_412_west	0.150299	0.721992	0.666667	0.849701	0.653846	0.121684	0.168183	50	20	NRPS
BGC_065_amic	BGC_150_nami	0.268483	0.535117	0.852941	0.731517	0.794872	0.250386	0.30807	35	4	NRPS
BGC_420_west	BGC_491_terr	0.223311	0.603245	0.88	0.776689	0.806452	0.198947	0.454774	38	1	NRPS
BGC_056_amic	BGC_413_west	0.286775	0.50869	0.894737	0.713225	0.769231	0.102828	0.38711	24	11	NRPS
BGC_124_rhiz	BGC_418_west	0.26645	0.538096	0.40625	0.73355	0.285714	0.280331	0.221333	13	1	NRPS
BGC_102_otit	BGC_121_sput	0.117596	0.778637	1	0.882404	1	0.10703	0.1625	17	1	NRPS
BGC_144_nami	BGC_395_rubr	0.048991	0.904419	1	0.951009	1	0.039822	0.058784	47	11	NRPS
BGC_363_amar	BGC_366_pseu	0.234685	0.585707	0.833333	0.765315	0.65	0.245397	0.140957	35	1	NRPS
BGC_158_nami	BGC_397_rubr	0.073599	0.858219	0.90625	0.926401	0.794118	0.070509	0.096774	30	1	NRPS
BGC_303_alka	BGC_414_west	0.223714	0.602619	0.846154	0.776286	0.765957	0.105712	0.436118	36	5	NRPS
BGC_414_west	BGC_445_lacu	0.275036	0.525573	0.780488	0.724964	0.7	0.297314	0.229366	41	5	NRPS
BGC_121_sput	BGC_169_aich	0.226715	0.59797	0.84	0.773285	0.59375	0.234871	0.16962	28	1	NRPS
BGC_063_amic	BGC_153_nami	0.22094	0.606934	0.225806	0.77906	0.1875	0.312304	0.17069	11	5	NRPS
BGC_384_jing	BGC_445_lacu	0.264068	0.541596	0.888889	0.735932	0.8	0.185968	0.384471	37	6	NRPS
BGC_051_amic	BGC_397_rubr	0.259907	0.547738	0.666667	0.740093	0.461538	0.281793	0.117647	26	1	NRPS

BGC_1	BGC_2	Raw distance	Squared similarity	Jaccard index	DSS index	Adjacency index	raw DSS non-anchor	raw DSS anchor	Non-anchor domains	Anchor domains	Network
BGC_058_amic	BGC_415_west	0.132353	0.752811	0.862069	0.867647	0.732394	0.130676	0.139795	71	4	NRPS
BGC_397_rubr	BGC_517_para	0.210345	0.623555	0.757576	0.789655	0.611111	0.218807	0.153226	27	1	NRPS
BGC_308_alka	BGC_420_west	0.19774	0.643621	0.829268	0.80226	0.666667	0.177239	0.218241	48	12	NRPS
BGC_389_jing	BGC_412_west	0.282026	0.515486	0.829787	0.717974	0.732143	0.273014	0.289461	66	20	NRPS
BGC_088_effu	BGC_522_aspl	0.272044	0.52992	0.653846	0.727956	0.540984	0.265479	0.278937	42	10	NRPS
BGC_158_nami	BGC_493_terr	0.283955	0.512721	0.628571	0.716045	0.5	0.304746	0.143617	27	1	NRPS
BGC_124_rhiz	BGC_514_hank	0.28733	0.507898	0.821429	0.71267	0.65625	0.299296	0.209549	26	1	NRPS
BGC_314_alka	BGC_407_rubr	0.114769	0.783633	0.612245	0.885231	0.6	0.109203	0.118109	48	20	NRPS
BGC_065_amic	BGC_303_alka	0.247156	0.566774	0.767442	0.752844	0.705882	0.228967	0.284443	41	5	NRPS
BGC_338_insu	BGC_514_hank	0.178538	0.6748	0.966667	0.821462	0.90625	0.184099	0.134048	32	1	NRPS
BGC_065_amic	BGC_414_west	0.26431	0.541239	0.767442	0.73569	0.686275	0.22958	0.333771	40	5	NRPS
BGC_420_west	BGC_490_terr	0.29485	0.497236	0.775	0.70515	0.627451	0.190122	0.384305	41	12	NRPS
BGC_064_amic	BGC_144_nami	0.110272	0.791616	0.974359	0.889728	0.930233	0.094683	0.127278	48	11	NRPS
BGC_169_aich	BGC_435_jaco	0.195069	0.647914	0.833333	0.804931	0.538462	0.201568	0.144703	31	1	NRPS
BGC_397_rubr	BGC_418_west	0.107031	0.797393	1	0.892969	0.96875	0.106707	0.109626	32	1	NRPS
BGC_514_hank	BGC_517_para	0.280257	0.51803	0.375	0.719743	0.294118	0.297566	0.224	13	1	NRPS
BGC_057_amic	BGC_152_nami	0.171533	0.686358	0.740741	0.828467	0.636364	0.184935	0.162597	32	12	NRPS
BGC_383_jing	BGC_415_west	0.25026	0.562109	0.617647	0.74974	0.45	0.245188	0.292105	33	1	NRPS
BGC_308_alka	BGC_490_terr	0.277163	0.522493	0.780488	0.722837	0.62	0.23906	0.315266	44	11	NRPS
BGC_414_west	BGC_518_para	0.250636	0.561546	0.846154	0.749364	0.826087	0.26233	0.227248	40	5	NRPS
BGC_307_alka	BGC_397_rubr	0.138604	0.742003	0.939394	0.861396	0.805556	0.146643	0.07027	34	1	NRPS
BGC_065_amic	BGC_402_rubr	0.185925	0.662718	0.95	0.814075	0.851064	0.141589	0.276813	41	5	NRPS
BGC_401_rubr	BGC_481_terr	0.298863	0.491593	0.767857	0.701137	0.625	0.338737	0.116938	73	4	NRPS
BGC_391_jing	BGC_397_rubr	0.23609	0.583558	0.857143	0.76391	0.756757	0.247464	0.13941	34	1	NRPS

BGC_1	BGC_2	Raw distance	Squared similarity	Jaccard index	DSS index	Adjacency index	raw DSS non-anchor	raw DSS anchor	Non-anchor domains	Anchor domains	Network
BGC_418_west	BGC_493_terr	0.283165	0.513852	0.647059	0.716835	0.540541	0.305415	0.132979	27	1	NRPS
BGC_391_jing	BGC_418_west	0.247836	0.565751	0.857143	0.752164	0.736842	0.264638	0.100817	35	1	NRPS
BGC_054_amic	BGC_419_west	0.173474	0.683145	0.727273	0.826526	0.407407	0.164623	0.208877	16	1	NRPS
BGC_397_rubr	BGC_493_terr	0.255573	0.554172	0.647059	0.744427	0.555556	0.276231	0.121294	26	1	NRPS
BGC_439_lacu	BGC_493_terr	0.108364	0.795014	0.9	0.891636	0.757576	0.112616	0.07754	29	1	NRPS
BGC_401_rubr	BGC_415_west	0.118961	0.77623	0.862069	0.881039	0.760563	0.118322	0.121794	71	4	NRPS
BGC_049_shan	BGC_295_phth	0.282595	0.514669	0.189189	0.717405	0.131579	0.33415	0.205263	6	1	NRPS
BGC_482_terr	BGC_518_para	0.289561	0.504724	0.861111	0.710439	0.73913	0.230692	0.377864	36	6	NRPS
BGC_101_otit	BGC_425_jaco	0.238618	0.579702	0.885714	0.761382	0.818182	0.161425	0.291689	44	16	NRPS
BGC_412_west	BGC_463_para	0.279863	0.518598	0.833333	0.720137	0.683333	0.24112	0.310857	64	20	NRPS
BGC_384_jing	BGC_482_terr	0.253208	0.557698	0.842105	0.746792	0.782609	0.231933	0.294695	39	5	NRPS
BGC_338_insu	BGC_397_rubr	0.27506	0.525538	0.53125	0.72494	0.484848	0.294255	0.188679	18	1	NRPS
BGC_102_otit	BGC_435_jaco	0.186288	0.662127	0.882353	0.813712	0.789474	0.196897	0.135897	19	1	NRPS
BGC_391_jing	BGC_439_lacu	0.166383	0.694917	0.862069	0.833617	0.71875	0.180508	0.071038	27	1	NRPS
BGC_144_nami	BGC_420_west	0.191597	0.653516	0.85	0.808403	0.68	0.163009	0.219589	47	12	NRPS
BGC_391_jing	BGC_493_terr	0.139867	0.739829	0.806452	0.860133	0.647059	0.145385	0.104	26	1	NRPS
BGC_396_rubr	BGC_419_west	0.243033	0.572999	0.727273	0.756967	0.578947	0.222399	0.289459	27	3	NRPS
BGC_150_nami	BGC_482_terr	0.269238	0.534012	0.870968	0.730762	0.783784	0.221729	0.358318	30	4	NRPS
BGC_033_neof	BGC_186_sihw	0.272965	0.52858	0.83871	0.727035	0.777778	0.329077	0.22578	37	11	NRPS
BGC_102_otit	BGC_169_aich	0.169265	0.69012	0.882353	0.830735	0.789474	0.181508	0.111111	19	1	NRPS
BGC_021_poly	BGC_457_oryz	0.288001	0.506943	0.732143	0.711999	0.590909	0.31261	0.275563	93	46	NRPS
BGC_150_nami	BGC_518_para	0.291092	0.502551	0.966667	0.708908	0.888889	0.166898	0.477382	30	5	NRPS
BGC_158_nami	BGC_439_lacu	0.205165	0.631763	0.758621	0.794836	0.625	0.221647	0.106267	24	1	NRPS
BGC_150_nami	BGC_303_alka	0.076545	0.852768	0.966667	0.923455	0.914286	0.058264	0.110822	30	4	NRPS

BGC_1	BGC_2	Raw distance	Squared similarity	Jaccard index	DSS index	Adjacency index	raw DSS non-anchor	raw DSS anchor	Non-anchor domains	Anchor domains	Network
BGC_418_west	BGC_517_para	0.223654	0.602713	0.757576	0.776346	0.594595	0.231985	0.165333	28	1	NRPS
BGC_307_alka	BGC_517_para	0.183594	0.666519	0.741935	0.816406	0.666667	0.188346	0.15508	24	1	NRPS
BGC_147_nami	BGC_408_rubr	0.11989	0.774593	0.756757	0.88011	0.744186	0.058274	0.161289	43	16	NRPS
BGC_384_jing	BGC_402_rubr	0.2604	0.547008	0.785714	0.7396	0.72	0.265927	0.249623	39	5	NRPS
BGC_440_lacu	BGC_488_terr	0.134512	0.74907	0.830508	0.865488	0.820895	0.179057	0.109576	103	46	NRPS
BGC_338_insu	BGC_439_lacu	0.288025	0.506908	0.448276	0.711975	0.34375	0.312869	0.201072	14	1	NRPS
BGC_149_nami	BGC_415_west	0.170082	0.688763	0.823529	0.829918	0.625	0.14995	0.381471	42	1	NRPS
BGC_436_lacu	BGC_481_terr	0.074245	0.857022	0.933333	0.925755	0.835616	0.087183	0.014408	74	4	NRPS
BGC_338_insu	BGC_517_para	0.295344	0.49654	0.40625	0.704656	0.323529	0.315728	0.224	14	1	NRPS
BGC_051_amic	BGC_158_nami	0.274931	0.525725	0.657143	0.725069	0.5	0.299844	0.100543	28	1	NRPS
BGC_058_amic	BGC_401_rubr	0.115588	0.782185	1	0.884413	0.923077	0.123833	0.07745	74	4	NRPS
BGC_124_rhiz	BGC_158_nami	0.273133	0.528335	0.393939	0.726867	0.285714	0.286798	0.228723	13	1	NRPS
BGC_144_nami	BGC_490_terr	0.2736	0.527657	0.780488	0.7264	0.62	0.235402	0.311798	44	11	NRPS
BGC_384_jing	BGC_414_west	0.288838	0.505752	0.775	0.711162	0.673469	0.197209	0.449187	35	5	NRPS
BGC_015_poly	BGC_459_oryz	0.111117	0.790112	0.818182	0.888883	0.714286	0.125041	0.088927	51	8	NRPS
BGC_142_nami	BGC_422_west	0.213946	0.617881	0.96	0.786054	0.857143	0.135338	0.267989	33	12	NRPS
BGC_418_west	BGC_439_lacu	0.242192	0.574273	0.6875	0.757808	0.647059	0.262816	0.102981	27	1	NRPS
BGC_445_lacu	BGC_482_terr	0.138749	0.741753	0.971429	0.861251	0.952381	0.061395	0.261227	38	6	NRPS
BGC_303_alka	BGC_384_jing	0.28028	0.517997	0.820513	0.71972	0.765957	0.297928	0.244101	41	5	NRPS
BGC_452_lacu	BGC_491_terr	0.1955	0.64722	0.846154	0.8045	0.6	0.154717	0.317848	12	1	NRPS
BGC_118_sput	BGC_159_aich	0.176994	0.677339	0.9	0.823006	0.771429	0.172962	0.193624	33	2	NRPS
BGC_314_alka	BGC_389_jing	0.266718	0.537703	0.543478	0.733282	0.519231	0.227899	0.288068	44	20	NRPS
BGC_110_sput	BGC_164_aich	0.292286	0.50086	0.833333	0.707714	0.717391	0.244731	0.323989	48	18	NRPS
BGC_341_insu	BGC_479_hank	0.295202	0.49674	0.779412	0.704798	0.647059	0.266459	0.308394	123	67	NRPS

BGC_1	BGC_2	Raw distance	Squared similarity	Jaccard index	DSS index	Adjacency index	raw DSS non-anchor	raw DSS anchor	Non-anchor domains	Anchor domains	Network
BGC_142_nami	BGC_408_rubr	0.177491	0.676521	1	0.822509	1	0.222728	0.163354	15	12	NRPS
BGC_158_nami	BGC_391_jing	0.21343	0.618692	0.756757	0.78657	0.585366	0.226203	0.114441	31	1	NRPS
BGC_118_sput	BGC_428_jaco	0.089994	0.828111	1	0.910006	0.967742	0.090221	0.089055	33	2	NRPS
BGC_158_nami	BGC_418_west	0.137498	0.743909	0.90625	0.862502	0.771429	0.14402	0.086957	31	1	NRPS
BGC_408_rubr	BGC_422_west	0.217573	0.612192	0.864865	0.782427	0.68	0.163327	0.24323	70	37	NRPS
BGC_402_rubr	BGC_414_west	0.293936	0.498527	0.756098	0.706064	0.653061	0.216251	0.437652	37	5	NRPS
BGC_158_nami	BGC_307_alka	0.079969	0.846456	0.914286	0.920031	0.837838	0.084017	0.046575	33	1	NRPS
BGC_407_rubr	BGC_412_west	0.188082	0.659211	0.843137	0.811918	0.737705	0.203	0.175215	69	20	NRPS
BGC_049_shan	BGC_191_sihw	0.290469	0.503434	0.222222	0.709531	0.137931	0.362744	0.182058	6	1	NRPS
BGC_051_amic	BGC_391_jing	0.291661	0.501745	0.611111	0.708339	0.363636	0.319297	0.112022	26	1	NRPS
BGC_338_insu	BGC_493_terr	0.290329	0.503633	0.419355	0.709671	0.333333	0.31325	0.210106	14	1	NRPS
BGC_051_amic	BGC_307_alka	0.154764	0.714424	0.742857	0.845236	0.641026	0.165944	0.076503	28	1	NRPS
BGC_303_alka	BGC_518_para	0.284091	0.512526	0.942857	0.715909	0.840909	0.186067	0.43521	37	6	NRPS
BGC_159_aich	BGC_428_jaco	0.181905	0.66928	0.842105	0.818095	0.711111	0.179126	0.196146	41	2	NRPS
BGC_144_nami	BGC_308_alka	0.041959	0.917843	1	0.958041	1	0.03272	0.051828	47	11	NRPS
BGC_150_nami	BGC_402_rubr	0.07865	0.848885	0.84375	0.92135	0.756757	0.060767	0.108828	27	4	NRPS
BGC_314_alka	BGC_463_para	0.294085	0.498316	0.5625	0.705915	0.517857	0.283323	0.300811	50	20	NRPS
BGC_407_rubr	BGC_463_para	0.273024	0.528494	0.851064	0.726976	0.75	0.235135	0.302388	62	20	NRPS
BGC_177_hirs	BGC_325_iter	0.288789	0.505821	0.59375	0.711211	0.472222	0.306892	0.184697	23	1	NRPS
BGC_150_nami	BGC_414_west	0.241957	0.574629	0.965517	0.758043	0.941176	0.115033	0.432342	30	5	NRPS
BGC_308_alka	BGC_395_rubr	0.051082	0.900446	1	0.948918	1	0.034367	0.068936	47	11	NRPS
BGC_051_amic	BGC_418_west	0.208608	0.626301	0.666667	0.791391	0.487179	0.229803	0.070845	26	1	NRPS
BGC_389_jing	BGC_407_rubr	0.297964	0.492854	0.826087	0.702036	0.722222	0.306266	0.291219	65	20	NRPS
BGC_357_amar	BGC_374_pseu	0.23157	0.590485	0.88	0.76843	0.764706	0.220678	0.321429	33	1	NRPS

BGC_1	BGC_2	Raw distance	Squared similarity	Jaccard index	DSS index	Adjacency index	raw DSS non-anchor	raw DSS anchor	Non-anchor domains	Anchor domains	Network
BGC_034_neof	BGC_191_sihw	0.26764	0.536352	0.741935	0.73236	0.6	0.284885	0.138298	30	1	NRPS
BGC_064_amic	BGC_420_west	0.1973	0.644328	0.829268	0.8027	0.666667	0.18001	0.21459	48	12	NRPS
BGC_064_amic	BGC_308_alka	0.107115	0.797244	0.974359	0.892885	0.930233	0.094996	0.120335	48	11	NRPS
BGC_051_amic	BGC_439_lacu	0.244757	0.570392	0.586207	0.755243	0.4	0.272455	0.106267	20	1	NRPS
BGC_051_amic	BGC_493_terr	0.297408	0.493635	0.548387	0.702592	0.315789	0.32969	0.136	20	1	NRPS
BGC_307_alka	BGC_391_jing	0.247243	0.566643	0.789474	0.752757	0.604651	0.263601	0.10411	35	1	NRPS
BGC_064_amic	BGC_395_rubr	0.12088	0.772851	0.974359	0.87912	0.930233	0.104146	0.139136	48	11	NRPS
BGC_233_desu	BGC_308_alka	0.27508	0.525509	0.608696	0.72492	0.56	0.215799	0.302026	20	11	NRPS
BGC_395_rubr	BGC_420_west	0.194386	0.649013	0.85	0.805614	0.68	0.163951	0.224188	47	12	NRPS
BGC_147_nami	BGC_422_west	0.24324	0.572686	0.694444	0.75676	0.642857	0.12248	0.314275	40	17	NRPS
BGC_395_rubr	BGC_490_terr	0.280459	0.517739	0.780488	0.719541	0.62	0.245436	0.315482	44	11	NRPS
BGC_338_insu	BGC_418_west	0.268989	0.534377	0.53125	0.731011	0.470588	0.288848	0.179625	18	1	NRPS
BGC_058_amic	BGC_149_nami	0.124155	0.767104	0.942857	0.875845	0.842105	0.11525	0.224335	45	1	NRPS
BGC_444_lacu	BGC_483_terr	0.221779	0.605627	0.833333	0.778221	0.736842	0.088963	0.318373	32	11	NRPS
BGC_391_jing	BGC_517_para	0.273362	0.528003	0.714286	0.726638	0.5	0.288078	0.166667	29	1	NRPS
BGC_164_aich	BGC_425_jaco	0.260325	0.547119	0.857143	0.739675	0.733333	0.14861	0.330968	43	17	NRPS
BGC_141_nami	BGC_403_rubr	0.209256	0.625277	0.973684	0.790744	0.869565	0.103565	0.314946	44	11	NRPS
BGC_307_alka	BGC_418_west	0.118779	0.77655	0.939394	0.881221	0.833333	0.12536	0.062842	34	1	NRPS
BGC_064_amic	BGC_490_terr	0.277147	0.522517	0.804878	0.722853	0.607843	0.240148	0.314986	45	11	NRPS
BGC_250_hydr	BGC_295_phth	0.117595	0.778639	0.583333	0.882405	0.555556	0.118302	0.114058	20	1	NRPS
BGC_448_lacu	BGC_490_terr	0.146167	0.72903	0.857143	0.853833	0.780488	0.074018	0.204789	39	12	NRPS
BGC_150_nami	BGC_384_jing	0.229209	0.594119	0.870968	0.770791	0.783784	0.214671	0.256467	30	4	NRPS
BGC_338_insu	BGC_391_jing	0.267896	0.535977	0.457143	0.732104	0.378378	0.286022	0.19086	17	1	NRPS
BGC_233_desu	BGC_395_rubr	0.278651	0.520344	0.608696	0.721349	0.56	0.22102	0.304848	20	11	NRPS

BGC_1	BGC_2	Raw distance	Squared similarity	Jaccard index	DSS index	Adjacency index	raw DSS non-anchor	raw DSS anchor	Non-anchor domains	Anchor domains	Network
BGC_149_nami	BGC_401_rubr	0.105459	0.800204	0.911765	0.894541	0.725	0.105293	0.10728	44	1	NRPS
BGC_055_amic	BGC_386_jing	0.135111	0.748034	0.818182	0.870083	0.636364	0.129917	0	9	0	Others
BGC_345_zhao	BGC_464_aspl	0.260653	0.546633	1	0.739533	0.6	0.260467	0	13	0	Others
BGC_105_otit	BGC_464_aspl	0.221978	0.605318	1	0.771156	1	0.228844	0	14	0	Others
BGC_117_sput	BGC_423_jaco	0.018749	0.962853	1	0.980671	1	0.019329	0	14	0	Others
BGC_411_west	BGC_464_aspl	0.224152	0.601941	1	0.768916	1	0.231084	0	14	0	Others
BGC_340_insu	BGC_480_hank	0.290913	0.502805	0.916667	0.703526	0.875	0.296474	0	25	0	Others
BGC_309_alka	BGC_489_terr	0.296394	0.495061	0.826087	0.70283	0.68	0.29717	0	24	0	Others
BGC_314_alka	BGC_407_rubr	0.123204	0.768772	0.612245	0.885231	0.6	0.109203	0.118109	48	20	Others
BGC_228_desu	BGC_471_humi	0.29817	0.492566	0.857143	0.704391	0.5	0.295609	0	13	0	Others
BGC_228_desu	BGC_296_phth	0.296931	0.494305	0.857143	0.705667	0.5	0.294333	0	13	0	Others
BGC_003_bron	BGC_404_rubr	0.214543	0.616942	0.928571	0.783976	0.785714	0.216024	0	14	0	Others
BGC_017_poly	BGC_122_rhiz	0.196064	0.646313	0.928571	0.803027	0.785714	0.196973	0	13	0	Others
BGC_047_shan	BGC_277_spum	0.190713	0.654946	1	0.803389	1	0.196611	0	13	0	Others
BGC_296_phth	BGC_464_aspl	0.245934	0.568615	1	0.754707	0.6	0.245293	0	13	0	Others
BGC_187_sihw	BGC_510_para	0.24412	0.571354	1	0.756577	0.6	0.243423	0	13	0	Others
BGC_303_alka	BGC_402_rubr	0.16215	0.701993	0.761905	0.84076	0.734694	0.211095	0.052936	41	5	Others
BGC_327_insu	BGC_507_hank	0.114827	0.783531	1	0.881621	1	0.118379	0	14	0	Others
BGC_017_poly	BGC_471_humi	0.252434	0.558854	1	0.748006	0.6	0.251994	0	13	0	Others
BGC_464_aspl	BGC_507_hank	0.205397	0.631394	0.928571	0.793405	0.785714	0.206595	0	13	0	Others
BGC_423_jaco	BGC_447_lacu	0.177147	0.677087	1	0.817374	1	0.182626	0	14	0	Others
BGC_105_otit	BGC_311_alka	0.243833	0.571789	0.818182	0.757998	0.636364	0.242002	0	9	0	Others
BGC_314_alka	BGC_463_para	0.29928	0.491009	0.5625	0.705915	0.517857	0.283323	0.300811	50	20	Others
BGC_228_desu	BGC_386_jing	0.266136	0.538557	0.727273	0.740473	0.416667	0.259527	0	8	0	Others

BGC_1	BGC_2	Raw distance	Squared similarity	Jaccard index	DSS index	Adjacency index	raw DSS non-anchor	raw DSS anchor	Non-anchor domains	Anchor domains	Network
BGC_407_rubr	BGC_463_para	0.271323	0.53097	0.851064	0.726976	0.75	0.235135	0.302388	62	20	Others
BGC_017_poly	BGC_277_spum	0.243226	0.572707	1	0.757499	0.6	0.242501	0	13	0	Others
BGC_003_bron	BGC_203_soli	0.247703	0.565951	0.846154	0.755059	0.571429	0.244941	0	12	0	Others
BGC_251_hydr	BGC_423_jaco	0.248223	0.565169	1	0.752347	0.6	0.247653	0	13	0	Others
BGC_080_effu	BGC_122_rhiz	0.280469	0.517725	0.923077	0.713368	0.916667	0.286632	0	13	0	Others
BGC_065_amic	BGC_303_alka	0.247949	0.56558	0.767442	0.752844	0.705882	0.228967	0.284443	41	5	Others
BGC_105_otit	BGC_117_sput	0.057368	0.888555	1	0.940858	1	0.059142	0	14	0	Others
BGC_507_hank	BGC_510_para	0.219471	0.609225	0.928571	0.778896	0.785714	0.221104	0	14	0	Others
BGC_199_soli	BGC_480_hank	0.299194	0.491129	0.92	0.697532	0.75	0.302468	0	26	0	Others
BGC_017_poly	BGC_031_neof	0.244573	0.57067	1	0.75611	0.6	0.24389	0	13	0	Others
BGC_117_sput	BGC_471_humi	0.244914	0.570154	1	0.755758	0.6	0.244242	0	13	0	Others
BGC_404_rubr	BGC_423_jaco	0.189468	0.656963	1	0.804673	1	0.195327	0	14	0	Others
BGC_042_shan	BGC_175_hirs	0.274529	0.526308	0.888889	0.725623	0.636364	0.274377	0	18	0	Others
BGC_117_sput	BGC_277_spum	0.232147	0.589599	1	0.768921	0.6	0.231079	0	13	0	Others
BGC_406_rubr	BGC_409_west	0.252199	0.559207	0.911111	0.744866	0.808511	0.233967	0.310698	42	4	Others
BGC_187_sihw	BGC_507_hank	0.246259	0.568126	0.928571	0.757777	0.470588	0.242223	0	13	0	Others
BGC_165_aich	BGC_404_rubr	0.187796	0.659675	1	0.806396	1	0.193604	0	14	0	Others
BGC_445_lacu	BGC_518_para	0.231054	0.591277	0.861111	0.768162	0.76087	0.228312	0.237127	36	6	Others
BGC_277_spum	BGC_319_iter	0.276529	0.52341	0.888889	0.729185	0.363636	0.270815	0	8	0	Others
BGC_105_otit	BGC_327_insu	0.170256	0.688476	0.928571	0.829633	0.785714	0.170367	0	13	0	Others
BGC_047_shan	BGC_146_nami	0.263196	0.54288	1	0.736911	0.6	0.263089	0	13	0	Others
BGC_117_sput	BGC_146_nami	0.180705	0.671244	1	0.813706	1	0.186294	0	14	0	Others
BGC_055_amic	BGC_203_soli	0.192627	0.651851	0.923077	0.806967	0.769231	0.193033	0	12	0	Others
BGC_313_alka	BGC_406_rubr	0.195718	0.64687	0.933333	0.802426	0.829787	0.159566	0.297344	42	4	Others

BGC_1	BGC_2	Raw distance	Squared similarity	Jaccard index	DSS index	Adjacency index	raw DSS non-anchor	raw DSS anchor	Non-anchor domains	Anchor domains	Network
BGC_150_nami	BGC_303_alka	0.076297	0.853228	0.966667	0.923455	0.914286	0.058264	0.110822	30	4	Others
BGC_055_amic	BGC_277_spum	0.235856	0.583916	1	0.765097	0.6	0.234903	0	13	0	Others
BGC_404_rubr	BGC_507_hank	0.175852	0.67922	0.928571	0.823864	0.785714	0.176136	0	13	0	Others
BGC_309_alka	BGC_390_jing	0.291367	0.502161	0.826087	0.708012	0.68	0.291988	0	24	0	Others
BGC_146_nami	BGC_404_rubr	0.036878	0.927604	1	0.961981	1	0.038019	0	14	0	Others
BGC_080_effu	BGC_484_terr	0.285521	0.51048	0.727273	0.720487	0.416667	0.279513	0	8	0	Others
BGC_165_aich	BGC_464_aspl	0.219739	0.608807	1	0.773465	1	0.226535	0	14	0	Others
BGC_411_west	BGC_447_lacu	0.124506	0.76649	1	0.871644	1	0.128356	0	14	0	Others
BGC_423_jaco	BGC_484_terr	0.22927	0.594025	0.818182	0.773011	0.636364	0.226989	0	9	0	Others
BGC_105_otit	BGC_507_hank	0.156896	0.710825	0.928571	0.843406	0.785714	0.156594	0	13	0	Others
BGC_165_aich	BGC_484_terr	0.241761	0.574927	0.818182	0.760134	0.636364	0.239866	0	9	0	Others
BGC_165_aich	BGC_371_pseu	0.271418	0.530832	0.785714	0.732019	0.533333	0.267981	0	12	0	Others
BGC_143_nami	BGC_394_rubr	0.062532	0.878847	1	0.935534	1	0.064466	0	22	0	Others
BGC_187_sihw	BGC_203_soli	0.286198	0.509513	0.916667	0.716119	0.5	0.283881	0	11	0	Others
BGC_386_jing	BGC_484_terr	0.15618	0.712031	1	0.838989	1	0.161011	0	11	0	Others
BGC_085_effu	BGC_505_aspl	0.201716	0.637258	0.772727	0.803839	0.541667	0.196161	0	18	0	Others
BGC_105_otit	BGC_471_humi	0.248794	0.564311	1	0.751759	0.6	0.248241	0	13	0	Others
BGC_003_bron	BGC_423_jaco	0.218636	0.61053	0.928571	0.779757	0.785714	0.220243	0	14	0	Others
BGC_122_rhiz	BGC_404_rubr	0.247017	0.566983	0.928571	0.750498	0.785714	0.249502	0	14	0	Others
BGC_404_rubr	BGC_510_para	0.113718	0.785495	1	0.882765	1	0.117235	0	14	0	Others
BGC_105_otit	BGC_277_spum	0.241696	0.575025	1	0.759076	0.6	0.240924	0	13	0	Others
BGC_017_poly	BGC_371_pseu	0.262632	0.543712	0.785714	0.741077	0.533333	0.258923	0	12	0	Others
BGC_065_amic	BGC_414_west	0.264981	0.540253	0.767442	0.73569	0.686275	0.22958	0.333771	40	5	Others
BGC_222_mala	BGC_251_hydr	0.213636	0.618368	1	0.779756	1	0.220244	0	13	0	Others

BGC_1	BGC_2	Raw distance	Squared similarity	Jaccard index	DSS index	Adjacency index	raw DSS non-anchor	raw DSS anchor	Non-anchor domains	Anchor domains	Network
BGC_080_effu	BGC_404_rubr	0.219395	0.609345	0.928571	0.778975	0.785714	0.221025	0	13	0	Others
BGC_055_amic	BGC_327_insu	0.164931	0.69734	0.928571	0.835123	0.785714	0.164877	0	13	0	Others
BGC_314_alka	BGC_412_west	0.156046	0.712258	0.666667	0.849701	0.653846	0.121684	0.168183	50	20	Others
BGC_140_nami	BGC_409_west	0.225693	0.599552	0.840909	0.77541	0.6875	0.263431	0.125059	41	4	Others
BGC_464_aspl	BGC_484_terr	0.294192	0.498165	0.818182	0.706081	0.636364	0.293919	0	9	0	Others
BGC_117_sput	BGC_203_soli	0.203863	0.633834	0.923077	0.795383	0.769231	0.204617	0	12	0	Others
BGC_229_desu	BGC_449_lacu	0.265266	0.539834	0.8	0.733174	0.777778	0.266826	0	9	0	Others
BGC_105_otit	BGC_222_mala	0.246795	0.567317	1	0.753819	0.6	0.246181	0	13	0	Others
BGC_003_bron	BGC_371_pseu	0.270402	0.532314	0.857143	0.728599	0.714286	0.271401	0	13	0	Others
BGC_031_neof	BGC_251_hydr	0.142624	0.735093	1	0.852965	1	0.147035	0	13	0	Others
BGC_031_neof	BGC_296_phth	0.126726	0.762608	1	0.869355	1	0.130645	0	13	0	Others
BGC_146_nami	BGC_251_hydr	0.262781	0.543492	1	0.737339	0.6	0.262661	0	13	0	Others
BGC_017_poly	BGC_423_jaco	0.219837	0.608654	0.928571	0.775686	0.923077	0.224314	0	14	0	Others
BGC_146_nami	BGC_327_insu	0.177326	0.676793	0.928571	0.822345	0.785714	0.177655	0	13	0	Others
BGC_117_sput	BGC_510_para	0.172192	0.685265	1	0.822482	1	0.177518	0	14	0	Others
BGC_080_effu	BGC_447_lacu	0.21226	0.620534	0.928571	0.78633	0.785714	0.21367	0	13	0	Others
BGC_150_nami	BGC_414_west	0.236219	0.583361	0.965517	0.758043	0.941176	0.115033	0.432342	30	5	Others
BGC_228_desu	BGC_447_lacu	0.196466	0.645667	0.928571	0.802612	0.785714	0.197388	0	13	0	Others
BGC_222_mala	BGC_423_jaco	0.238679	0.57961	1	0.762187	0.6	0.237813	0	13	0	Others
BGC_447_lacu	BGC_464_aspl	0.220503	0.607616	1	0.772677	1	0.227323	0	14	0	Others
BGC_165_aich	BGC_345_zhao	0.244898	0.570179	1	0.755775	0.6	0.244225	0	13	0	Others
BGC_117_sput	BGC_165_aich	0.057819	0.887705	1	0.940393	1	0.059607	0	14	0	Others
BGC_296_phth	BGC_345_zhao	0.221948	0.605366	1	0.771188	1	0.228812	0	13	0	Others
BGC_031_neof	BGC_047_shan	0.157972	0.709012	1	0.837143	1	0.162857	0	13	0	Others

BGC_1	BGC_2	Raw distance	Squared similarity	Jaccard index	DSS index	Adjacency index	raw DSS non-anchor	raw DSS anchor	Non-anchor domains	Anchor domains	Network
BGC_471_humi	BGC_510_para	0.250644	0.561534	1	0.749851	0.6	0.250149	0	13	0	Others
BGC_203_soli	BGC_411_west	0.271477	0.530746	0.923077	0.725678	0.769231	0.274322	0	13	0	Others
BGC_117_sput	BGC_411_west	0.183816	0.666157	1	0.810499	1	0.189501	0	14	0	Others
BGC_055_amic	BGC_447_lacu	0.110642	0.790958	1	0.885936	1	0.114064	0	14	0	Others
BGC_052_amic	BGC_421_west	0.163745	0.699322	0.857143	0.840395	0.625	0.159605	0	21	0	Others
BGC_404_rubr	BGC_484_terr	0.171916	0.685723	0.818182	0.832139	0.636364	0.167861	0	9	0	Others
BGC_065_amic	BGC_150_nami	0.266002	0.538754	0.852941	0.731517	0.794872	0.250386	0.30807	35	4	Others
BGC_203_soli	BGC_447_lacu	0.252803	0.558304	0.923077	0.74493	0.769231	0.25507	0	13	0	Others
BGC_017_poly	BGC_055_amic	0.23599	0.583712	0.928571	0.759034	0.923077	0.240966	0	14	0	Others
BGC_003_bron	BGC_122_rhiz	0.207461	0.628118	1	0.786123	1	0.213877	0	14	0	Others
BGC_296_phth	BGC_404_rubr	0.264191	0.541416	1	0.735886	0.6	0.264114	0	13	0	Others
BGC_313_alka	BGC_409_west	0.205434	0.631335	0.888889	0.795543	0.7	0.238591	0.114853	42	4	Others
BGC_055_amic	BGC_411_west	0.068639	0.867433	1	0.929238	1	0.070762	0	14	0	Others
BGC_165_aich	BGC_471_humi	0.241073	0.575971	1	0.759719	0.6	0.240281	0	13	0	Others
BGC_384_jing	BGC_402_rubr	0.260331	0.54711	0.785714	0.7396	0.72	0.265927	0.249623	39	5	Others
BGC_222_mala	BGC_471_humi	0.140655	0.738474	1	0.854995	1	0.145005	0	13	0	Others
BGC_047_shan	BGC_510_para	0.247754	0.565874	1	0.752831	0.6	0.247169	0	13	0	Others
BGC_057_amic	BGC_152_nami	0.176252	0.678561	0.740741	0.828467	0.636364	0.184935	0.162597	32	12	Others
BGC_423_jaco	BGC_507_hank	0.157236	0.710251	0.928571	0.843056	0.785714	0.156944	0	13	0	Others
BGC_105_otit	BGC_228_desu	0.238248	0.580266	0.928571	0.759538	0.785714	0.240462	0	14	0	Others
BGC_105_otit	BGC_371_pseu	0.272148	0.529769	0.785714	0.731267	0.533333	0.268733	0	12	0	Others
BGC_105_otit	BGC_386_jing	0.246152	0.568286	0.818182	0.755607	0.636364	0.244393	0	9	0	Others
BGC_327_insu	BGC_404_rubr	0.22935	0.593902	0.928571	0.768711	0.785714	0.231289	0	14	0	Others
BGC_003_bron	BGC_510_para	0.211458	0.621798	0.928571	0.787157	0.785714	0.212843	0	14	0	Others

BGC_1	BGC_2	Raw distance	Squared similarity	Jaccard index	DSS index	Adjacency index	raw DSS non-anchor	raw DSS anchor	Non-anchor domains	Anchor domains	Network
BGC_146_nami	BGC_484_terr	0.15466	0.7146	0.818182	0.849929	0.636364	0.150071	0	9	0	Others
BGC_311_alka	BGC_484_terr	0.170467	0.688126	1	0.824261	1	0.175739	0	11	0	Others
BGC_031_neof	BGC_447_lacu	0.252507	0.558746	1	0.747931	0.6	0.252069	0	13	0	Others
BGC_031_neof	BGC_345_zhao	0.213064	0.619268	1	0.780346	1	0.219654	0	13	0	Others
BGC_105_otit	BGC_510_para	0.176772	0.677705	1	0.817761	1	0.182239	0	14	0	Others
BGC_228_desu	BGC_327_insu	0.259254	0.548705	0.8	0.74381	0.5625	0.25619	0	13	0	Others
BGC_146_nami	BGC_464_aspl	0.216715	0.613535	1	0.776582	1	0.223418	0	14	0	Others
BGC_146_nami	BGC_345_zhao	0.267746	0.536195	1	0.73222	0.6	0.26778	0	13	0	Others
BGC_003_bron	BGC_228_desu	0.297208	0.493917	0.8	0.704683	0.5625	0.295317	0	14	0	Others
BGC_187_sihw	BGC_411_west	0.259653	0.548114	1	0.740564	0.6	0.259436	0	13	0	Others
BGC_160_aich	BGC_430_jaco	0.12165	0.771499	1	0.874588	1	0.125412	0	22	0	Others
BGC_031_neof	BGC_411_west	0.25337	0.557457	1	0.747041	0.6	0.252959	0	13	0	Others
BGC_187_sihw	BGC_464_aspl	0.249067	0.5639	1	0.751477	0.6	0.248523	0	13	0	Others
BGC_187_sihw	BGC_447_lacu	0.252254	0.559124	1	0.748192	0.6	0.251808	0	13	0	Others
BGC_105_otit	BGC_122_rhiz	0.18973	0.656538	0.928571	0.809557	0.785714	0.190443	0	13	0	Others
BGC_105_otit	BGC_423_jaco	0.055215	0.892618	1	0.943077	1	0.056923	0	14	0	Others
BGC_047_shan	BGC_404_rubr	0.26968	0.533367	1	0.730227	0.6	0.269773	0	13	0	Others
BGC_411_west	BGC_423_jaco	0.185445	0.6635	1	0.80882	1	0.19118	0	14	0	Others
BGC_414_west	BGC_518_para	0.248134	0.565303	0.846154	0.749364	0.826087	0.26233	0.227248	40	5	Others
BGC_003_bron	BGC_507_hank	0.165074	0.697102	1	0.829821	1	0.170179	0	14	0	Others
BGC_203_soli	BGC_327_insu	0.251449	0.560328	0.846154	0.751196	0.571429	0.248804	0	12	0	Others
BGC_311_alka	BGC_404_rubr	0.240307	0.577133	0.818182	0.761633	0.636364	0.238367	0	11	0	Others
BGC_277_spum	BGC_296_phth	0.202392	0.636178	1	0.791348	1	0.208652	0	13	0	Others
BGC_117_sput	BGC_187_sihw	0.249292	0.563562	1	0.751245	0.6	0.248755	0	13	0	Others

BGC_1	BGC_2	Raw distance	Squared similarity	Jaccard index	DSS index	Adjacency index	raw DSS non-anchor	raw DSS anchor	Non-anchor domains	Anchor domains	Network
BGC_080_effu	BGC_311_alka	0.292969	0.499893	0.727273	0.71281	0.416667	0.28719	0	8	0	Others
BGC_055_amic	BGC_228_desu	0.237104	0.582011	0.928571	0.760718	0.785714	0.239282	0	14	0	Others
BGC_345_zhao	BGC_507_hank	0.257489	0.551323	0.928571	0.7462	0.470588	0.2538	0	13	0	Others
BGC_228_desu	BGC_277_spum	0.288252	0.506585	0.857143	0.714615	0.5	0.285385	0	13	0	Others
BGC_047_shan	BGC_117_sput	0.252817	0.558282	1	0.747611	0.6	0.252389	0	13	0	Others
BGC_447_lacu	BGC_471_humi	0.258321	0.550088	1	0.741937	0.6	0.258063	0	13	0	Others
BGC_345_zhao	BGC_471_humi	0.149367	0.723577	1	0.846014	1	0.153986	0	13	0	Others
BGC_165_aich	BGC_296_phth	0.239542	0.578297	1	0.761297	0.6	0.238703	0	13	0	Others
BGC_146_nami	BGC_296_phth	0.252593	0.558617	1	0.747842	0.6	0.252158	0	13	0	Others
BGC_065_amic	BGC_402_rubr	0.183826	0.66614	0.95	0.814075	0.851064	0.141589	0.276813	41	5	Others
BGC_411_west	BGC_471_humi	0.26399	0.541711	1	0.736093	0.6	0.263907	0	13	0	Others
BGC_386_jing	BGC_447_lacu	0.281227	0.516634	0.818182	0.719447	0.636364	0.280553	0	11	0	Others
BGC_146_nami	BGC_371_pseu	0.274549	0.52628	0.785714	0.728791	0.533333	0.271209	0	12	0	Others
BGC_117_sput	BGC_386_jing	0.241826	0.574828	0.818182	0.760067	0.636364	0.239933	0	9	0	Others
BGC_031_neof	BGC_464_aspl	0.251895	0.559661	1	0.748562	0.6	0.251438	0	13	0	Others
BGC_327_insu	BGC_355_amar	0.28187	0.515711	0.857143	0.716776	0.714286	0.283224	0	13	0	Others
BGC_327_insu	BGC_510_para	0.240591	0.576702	0.928571	0.757123	0.785714	0.242877	0	14	0	Others
BGC_017_poly	BGC_447_lacu	0.244203	0.571229	0.928571	0.750567	0.923077	0.249433	0	14	0	Others
BGC_122_rhiz	BGC_507_hank	0.176445	0.678242	1	0.818098	1	0.181902	0	14	0	Others
BGC_080_effu	BGC_423_jaco	0.207593	0.62791	0.928571	0.791142	0.785714	0.208858	0	13	0	Others
BGC_389_jing	BGC_407_rubr	0.29632	0.495165	0.826087	0.702036	0.722222	0.306266	0.291219	65	20	Others
BGC_143_nami	BGC_512_para	0.193595	0.650289	0.875	0.808579	0.666667	0.191421	0	22	0	Others
BGC_017_poly	BGC_411_west	0.250562	0.561657	0.928571	0.744011	0.923077	0.255989	0	14	0	Others
BGC_222_mala	BGC_464_aspl	0.262872	0.543358	1	0.737246	0.6	0.262754	0	13	0	Others

BGC_1	BGC_2	Raw distance	Squared similarity	Jaccard index	DSS index	Adjacency index	raw DSS non-anchor	raw DSS anchor	Non-anchor domains	Anchor domains	Network
BGC_003_bron	BGC_117_sput	0.219348	0.609417	0.928571	0.779022	0.785714	0.220978	0	14	0	Others
BGC_052_amic	BGC_390_jing	0.233364	0.58773	0.904762	0.764149	0.818182	0.235851	0	22	0	Others
BGC_003_bron	BGC_311_alka	0.281975	0.515559	0.727273	0.724143	0.416667	0.275857	0	9	0	Others
BGC_421_west	BGC_512_para	0.236829	0.58243	0.826087	0.765569	0.615385	0.234431	0	21	0	Others
BGC_017_poly	BGC_165_aich	0.219041	0.609897	0.928571	0.776507	0.923077	0.223493	0	14	0	Others
BGC_031_neof	BGC_055_amic	0.245578	0.569152	1	0.755074	0.6	0.244926	0	13	0	Others
BGC_357_amar	BGC_374_pseu	0.230528	0.592086	0.88	0.76843	0.764706	0.220678	0.321429	33	1	Others
BGC_355_amar	BGC_371_pseu	0.095538	0.818051	1	0.901507	1	0.098493	0	13	0	Others
BGC_055_amic	BGC_296_phth	0.246038	0.568458	1	0.7546	0.6	0.2454	0	13	0	Others
BGC_055_amic	BGC_471_humi	0.248857	0.564216	1	0.751694	0.6	0.248306	0	13	0	Others
BGC_165_aich	BGC_411_west	0.18734	0.660417	1	0.806866	1	0.193134	0	14	0	Others
BGC_228_desu	BGC_507_hank	0.24829	0.565068	0.8	0.755114	0.5625	0.244886	0	13	0	Others
BGC_003_bron	BGC_146_nami	0.203219	0.63486	0.928571	0.79565	0.785714	0.20435	0	14	0	Others
BGC_251_hydr	BGC_510_para	0.251078	0.560884	1	0.749404	0.6	0.250596	0	13	0	Others
BGC_394_rubr	BGC_512_para	0.228561	0.595117	0.84	0.773383	0.642857	0.226617	0	23	0	Others
BGC_165_aich	BGC_447_lacu	0.184082	0.665722	1	0.810225	1	0.189775	0	14	0	Others
BGC_085_effu	BGC_229_desu	0.266106	0.5386	0.611111	0.739982	0.5	0.260018	0	11	0	Others
BGC_165_aich	BGC_228_desu	0.240886	0.576254	0.928571	0.756819	0.785714	0.243181	0	14	0	Others
BGC_122_rhiz	BGC_203_soli	0.285572	0.510407	0.846154	0.716018	0.571429	0.283982	0	12	0	Others
BGC_117_sput	BGC_507_hank	0.15734	0.710075	0.928571	0.842948	0.785714	0.157052	0	13	0	Others
BGC_390_jing	BGC_394_rubr	0.156905	0.710809	0.952381	0.841545	0.863636	0.158455	0	21	0	Others
BGC_384_jing	BGC_414_west	0.288953	0.505588	0.775	0.711162	0.673469	0.197209	0.449187	35	5	Others
BGC_080_effu	BGC_203_soli	0.244831	0.57028	0.846154	0.758019	0.571429	0.241981	0	11	0	Others
BGC_277_spum	BGC_510_para	0.232563	0.58896	1	0.768492	0.6	0.231508	0	13	0	Others

BGC_1	BGC_2	Raw distance	Squared similarity	Jaccard index	DSS index	Adjacency index	raw DSS non-anchor	raw DSS anchor	Non-anchor domains	Anchor domains	Network
BGC_031_neof	BGC_165_aich	0.235395	0.58462	1	0.765572	0.6	0.234428	0	13	0	Others
BGC_055_amic	BGC_345_zhao	0.260249	0.547232	1	0.73995	0.6	0.26005	0	13	0	Others
BGC_222_mala	BGC_277_spum	0.087109	0.83337	1	0.910197	1	0.089803	0	13	0	Others
BGC_228_desu	BGC_510_para	0.18949	0.656927	0.928571	0.809804	0.785714	0.190196	0	13	0	Others
BGC_017_poly	BGC_464_aspl	0.268059	0.535738	0.928571	0.725973	0.923077	0.274027	0	14	0	Others
BGC_251_hydr	BGC_507_hank	0.255082	0.554902	0.928571	0.748681	0.470588	0.251319	0	13	0	Others
BGC_126_rhiz	BGC_449_lacu	0.283569	0.513274	1	0.709247	0.923077	0.290753	0	14	0	Others
BGC_187_sihw	BGC_423_jaco	0.248875	0.564189	1	0.751675	0.6	0.248325	0	13	0	Others
BGC_055_amic	BGC_146_nami	0.054594	0.893793	1	0.943718	1	0.056282	0	14	0	Others
BGC_345_zhao	BGC_510_para	0.263585	0.542307	1	0.736511	0.6	0.263489	0	13	0	Others
BGC_017_poly	BGC_187_sihw	0.251501	0.560251	1	0.748968	0.6	0.251032	0	13	0	Others
BGC_031_neof	BGC_507_hank	0.237559	0.581316	0.928571	0.766746	0.470588	0.233255	0	13	0	Others
BGC_052_amic	BGC_309_alka	0.13629	0.745994	0.833333	0.867557	0.692308	0.132443	0	22	0	Others
BGC_146_nami	BGC_507_hank	0.164184	0.698589	0.928571	0.835893	0.785714	0.164107	0	13	0	Others
BGC_031_neof	BGC_471_humi	0.199247	0.641206	1	0.794591	1	0.205409	0	13	0	Others
BGC_165_aich	BGC_327_insu	0.170144	0.688661	0.928571	0.829749	0.785714	0.170251	0	13	0	Others
BGC_055_amic	BGC_105_otit	0.172489	0.684774	1	0.822176	1	0.177824	0	14	0	Others
BGC_080_effu	BGC_464_aspl	0.119168	0.775865	0.928571	0.882301	0.785714	0.117699	0	13	0	Others
BGC_126_rhiz	BGC_143_nami	0.275746	0.524544	0.772727	0.724344	0.695652	0.275656	0	19	0	Others
BGC_390_jing	BGC_449_lacu	0.11096	0.790392	1	0.885608	1	0.114392	0	14	0	Others
BGC_052_amic	BGC_512_para	0.245552	0.569192	0.869565	0.756128	0.615385	0.243872	0	22	0	Others
BGC_449_lacu	BGC_512_para	0.215435	0.615543	1	0.779489	0.923077	0.220512	0	14	0	Others
BGC_003_bron	BGC_031_neof	0.299955	0.490063	0.928571	0.70242	0.470588	0.29758	0	14	0	Others
BGC_047_shan	BGC_464_aspl	0.255277	0.554612	1	0.745075	0.6	0.254925	0	13	0	Others

BGC_1	BGC_2	Raw distance	Squared similarity	Jaccard index	DSS index	Adjacency index	raw DSS non-anchor	raw DSS anchor	Non-anchor domains	Anchor domains	Network
BGC_047_shan	BGC_319_iter	0.29614	0.495419	0.888889	0.708967	0.363636	0.291033	0	8	0	Others
BGC_055_amic	BGC_117_sput	0.174023	0.682238	1	0.820595	1	0.179405	0	14	0	Others
BGC_105_otit	BGC_146_nami	0.181853	0.669365	1	0.812523	1	0.187477	0	14	0	Others
BGC_003_bron	BGC_447_lacu	0.207321	0.628339	0.928571	0.791421	0.785714	0.208579	0	14	0	Others
BGC_017_poly	BGC_203_soli	0.276399	0.523599	0.846154	0.722983	0.692308	0.277017	0	12	0	Others
BGC_031_neof	BGC_105_otit	0.245264	0.569626	1	0.755398	0.6	0.244602	0	13	0	Others
BGC_055_amic	BGC_371_pseu	0.267964	0.535877	0.785714	0.73558	0.533333	0.26442	0	12	0	Others
BGC_355_amar	BGC_507_hank	0.271828	0.530235	0.857143	0.727129	0.714286	0.272871	0	13	0	Others
BGC_042_shan	BGC_185_sihw	0.279345	0.519344	0.84	0.720775	0.655172	0.279225	0	24	0	Others
BGC_047_shan	BGC_187_sihw	0.163604	0.699559	1	0.831336	1	0.168664	0	13	0	Others
BGC_003_bron	BGC_165_aich	0.215233	0.615859	0.928571	0.783265	0.785714	0.216735	0	14	0	Others
BGC_394_rubr	BGC_421_west	0.129285	0.758144	0.904762	0.873077	0.73913	0.126923	0	21	0	Others
BGC_095_otit	BGC_114_sput	0.157802	0.709297	1	0.837317	1	0.162683	0	22	0	Others
BGC_017_poly	BGC_117_sput	0.21932	0.609462	0.928571	0.77622	0.923077	0.22378	0	14	0	Others
BGC_277_spum	BGC_404_rubr	0.257683	0.551034	1	0.742595	0.6	0.257405	0	13	0	Others
BGC_105_otit	BGC_484_terr	0.24223	0.574216	0.818182	0.759651	0.636364	0.240349	0	9	0	Others
BGC_394_rubr	BGC_449_lacu	0.126801	0.762477	1	0.869277	1	0.130722	0	14	0	Others
BGC_165_aich	BGC_277_spum	0.233756	0.58713	1	0.767262	0.6	0.232738	0	13	0	Others
BGC_165_aich	BGC_203_soli	0.206988	0.628868	0.923077	0.792161	0.769231	0.207839	0	12	0	Others
BGC_117_sput	BGC_371_pseu	0.268784	0.534676	0.785714	0.734734	0.533333	0.265266	0	12	0	Others
BGC_117_sput	BGC_296_phth	0.243198	0.572749	1	0.757528	0.6	0.242472	0	13	0	Others
BGC_047_shan	BGC_122_rhiz	0.277959	0.521343	0.928571	0.725096	0.470588	0.274904	0	13	0	Others
BGC_122_rhiz	BGC_411_west	0.247601	0.566104	0.928571	0.749896	0.785714	0.250104	0	14	0	Others
BGC_052_amic	BGC_394_rubr	0.124918	0.765769	0.952381	0.874521	0.863636	0.125479	0	22	0	Others

BGC_1	BGC_2	Raw distance	Squared similarity	Jaccard index	DSS index	Adjacency index	raw DSS non-anchor	raw DSS anchor	Non-anchor domains	Anchor domains	Network
BGC_228_desu	BGC_423_jaco	0.188955	0.657793	0.928571	0.810355	0.785714	0.189645	0	13	0	Others
BGC_143_nami	BGC_480_hank	0.298072	0.492703	0.869565	0.699827	0.72	0.300173	0	22	0	Others
BGC_222_mala	BGC_447_lacu	0.256221	0.553208	1	0.744103	0.6	0.255897	0	13	0	Others
BGC_017_poly	BGC_507_hank	0.173403	0.683263	0.928571	0.826389	0.785714	0.173611	0	13	0	Others
BGC_445_lacu	BGC_482_terr	0.135825	0.746799	0.971429	0.861251	0.952381	0.061395	0.261227	38	6	Others
BGC_080_effu	BGC_411_west	0.210837	0.622779	0.928571	0.787797	0.785714	0.212203	0	13	0	Others
BGC_423_jaco	BGC_510_para	0.171963	0.685645	1	0.822719	1	0.177281	0	14	0	Others
BGC_146_nami	BGC_187_sihw	0.258757	0.549441	1	0.741487	0.6	0.258513	0	13	0	Others
BGC_404_rubr	BGC_464_aspl	0.225322	0.600126	1	0.767709	1	0.232291	0	14	0	Others
BGC_303_alka	BGC_414_west	0.223222	0.603384	0.846154	0.776286	0.765957	0.105712	0.436118	36	5	Others
BGC_345_zhao	BGC_404_rubr	0.280603	0.517533	1	0.718966	0.6	0.281034	0	13	0	Others
BGC_303_alka	BGC_384_jing	0.278347	0.520782	0.820513	0.71972	0.765957	0.297928	0.244101	41	5	Others
BGC_251_hydr	BGC_296_phth	0.110834	0.790616	1	0.885738	1	0.114262	0	13	0	Others
BGC_122_rhiz	BGC_510_para	0.246371	0.567957	0.928571	0.751164	0.785714	0.248836	0	14	0	Others
BGC_384_jing	BGC_518_para	0.299102	0.491258	0.861111	0.698458	0.73913	0.228254	0.411475	36	6	Others
BGC_031_neof	BGC_228_desu	0.289301	0.505092	0.857143	0.713533	0.5	0.286467	0	13	0	Others
BGC_187_sihw	BGC_277_spum	0.195817	0.64671	1	0.798127	1	0.201873	0	13	0	Others
BGC_414_west	BGC_445_lacu	0.27498	0.525654	0.780488	0.724964	0.7	0.297314	0.229366	41	5	Others
BGC_143_nami	BGC_449_lacu	0.123995	0.767385	1	0.87217	1	0.12783	0	14	0	Others
BGC_203_soli	BGC_423_jaco	0.264541	0.540899	0.923077	0.732828	0.769231	0.267172	0	13	0	Others
BGC_017_poly	BGC_047_shan	0.258293	0.550129	1	0.741966	0.6	0.258034	0	13	0	Others
BGC_421_west	BGC_449_lacu	0.13723	0.744373	0.923077	0.863737	0.785714	0.136263	0	13	0	Others
BGC_047_shan	BGC_345_zhao	0.207853	0.627496	1	0.785718	1	0.214282	0	13	0	Others
BGC_187_sihw	BGC_471_humi	0.200681	0.63891	1	0.793112	1	0.206888	0	13	0	Others

BGC_1	BGC_2	Raw distance	Squared similarity	Jaccard index	DSS index	Adjacency index	raw DSS non-anchor	raw DSS anchor	Non-anchor domains	Anchor domains	Network
BGC_080_effu	BGC_386_jing	0.289177	0.50527	0.727273	0.716719	0.416667	0.283281	0	8	0	Others
BGC_055_amic	BGC_484_terr	0.161548	0.703001	0.818182	0.842827	0.636364	0.157173	0	9	0	Others
BGC_423_jaco	BGC_471_humi	0.247599	0.566108	1	0.752991	0.6	0.247009	0	13	0	Others
BGC_146_nami	BGC_222_mala	0.25902	0.549051	1	0.741216	0.6	0.258784	0	13	0	Others
BGC_228_desu	BGC_311_alka	0.258872	0.54927	0.727273	0.74796	0.416667	0.25204	0	8	0	Others
BGC_407_rubr	BGC_487_terr	0.299778	0.490311	0.884615	0.696534	0.786885	0.231368	0.361831	68	21	Others
BGC_047_shan	BGC_411_west	0.27445	0.526422	1	0.725309	0.6	0.274691	0	13	0	Others
BGC_055_amic	BGC_464_aspl	0.217307	0.612608	1	0.775972	1	0.224028	0	14	0	Others
BGC_031_neof	BGC_423_jaco	0.239226	0.578777	1	0.761623	0.6	0.238377	0	13	0	Others
BGC_052_amic	BGC_449_lacu	0.12595	0.763964	1	0.870155	1	0.129845	0	14	0	Others
BGC_080_effu	BGC_105_otit	0.213059	0.619277	0.928571	0.785507	0.785714	0.214493	0	13	0	Others
BGC_117_sput	BGC_484_terr	0.227174	0.597261	0.818182	0.775172	0.636364	0.224828	0	9	0	Others
BGC_055_amic	BGC_251_hydr	0.256154	0.553307	1	0.744171	0.6	0.255829	0	13	0	Others
BGC_222_mala	BGC_296_phth	0.211567	0.621626	1	0.781889	1	0.218111	0	13	0	Others
BGC_222_mala	BGC_345_zhao	0.175727	0.679427	1	0.818839	1	0.181161	0	13	0	Others
BGC_411_west	BGC_510_para	0.109477	0.793031	1	0.887137	1	0.112863	0	14	0	Others
BGC_187_sihw	BGC_327_insu	0.259385	0.54851	0.928571	0.744245	0.470588	0.255755	0	13	0	Others
BGC_055_amic	BGC_404_rubr	0.071697	0.861746	1	0.926085	1	0.073915	0	14	0	Others
BGC_314_alka	BGC_389_jing	0.272897	0.528679	0.543478	0.733282	0.519231	0.227899	0.288068	44	20	Others
BGC_309_alka	BGC_394_rubr	0.175317	0.680102	0.875	0.826101	0.730769	0.173899	0	25	0	Others
BGC_003_bron	BGC_386_jing	0.284184	0.512392	0.727273	0.721866	0.416667	0.278134	0	9	0	Others
BGC_251_hydr	BGC_464_aspl	0.260373	0.547048	1	0.739822	0.6	0.260178	0	13	0	Others
BGC_080_effu	BGC_327_insu	0.273046	0.528463	0.923077	0.721021	0.916667	0.278979	0	13	0	Others
BGC_277_spum	BGC_507_hank	0.234799	0.585533	0.928571	0.769591	0.470588	0.230409	0	13	0	Others

BGC_1	BGC_2	Raw distance	Squared similarity	Jaccard index	DSS index	Adjacency index	raw DSS non-anchor	raw DSS anchor	Non-anchor domains	Anchor domains	Network
BGC_390_jing	BGC_489_terr	0.151089	0.72065	0.904762	0.847184	0.904762	0.152816	0	20	0	Others
BGC_003_bron	BGC_277_spum	0.298642	0.491903	0.928571	0.703774	0.470588	0.296226	0	14	0	Others
BGC_140_nami	BGC_406_rubr	0.245985	0.568539	0.826087	0.755211	0.66	0.221319	0.304931	41	4	Others
BGC_384_jing	BGC_445_lacu	0.261257	0.545742	0.888889	0.735932	0.8	0.185968	0.384471	37	6	Others
BGC_003_bron	BGC_055_amic	0.196681	0.645322	0.928571	0.802391	0.785714	0.197609	0	14	0	Others
BGC_296_phth	BGC_510_para	0.244902	0.570174	1	0.755772	0.6	0.244228	0	13	0	Others
BGC_117_sput	BGC_222_mala	0.236844	0.582407	1	0.764078	0.6	0.235922	0	13	0	Others
BGC_114_sput	BGC_160_aich	0.140118	0.739398	1	0.855549	1	0.144451	0	22	0	Others
BGC_228_desu	BGC_404_rubr	0.201869	0.637012	0.928571	0.797042	0.785714	0.202958	0	13	0	Others
BGC_146_nami	BGC_165_aich	0.178968	0.674093	1	0.815497	1	0.184503	0	14	0	Others
BGC_003_bron	BGC_471_humi	0.298172	0.492562	0.928571	0.704258	0.470588	0.295742	0	14	0	Others
BGC_134_rhiz	BGC_206_soli	0.161535	0.703024	0.888889	0.837192	0.875	0.162808	0	8	0	Others
BGC_449_lacu	BGC_480_hank	0.287773	0.507268	0.928571	0.709562	0.733333	0.290438	0	14	0	Others
BGC_003_bron	BGC_080_effu	0.198842	0.641855	0.923077	0.79752	0.916667	0.20248	0	12	0	Others
BGC_362_amar	BGC_367_pseu	0.1866	0.66162	0.925926	0.813391	0.757576	0.18535	0.19196	34	2	Others
BGC_411_west	BGC_507_hank	0.175468	0.679854	0.928571	0.82426	0.785714	0.17574	0	13	0	Others
BGC_031_neof	BGC_222_mala	0.196772	0.645175	1	0.797142	1	0.202858	0	13	0	Others
BGC_146_nami	BGC_510_para	0.099088	0.811643	1	0.897848	1	0.102152	0	14	0	Others
BGC_017_poly	BGC_510_para	0.23578	0.584033	0.928571	0.759251	0.923077	0.240749	0	14	0	Others
BGC_117_sput	BGC_251_hydr	0.247457	0.56632	1	0.753137	0.6	0.246863	0	13	0	Others
BGC_447_lacu	BGC_507_hank	0.162491	0.701421	0.928571	0.837638	0.785714	0.162362	0	13	0	Others
BGC_309_alka	BGC_512_para	0.298187	0.492541	0.777778	0.705191	0.5	0.294809	0	25	0	Others
BGC_482_terr	BGC_518_para	0.28748	0.507684	0.861111	0.710439	0.73913	0.230692	0.377864	36	6	Others
BGC_449_lacu	BGC_489_terr	0.065358	0.873556	1	0.932621	1	0.067379	0	14	0	Others

BGC_1	BGC_2	Raw distance	Squared similarity	Jaccard index	DSS index	Adjacency index	raw DSS non-anchor	raw DSS anchor	Non-anchor domains	Anchor domains	Network
BGC_003_bron	BGC_355_amar	0.267337	0.536795	0.857143	0.731759	0.714286	0.268241	0	13	0	Others
BGC_114_sput	BGC_430_jaco	0.065403	0.873471	1	0.932574	1	0.067426	0	22	0	Others
BGC_017_poly	BGC_105_otit	0.221449	0.606141	0.928571	0.774024	0.923077	0.225976	0	14	0	Others
BGC_047_shan	BGC_296_phth	0.180746	0.671178	1	0.813664	1	0.186336	0	13	0	Others
BGC_105_otit	BGC_187_sihw	0.255581	0.554159	1	0.744762	0.6	0.255238	0	13	0	Others
BGC_031_neof	BGC_327_insu	0.258985	0.549104	0.928571	0.744657	0.470588	0.255343	0	13	0	Others
BGC_105_otit	BGC_345_zhao	0.252524	0.558721	1	0.747914	0.6	0.252086	0	13	0	Others
BGC_165_aich	BGC_386_jing	0.245329	0.569529	0.818182	0.756456	0.636364	0.243544	0	9	0	Others
BGC_228_desu	BGC_484_terr	0.267935	0.535918	0.727273	0.738617	0.416667	0.261383	0	8	0	Others
BGC_017_poly	BGC_296_phth	0.260597	0.546717	1	0.739591	0.6	0.260409	0	13	0	Others
BGC_003_bron	BGC_327_insu	0.170066	0.688791	1	0.824674	1	0.175326	0	14	0	Others
BGC_222_mala	BGC_371_pseu	0.296262	0.495247	0.785714	0.708382	0.4375	0.291618	0	12	0	Others
BGC_003_bron	BGC_105_otit	0.212503	0.620151	0.928571	0.786079	0.785714	0.213921	0	14	0	Others
BGC_017_poly	BGC_228_desu	0.230074	0.592786	0.857143	0.770174	0.714286	0.229826	0	13	0	Others
BGC_251_hydr	BGC_327_insu	0.245458	0.569334	0.928571	0.758603	0.470588	0.241397	0	13	0	Others
BGC_412_west	BGC_463_para	0.279467	0.519168	0.833333	0.720137	0.683333	0.24112	0.310857	64	20	Others
BGC_187_sihw	BGC_345_zhao	0.218424	0.610862	1	0.774821	1	0.225179	0	13	0	Others
BGC_165_aich	BGC_510_para	0.174952	0.680705	1	0.819637	1	0.180363	0	14	0	Others
BGC_203_soli	BGC_510_para	0.261247	0.545756	0.923077	0.736224	0.769231	0.263776	0	13	0	Others
BGC_022_poly	BGC_461_oryz	0.12556	0.764645	0.958333	0.873563	0.875	0.126437	0	24	0	Others
BGC_105_otit	BGC_203_soli	0.205923	0.630558	0.923077	0.793259	0.769231	0.206741	0	12	0	Others
BGC_143_nami	BGC_489_terr	0.207426	0.628173	0.95	0.787705	0.95	0.212295	0	21	0	Others
BGC_165_aich	BGC_423_jaco	0.052334	0.898072	1	0.946048	1	0.053952	0	14	0	Others
BGC_017_poly	BGC_404_rubr	0.252587	0.558626	0.928571	0.741923	0.923077	0.258077	0	14	0	Others

BGC_1	BGC_2	Raw distance	Squared similarity	Jaccard index	DSS index	Adjacency index	raw DSS non-anchor	raw DSS anchor	Non-anchor domains	Anchor domains	Network
BGC_384_jing	BGC_482_terr	0.251539	0.560194	0.842105	0.746792	0.782609	0.231933	0.294695	39	5	Others
BGC_031_neof	BGC_510_para	0.240406	0.576984	1	0.760406	0.6	0.239593	0	13	0	Others
BGC_404_rubr	BGC_411_west	0.048533	0.905289	1	0.949965	1	0.050035	0	14	0	Others
BGC_095_otit	BGC_160_aich	0.123873	0.767599	1	0.872296	1	0.127704	0	22	0	Others
BGC_052_amic	BGC_489_terr	0.226076	0.598958	0.904762	0.771662	0.818182	0.228338	0	22	0	Others
BGC_203_soli	BGC_507_hank	0.256305	0.553082	0.846154	0.74619	0.571429	0.25381	0	12	0	Others
BGC_126_rhiz	BGC_309_alka	0.287727	0.507333	0.68	0.716983	0.5	0.283017	0	19	0	Others
BGC_251_hydr	BGC_404_rubr	0.257563	0.551212	1	0.742718	0.6	0.257282	0	13	0	Others
BGC_080_effu	BGC_117_sput	0.208754	0.62607	0.928571	0.789944	0.785714	0.210056	0	13	0	Others
BGC_047_shan	BGC_471_humi	0.194557	0.648739	1	0.799426	1	0.200574	0	13	0	Others
BGC_187_sihw	BGC_222_mala	0.201976	0.636843	1	0.791778	1	0.208222	0	13	0	Others
BGC_055_amic	BGC_080_effu	0.266336	0.538263	0.928571	0.730582	0.785714	0.269418	0	14	0	Others
BGC_105_otit	BGC_296_phth	0.248759	0.564363	1	0.751795	0.6	0.248205	0	13	0	Others
BGC_165_aich	BGC_311_alka	0.24144	0.575413	0.818182	0.760465	0.636364	0.239535	0	9	0	Others
BGC_061_amic	BGC_140_nami	0.171701	0.68608	0.795455	0.833093	0.612245	0.197203	0.096848	37	4	Others
BGC_251_hydr	BGC_345_zhao	0.217034	0.613035	1	0.776253	1	0.223747	0	13	0	Others
BGC_122_rhiz	BGC_464_aspl	0.281355	0.51645	0.928571	0.715098	0.785714	0.284902	0	14	0	Others
BGC_031_neof	BGC_080_effu	0.250918	0.561124	0.923077	0.751737	0.533333	0.248263	0	12	0	Others
BGC_441_lacu	BGC_486_terr	0.159626	0.706228	0.863636	0.842427	0.729167	0.159504	0.151137	40	3	Others
BGC_061_amic	BGC_313_alka	0.244904	0.570171	0.755556	0.759341	0.54902	0.299667	0.096828	39	4	Others
BGC_165_aich	BGC_222_mala	0.239981	0.577629	1	0.760844	0.6	0.239156	0	13	0	Others
BGC_017_poly	BGC_251_hydr	0.262003	0.54464	1	0.738142	0.6	0.261858	0	13	0	Others
BGC_277_spum	BGC_411_west	0.249602	0.563097	1	0.750926	0.6	0.249074	0	13	0	Others
BGC_165_aich	BGC_355_amar	0.262447	0.543985	0.785714	0.741267	0.533333	0.258733	0	12	0	Others

BGC_1	BGC_2	Raw distance	Squared similarity	Jaccard index	DSS index	Adjacency index	raw DSS non-anchor	raw DSS anchor	Non-anchor domains	Anchor domains	Network
BGC_277_spum	BGC_345_zhao	0.173741	0.682704	1	0.820886	1	0.179114	0	13	0	Others
BGC_047_shan	BGC_327_insu	0.268598	0.534949	0.928571	0.734747	0.470588	0.265253	0	13	0	Others
BGC_277_spum	BGC_464_aspl	0.259816	0.547873	1	0.740396	0.6	0.259604	0	13	0	Others
BGC_277_spum	BGC_447_lacu	0.244075	0.571423	1	0.756624	0.6	0.243376	0	13	0	Others
BGC_031_neof	BGC_117_sput	0.241794	0.574876	1	0.758975	0.6	0.241025	0	13	0	Others
BGC_146_nami	BGC_423_jaco	0.182686	0.668002	1	0.811664	1	0.188336	0	14	0	Others
BGC_390_jing	BGC_512_para	0.215063	0.616125	0.826087	0.786092	0.708333	0.213908	0	20	0	Others
BGC_105_otit	BGC_404_rubr	0.190001	0.656099	1	0.804123	1	0.195877	0	14	0	Others
BGC_003_bron	BGC_411_west	0.219138	0.609746	0.928571	0.779239	0.785714	0.220761	0	14	0	Others
BGC_017_poly	BGC_355_amar	0.26123	0.545782	0.785714	0.742522	0.533333	0.257478	0	12	0	Others
BGC_165_aich	BGC_187_sihw	0.250467	0.5618	1	0.750034	0.6	0.249966	0	13	0	Others
BGC_327_insu	BGC_371_pseu	0.283079	0.513976	0.857143	0.71553	0.714286	0.28447	0	13	0	Others
BGC_345_zhao	BGC_447_lacu	0.265772	0.539091	1	0.734256	0.6	0.265744	0	13	0	Others
BGC_003_bron	BGC_464_aspl	0.267461	0.536614	0.928571	0.729422	0.785714	0.270578	0	14	0	Others
BGC_228_desu	BGC_345_zhao	0.299446	0.490776	0.857143	0.703075	0.5	0.296925	0	13	0	Others
BGC_311_alka	BGC_510_para	0.272207	0.529682	0.818182	0.728746	0.636364	0.271254	0	11	0	Others
BGC_345_zhao	BGC_411_west	0.276037	0.524123	1	0.723674	0.6	0.276326	0	13	0	Others
BGC_047_shan	BGC_251_hydr	0.172481	0.684788	1	0.822185	1	0.177815	0	13	0	Others
BGC_122_rhiz	BGC_165_aich	0.244551	0.570703	0.928571	0.75304	0.785714	0.24696	0	14	0	Others
BGC_411_west	BGC_484_terr	0.172423	0.684883	0.818182	0.831616	0.636364	0.168384	0	9	0	Others
BGC_206_soli	BGC_496_hank	0.275454	0.524967	0.944444	0.720185	0.826087	0.275523	0.293761	26	2	Others
BGC_146_nami	BGC_311_alka	0.056042	0.891056	0.818182	0.951596	0.636364	0.048404	0	9	0	Others
BGC_117_sput	BGC_228_desu	0.246808	0.567299	0.928571	0.750714	0.785714	0.249286	0	14	0	Others
BGC_055_amic	BGC_507_hank	0.155079	0.713891	0.928571	0.845279	0.785714	0.154721	0	13	0	Others

BGC_1	BGC_2	Raw distance	Squared similarity	Jaccard index	DSS index	Adjacency index	raw DSS non-anchor	raw DSS anchor	Non-anchor domains	Anchor domains	Network
BGC_327_insu	BGC_423_jaco	0.225326	0.60012	0.928571	0.77286	0.785714	0.22714	0	14	0	Others
BGC_143_nami	BGC_309_alka	0.079402	0.8475	0.875	0.924982	0.730769	0.075018	0	22	0	Others
BGC_146_nami	BGC_386_jing	0.134804	0.748564	0.818182	0.870399	0.636364	0.129601	0	9	0	Others
BGC_311_alka	BGC_386_jing	0.132202	0.753074	1	0.863709	1	0.136291	0	11	0	Others
BGC_122_rhiz	BGC_447_lacu	0.250889	0.561168	0.928571	0.746507	0.785714	0.253493	0	14	0	Others
BGC_080_effu	BGC_510_para	0.208877	0.625876	0.928571	0.789818	0.785714	0.210182	0	13	0	Others
BGC_055_amic	BGC_165_aich	0.169754	0.689308	1	0.824996	1	0.175004	0	14	0	Others
BGC_402_rubr	BGC_414_west	0.294496	0.497737	0.756098	0.706064	0.653061	0.216251	0.437652	37	5	Others
BGC_222_mala	BGC_411_west	0.264395	0.541115	1	0.735676	0.6	0.264324	0	13	0	Others
BGC_277_spum	BGC_327_insu	0.253192	0.557722	0.928571	0.750629	0.470588	0.249371	0	13	0	Others
BGC_464_aspl	BGC_471_humi	0.265926	0.538865	1	0.734097	0.6	0.265903	0	13	0	Others
BGC_031_neof	BGC_277_spum	0.190289	0.655633	1	0.803826	1	0.196174	0	13	0	Others
BGC_389_jing	BGC_412_west	0.280625	0.517501	0.829787	0.717974	0.732143	0.273014	0.289461	66	20	Others
BGC_047_shan	BGC_165_aich	0.253076	0.557895	1	0.747344	0.6	0.252656	0	13	0	Others
BGC_143_nami	BGC_421_west	0.147833	0.726188	0.904762	0.853955	0.73913	0.146045	0	21	0	Others
BGC_251_hydr	BGC_471_humi	0.206508	0.62963	1	0.787105	1	0.212895	0	13	0	Others
BGC_017_poly	BGC_146_nami	0.239915	0.577729	0.928571	0.754987	0.923077	0.245013	0	14	0	Others
BGC_296_phth	BGC_471_humi	0.209794	0.624425	1	0.783717	1	0.216283	0	13	0	Others
BGC_229_desu	BGC_430_jaco	0.260486	0.546881	0.789474	0.740139	0.684211	0.259861	0	15	0	Others
BGC_105_otit	BGC_355_amar	0.260442	0.546946	0.785714	0.743334	0.533333	0.256666	0	12	0	Others
BGC_165_aich	BGC_507_hank	0.157213	0.71029	0.928571	0.843079	0.785714	0.156921	0	13	0	Others
BGC_047_shan	BGC_080_effu	0.255049	0.554952	0.923077	0.747478	0.533333	0.252522	0	12	0	Others
BGC_464_aspl	BGC_510_para	0.209326	0.625165	1	0.7842	1	0.2158	0	14	0	Others
BGC_047_shan	BGC_447_lacu	0.261382	0.545557	1	0.738782	0.6	0.261218	0	13	0	Others

BGC_1	BGC_2	Raw distance	Squared similarity	Jaccard index	DSS index	Adjacency index	raw DSS non-anchor	raw DSS anchor	Non-anchor domains	Anchor domains	Network
BGC_296_phth	BGC_507_hank	0.239761	0.577964	0.928571	0.764476	0.470588	0.235524	0	13	0	Others
BGC_390_jing	BGC_421_west	0.198771	0.641968	0.9	0.801736	0.727273	0.198264	0	20	0	Others
BGC_404_rubr	BGC_471_humi	0.268473	0.535131	1	0.731471	0.6	0.268529	0	13	0	Others
BGC_222_mala	BGC_510_para	0.247733	0.565906	1	0.752853	0.6	0.247147	0	13	0	Others
BGC_117_sput	BGC_345_zhao	0.253074	0.557898	1	0.747346	0.6	0.252654	0	13	0	Others
BGC_122_rhiz	BGC_327_insu	0.159395	0.706617	1	0.835676	1	0.164324	0	14	0	Others
BGC_222_mala	BGC_228_desu	0.296913	0.494331	0.857143	0.705686	0.5	0.294314	0	13	0	Others
BGC_117_sput	BGC_464_aspl	0.214395	0.617175	1	0.778974	1	0.221026	0	14	0	Others
BGC_423_jaco	BGC_464_aspl	0.215011	0.616208	1	0.778339	1	0.221661	0	14	0	Others
BGC_296_phth	BGC_423_jaco	0.24148	0.575352	1	0.759299	0.6	0.240701	0	13	0	Others
BGC_031_neof	BGC_146_nami	0.25084	0.56124	1	0.749649	0.6	0.250351	0	13	0	Others
BGC_055_amic	BGC_187_sihw	0.249531	0.563204	1	0.750999	0.6	0.249001	0	13	0	Others
BGC_447_lacu	BGC_510_para	0.126129	0.763651	1	0.86997	1	0.13003	0	14	0	Others
BGC_407_rubr	BGC_412_west	0.189254	0.657309	0.843137	0.811918	0.737705	0.203	0.175215	69	20	Others
BGC_047_shan	BGC_222_mala	0.199847	0.640244	1	0.793972	1	0.206028	0	13	0	Others
BGC_277_spum	BGC_423_jaco	0.232361	0.58927	1	0.7687	0.6	0.2313	0	13	0	Others
BGC_031_neof	BGC_319_iter	0.279581	0.519004	0.888889	0.726039	0.363636	0.273961	0	8	0	Others
BGC_146_nami	BGC_447_lacu	0.112183	0.78822	1	0.884348	1	0.115652	0	14	0	Others
BGC_047_shan	BGC_105_otit	0.25956	0.548251	1	0.74066	0.6	0.25934	0	13	0	Others
BGC_080_effu	BGC_146_nami	0.208123	0.627069	0.928571	0.790595	0.785714	0.209405	0	13	0	Others
BGC_055_amic	BGC_355_amar	0.264934	0.540322	0.785714	0.738703	0.533333	0.261297	0	12	0	Others
BGC_222_mala	BGC_319_iter	0.291307	0.502245	0.888889	0.71395	0.363636	0.28605	0	8	0	Others
BGC_146_nami	BGC_411_west	0.057165	0.888938	1	0.941067	1	0.058933	0	14	0	Others
BGC_187_sihw	BGC_251_hydr	0.154702	0.714528	1	0.840513	1	0.159487	0	13	0	Others

BGC_1	BGC_2	Raw distance	Squared similarity	Jaccard index	DSS index	Adjacency index	raw DSS non-anchor	raw DSS anchor	Non-anchor domains	Anchor domains	Network
BGC_031_neof	BGC_187_sihw	0.089074	0.829787	1	0.908172	1	0.091828	0	13	0	Others
BGC_187_sihw	BGC_319_iter	0.288343	0.506455	0.888889	0.717005	0.363636	0.282995	0	8	0	Others
BGC_345_zhao	BGC_423_jaco	0.251283	0.560577	1	0.749193	0.6	0.250807	0	13	0	Others
BGC_228_desu	BGC_464_aspl	0.212976	0.619406	0.928571	0.785591	0.785714	0.214408	0	13	0	Others
BGC_146_nami	BGC_355_amar	0.273865	0.527272	0.785714	0.729496	0.533333	0.270504	0	12	0	Others
BGC_117_sput	BGC_355_amar	0.262759	0.543525	0.785714	0.740946	0.533333	0.259054	0	12	0	Others
BGC_055_amic	BGC_311_alka	0.079365	0.847569	0.818182	0.927553	0.636364	0.072447	0	9	0	Others
BGC_228_desu	BGC_411_west	0.194998	0.648028	0.928571	0.804126	0.785714	0.195874	0	13	0	Others
BGC_449_lacu	BGC_461_oryz	0.272035	0.529933	0.928571	0.724706	0.785714	0.275294	0	13	0	Others
BGC_117_sput	BGC_327_insu	0.167634	0.692833	0.928571	0.832336	0.785714	0.167664	0	13	0	Others
BGC_080_effu	BGC_228_desu	0.273256	0.528156	0.857143	0.728013	0.6	0.271987	0	13	0	Others
BGC_031_neof	BGC_122_rhiz	0.273448	0.527877	0.928571	0.729746	0.470588	0.270254	0	13	0	Others
BGC_224_mala	BGC_271_spum	0.295205	0.496736	0.75	0.707294	0.560976	0.292706	0	35	0	Others
BGC_394_rubr	BGC_489_terr	0.234298	0.5863	0.904762	0.761401	0.904762	0.238598	0	22	0	Others
BGC_327_insu	BGC_447_lacu	0.228609	0.595044	0.928571	0.769475	0.785714	0.230525	0	14	0	Others
BGC_122_rhiz	BGC_423_jaco	0.240616	0.576664	0.928571	0.757097	0.785714	0.242903	0	14	0	Others
BGC_031_neof	BGC_203_soli	0.279774	0.518725	0.916667	0.722742	0.5	0.277258	0	11	0	Others
BGC_105_otit	BGC_251_hydr	0.256407	0.552931	1	0.74391	0.6	0.25609	0	13	0	Others
BGC_146_nami	BGC_203_soli	0.192852	0.651488	0.923077	0.806734	0.769231	0.193266	0	12	0	Others
BGC_327_insu	BGC_411_west	0.230972	0.591404	0.928571	0.767039	0.785714	0.232961	0	14	0	Others
BGC_047_shan	BGC_055_amic	0.25871	0.54951	1	0.741536	0.6	0.258464	0	13	0	Others
BGC_117_sput	BGC_447_lacu	0.175688	0.67949	1	0.818878	1	0.181122	0	14	0	Others
BGC_447_lacu	BGC_484_terr	0.064852	0.874502	0.818182	0.942514	0.636364	0.057485	0	9	0	Others
BGC_309_alka	BGC_449_lacu	0.189368	0.657124	0.928571	0.809635	0.8	0.190365	0	15	0	Others

BGC_1	BGC_2	Raw distance	Squared similarity	Jaccard index	DSS index	Adjacency index	raw DSS non-anchor	raw DSS anchor	Non-anchor domains	Anchor domains	Network
BGC_047_shan	BGC_228_desu	0.295413	0.496443	0.857143	0.707233	0.5	0.292767	0	13	0	Others
BGC_150_nami	BGC_482_terr	0.266776	0.537617	0.870968	0.730762	0.783784	0.221729	0.358318	30	4	Others
BGC_055_amic	BGC_423_jaco	0.175601	0.679634	1	0.818968	1	0.181032	0	14	0	Others
BGC_309_alka	BGC_421_west	0.239646	0.578138	0.791667	0.764663	0.535714	0.235337	0	24	0	Others
BGC_095_otit	BGC_430_jaco	0.131393	0.754478	1	0.864543	1	0.135457	0	22	0	Others
BGC_222_mala	BGC_507_hank	0.238969	0.579168	0.928571	0.765292	0.470588	0.234708	0	13	0	Others
BGC_251_hydr	BGC_411_west	0.252914	0.558137	1	0.747511	0.6	0.252489	0	13	0	Others
BGC_140_nami	BGC_313_alka	0.226046	0.599005	0.804348	0.777928	0.566038	0.26211	0.116975	42	4	Others
BGC_003_bron	BGC_017_poly	0.220127	0.608202	0.928571	0.77822	0.785714	0.22178	0	14	0	Others
BGC_061_amic	BGC_409_west	0.19476	0.648411	0.813953	0.807409	0.695652	0.239621	0.077954	39	4	Others
BGC_251_hydr	BGC_447_lacu	0.265188	0.539948	1	0.734858	0.6	0.265142	0	13	0	Others
BGC_311_alka	BGC_447_lacu	0.292963	0.499901	0.818182	0.707348	0.636364	0.292652	0	11	0	Others
BGC_117_sput	BGC_122_rhiz	0.183145	0.667252	0.928571	0.816345	0.785714	0.183655	0	13	0	Others
BGC_055_amic	BGC_222_mala	0.249609	0.563086	1	0.750918	0.6	0.249082	0	13	0	Others
BGC_017_poly	BGC_345_zhao	0.263486	0.542453	1	0.736612	0.6	0.263388	0	13	0	Others
BGC_421_west	BGC_489_terr	0.202191	0.6365	0.9	0.79821	0.727273	0.20179	0	20	0	Others
BGC_047_shan	BGC_423_jaco	0.253418	0.557385	1	0.746992	0.6	0.253008	0	13	0	Others
BGC_311_alka	BGC_411_west	0.249505	0.563242	0.818182	0.75215	0.636364	0.24785	0	11	0	Others
BGC_404_rubr	BGC_447_lacu	0.121319	0.77208	1	0.874929	1	0.125071	0	14	0	Others
BGC_471_humi	BGC_507_hank	0.247015	0.566986	0.928571	0.756997	0.470588	0.243003	0	13	0	Others
BGC_052_amic	BGC_143_nami	0.138285	0.742552	0.952381	0.86074	0.863636	0.13926	0	22	0	Others
BGC_251_hydr	BGC_277_spum	0.210116	0.623917	1	0.783386	1	0.216614	0	13	0	Others
BGC_203_soli	BGC_404_rubr	0.268557	0.535008	0.923077	0.728688	0.769231	0.271312	0	13	0	Others
BGC_117_sput	BGC_404_rubr	0.187906	0.659497	1	0.806283	1	0.193717	0	14	0	Others

BGC_1	BGC_2	Raw distance	Squared similarity	Jaccard index	DSS index	Adjacency index	raw DSS non-anchor	raw DSS anchor	Non-anchor domains	Anchor domains	Network
BGC_150_nami	BGC_384_jing	0.227947	0.596065	0.870968	0.770791	0.783784	0.214671	0.256467	30	4	Others
BGC_105_otit	BGC_165_aich	0.038536	0.924413	1	0.960272	1	0.039728	0	14	0	Others
BGC_017_poly	BGC_222_mala	0.249526	0.563211	1	0.751004	0.6	0.248996	0	13	0	Others
BGC_277_spum	BGC_471_humi	0.138072	0.74292	1	0.857658	1	0.142342	0	13	0	Others
BGC_296_phth	BGC_411_west	0.260788	0.546435	1	0.739394	0.6	0.260606	0	13	0	Others
BGC_105_otit	BGC_447_lacu	0.181259	0.670337	1	0.813135	1	0.186865	0	14	0	Others
BGC_327_insu	BGC_464_aspl	0.276746	0.523096	0.928571	0.719849	0.785714	0.280151	0	14	0	Others
BGC_047_shan	BGC_507_hank	0.249711	0.562934	0.928571	0.754218	0.470588	0.245782	0	13	0	Others
BGC_296_phth	BGC_447_lacu	0.259285	0.548659	1	0.740943	0.6	0.259057	0	13	0	Others
BGC_105_otit	BGC_411_west	0.190011	0.656083	1	0.804113	1	0.195887	0	14	0	Others
BGC_146_nami	BGC_277_spum	0.245142	0.569811	1	0.755524	0.6	0.244476	0	13	0	Others
BGC_203_soli	BGC_464_aspl	0.29336	0.49934	0.923077	0.703118	0.769231	0.296882	0	13	0	Others
BGC_146_nami	BGC_471_humi	0.25741	0.55144	1	0.742877	0.6	0.257123	0	13	0	Others
BGC_332_insu	BGC_496_hank	0.113051	0.786679	0.5	0.900736	0.411765	0.099264	0	15	0	Others
BGC_143_nami	BGC_390_jing	0.188288	0.658877	0.95	0.807436	0.95	0.192565	0	21	0	Others
BGC_122_rhiz	BGC_146_nami	0.252048	0.559432	0.928571	0.745311	0.785714	0.254689	0	14	0	Others
BGC_296_phth	BGC_327_insu	0.260929	0.546225	0.928571	0.742653	0.470588	0.257347	0	13	0	Others
BGC_187_sihw	BGC_404_rubr	0.266362	0.538225	1	0.733648	0.6	0.266352	0	13	0	Others
BGC_031_neof	BGC_404_rubr	0.258216	0.550243	1	0.742045	0.6	0.257955	0	13	0	Others
BGC_017_poly	BGC_080_effu	0.205166	0.631762	0.923077	0.791	0.916667	0.209	0	12	0	Others
BGC_146_nami	BGC_228_desu	0.244504	0.570775	0.928571	0.753089	0.785714	0.246911	0	14	0	Others
BGC_117_sput	BGC_311_alka	0.238031	0.580596	0.818182	0.763979	0.636364	0.236021	0	9	0	Others
BGC_371_pseu	BGC_507_hank	0.268678	0.534832	0.857143	0.730376	0.714286	0.269624	0	13	0	Others
BGC_055_amic	BGC_510_para	0.097201	0.815046	1	0.899793	1	0.100207	0	14	0	Others

BGC_1	BGC_2	Raw distance	Squared similarity	Jaccard index	DSS index	Adjacency index	raw DSS non-anchor	raw DSS anchor	Non-anchor domains	Anchor domains	Network
BGC_303_alka	BGC_518_para	0.279321	0.519378	0.942857	0.715909	0.840909	0.186067	0.43521	37	6	Others
BGC_003_bron	BGC_484_terr	0.293693	0.49887	0.727273	0.712063	0.416667	0.287937	0	9	0	Others
BGC_047_shan	BGC_203_soli	0.274292	0.526652	0.916667	0.728393	0.5	0.271607	0	11	0	Others
BGC_080_effu	BGC_165_aich	0.213174	0.619095	0.928571	0.785388	0.785714	0.214612	0	13	0	Others
BGC_080_effu	BGC_507_hank	0.260658	0.546627	0.923077	0.733792	0.916667	0.266208	0	13	0	Others
BGC_150_nami	BGC_518_para	0.284915	0.511347	0.966667	0.708908	0.888889	0.166898	0.477382	30	5	Others
BGC_017_poly	BGC_327_insu	0.196771	0.645177	0.928571	0.802298	0.785714	0.197702	0	13	0	Others
BGC_222_mala	BGC_404_rubr	0.271369	0.530903	1	0.728485	0.6	0.271515	0	13	0	Others
BGC_055_amic	BGC_122_rhiz	0.191553	0.653587	0.928571	0.807678	0.785714	0.192322	0	13	0	Others
BGC_222_mala	BGC_327_insu	0.25245	0.558832	0.928571	0.751395	0.470588	0.248605	0	13	0	Others
BGC_165_aich	BGC_251_hydr	0.246605	0.567604	1	0.754015	0.6	0.245985	0	13	0	Others
BGC_187_sihw	BGC_228_desu	0.283854	0.512865	0.857143	0.719149	0.5	0.280851	0	13	0	Others
BGC_187_sihw	BGC_296_phth	0.146047	0.729235	1	0.849436	1	0.150564	0	13	0	Others
BGC_150_nami	BGC_402_rubr	0.082718	0.841406	0.84375	0.92135	0.756757	0.060767	0.108828	27	4	Others
BGC_209_soli	BGC_513_para	0.238943	0.579208	0.8	0.753618	0.615385	0.262623	0.116457	24	3	PKSI
BGC_180_hirs	BGC_342_zhao	0.217976	0.611561	0.903226	0.746182	0.810811	0.262686	0.150365	35	3	PKSI
BGC_001_bron	BGC_252_hydr	0.280763	0.517302	0.606061	0.755278	0.594595	0.251524	0.199373	20	3	PKSI
BGC_001_bron	BGC_294_phth	0.27821	0.520981	0.588235	0.76421	0.578947	0.240499	0.204396	20	3	PKSI
BGC_243_desu	BGC_520_aspl	0.182208	0.668784	1	0.760253	1	0.266876	0.131231	12	3	PKSI
BGC_036_neof	BGC_317_iter	0.296124	0.495442	0.763158	0.687695	0.666667	0.324924	0.13984	41	3	PKSI
BGC_001_bron	BGC_243_desu	0.145985	0.729341	1	0.807914	1	0.215323	0.099136	12	3	PKSI
BGC_148_nami	BGC_243_desu	0.164378	0.698264	1	0.783713	1	0.226408	0.175802	12	3	PKSI
BGC_501_hank	BGC_513_para	0.263057	0.543086	0.757576	0.733051	0.657895	0.282008	0.141457	25	3	PKSI
BGC_082_effu	BGC_209_soli	0.22121	0.606515	0.75	0.789613	0.684211	0.225896	0.153518	11	3	PKSI

BGC_1	BGC_2	Raw distance	Squared similarity	Jaccard index	DSS index	Adjacency index	raw DSS non-anchor	raw DSS anchor	Non-anchor domains	Anchor domains	Network
BGC_119_sput	BGC_128_rhiz	0.274817	0.52589	0.736842	0.723347	0.666667	0.307007	0.124878	15	3	PKSI
BGC_036_neof	BGC_294_phth	0.163777	0.699268	0.857143	0.833376	0.714286	0.17247	0.094526	37	3	PKSI
BGC_272_spum	BGC_342_zhao	0.209347	0.625132	0.885714	0.763402	0.780488	0.244886	0.126087	40	3	PKSI
BGC_046_shan	BGC_243_desu	0.205322	0.631512	0.923077	0.757369	0.8	0.259878	0.179389	11	3	PKSI
BGC_304_alka	BGC_513_para	0.123429	0.768376	0.939394	0.859185	0.846154	0.146683	0.062575	40	3	PKSI
BGC_243_desu	BGC_317_iter	0.239044	0.579054	0.923077	0.712999	0.8	0.320643	0.152433	12	3	PKSI
BGC_209_soli	BGC_304_alka	0.228014	0.595962	0.842105	0.755161	0.64	0.259885	0.124468	24	3	PKSI
BGC_001_bron	BGC_336_insu	0.254049	0.556443	0.736842	0.750873	0.659091	0.261587	0.128675	29	3	PKSI
BGC_001_bron	BGC_082_effu	0.296384	0.495075	0.59375	0.740085	0.526316	0.278353	0.130853	21	3	PKSI
BGC_097_otit	BGC_455_oryz	0.244908	0.570165	1	0.67885	0.958333	0.350852	0.234521	35	12	PKSI
BGC_243_desu	BGC_467_humi	0.24148	0.575353	0.923077	0.709794	0.8	0.329019	0.134954	12	3	PKSI
BGC_434_jaco	BGC_458_oryz	0.298661	0.491877	0.933333	0.635309	0.658537	0.379794	0.158274	41	3	PKSI
BGC_013_poly	BGC_458_oryz	0.101845	0.806683	0.862069	0.911038	0.805556	0.095129	0.012903	37	3	PKSI
BGC_046_shan	BGC_272_spum	0.271639	0.530509	0.763158	0.719513	0.681818	0.290611	0.148882	39	3	PKSI
BGC_036_neof	BGC_243_desu	0.193522	0.650406	1	0.745365	1	0.280187	0.152424	12	3	PKSI
BGC_099_otit	BGC_128_rhiz	0.182568	0.668195	0.823529	0.815249	0.833333	0.20153	0.112046	13	3	PKSI
BGC_119_sput	BGC_434_jaco	0.034345	0.93249	1	0.95481	1	0.04961	0.002469	29	3	PKSI
BGC_243_desu	BGC_336_insu	0.172403	0.684916	1	0.773153	1	0.249717	0.135365	12	3	PKSI
BGC_036_neof	BGC_252_hydr	0.150327	0.721945	0.848485	0.852809	0.74359	0.152469	0.092651	31	3	PKSI
BGC_220_mala	BGC_272_spum	0.117932	0.778043	0.939394	0.866418	0.846154	0.137708	0.079936	39	3	PKSI
BGC_317_iter	BGC_342_zhao	0.235968	0.583745	0.878788	0.730002	0.794872	0.27953	0.152434	37	3	PKSI
BGC_046_shan	BGC_317_iter	0.208667	0.626208	0.941176	0.746413	0.85	0.260791	0.157529	40	3	PKSI
BGC_099_otit	BGC_119_sput	0.080665	0.845177	0.916667	0.921883	0.851852	0.083468	0.029953	27	3	PKSI
BGC_220_mala	BGC_294_phth	0.275006	0.525617	0.763158	0.718223	0.5625	0.292101	0.147562	39	3	PKSI

BGC_1	BGC_2	Raw distance	Squared similarity	Jaccard index	DSS index	Adjacency index	raw DSS non-anchor	raw DSS anchor	Non-anchor domains	Anchor domains	Network
BGC_336_insu	BGC_501_hank	0.228835	0.594696	0.85	0.749041	0.744681	0.25944	0.12657	44	3	PKSI
BGC_189_sihw	BGC_252_hydr	0.242721	0.573472	0.783784	0.754089	0.586957	0.258512	0.094695	36	3	PKSI
BGC_128_rhiz	BGC_434_jaco	0.299129	0.49122	0.736842	0.691359	0.666667	0.343097	0.124878	16	3	PKSI
BGC_071_arai	BGC_259_croc	0.262462	0.543962	0.8	0.720167	0.710526	0.300722	0.081382	38	4	PKSI
BGC_243_desu	BGC_272_spum	0.247799	0.565806	0.923077	0.701478	0.8	0.330508	0.170575	12	3	PKSI
BGC_046_shan	BGC_082_effu	0.29617	0.495376	0.709677	0.703589	0.648649	0.307073	0.204011	26	3	PKSI
BGC_189_sihw	BGC_243_desu	0.185352	0.663652	1	0.756116	1	0.263674	0.164723	12	3	PKSI
BGC_036_neof	BGC_046_shan	0.227707	0.596436	0.810811	0.762926	0.704545	0.246451	0.10893	41	3	PKSI
BGC_036_neof	BGC_128_rhiz	0.225116	0.600445	0.823529	0.759264	0.833333	0.259719	0.158475	13	3	PKSI
BGC_364_amar	BGC_380_pseu	0.153395	0.71674	0.807692	0.860624	0.741935	0.148304	0.070927	23	3	PKSI
BGC_220_mala	BGC_243_desu	0.217695	0.612001	0.923077	0.74109	0.8	0.285257	0.162305	11	3	PKSI
BGC_170_aich	BGC_243_desu	0.181999	0.669126	1	0.760528	1	0.257933	0.165627	12	3	PKSI
BGC_148_nami	BGC_513_para	0.284616	0.511774	0.666667	0.731284	0.647059	0.294534	0.053571	25	3	PKSI
BGC_036_neof	BGC_272_spum	0.204548	0.632745	0.888889	0.770797	0.704545	0.238077	0.110875	40	3	PKSI
BGC_082_effu	BGC_128_rhiz	0.252578	0.55864	0.722222	0.759349	0.571429	0.261651	0.149651	13	3	PKSI
BGC_243_desu	BGC_434_jaco	0.174049	0.682195	1	0.770988	1	0.246299	0.159867	12	3	PKSI
BGC_180_hirs	BGC_243_desu	0.198654	0.642155	0.923077	0.766143	0.8	0.258484	0.143559	11	3	PKSI
BGC_128_rhiz	BGC_336_insu	0.288466	0.506281	0.764706	0.694399	0.777778	0.341	0.116806	16	3	PKSI
BGC_180_hirs	BGC_294_phth	0.283624	0.513195	0.722222	0.718584	0.568182	0.291831	0.163387	34	3	PKSI
BGC_148_nami	BGC_382_jing	0.157751	0.709383	0.913043	0.821503	0.851852	0.18369	0.093683	49	3	PKSI
BGC_148_nami	BGC_209_soli	0.245288	0.56959	0.705882	0.77367	0.571429	0.253302	0.118441	12	3	PKSI
BGC_148_nami	BGC_304_alka	0.177356	0.676743	0.689655	0.865245	0.666667	0.151139	0.009144	23	3	PKSI
BGC_001_bron	BGC_458_oryz	0.276188	0.523903	0.724138	0.727829	0.567568	0.295136	0.096105	23	3	PKSI
BGC_082_effu	BGC_243_desu	0.192219	0.65251	0.923077	0.774611	0.8	0.246679	0.147327	11	3	PKSI

BGC_1	BGC_2	Raw distance	Squared similarity	Jaccard index	DSS index	Adjacency index	raw DSS non-anchor	raw DSS anchor	Non-anchor domains	Anchor domains	Network
BGC_001_bron	BGC_513_para	0.205978	0.63047	0.878788	0.769604	0.789474	0.240511	0.132623	29	3	PKSI
BGC_243_desu	BGC_458_oryz	0.199299	0.641122	0.923077	0.765295	0.8	0.271525	0.087428	12	3	PKSI
BGC_036_neof	BGC_342_zhao	0.228175	0.595714	0.885714	0.740196	0.72093	0.271465	0.100435	41	3	PKSI
BGC_001_bron	BGC_036_neof	0.283274	0.513696	0.588235	0.757546	0.578947	0.252988	0.172227	20	3	PKSI
BGC_272_spum	BGC_294_phth	0.259613	0.548173	0.756757	0.740575	0.553191	0.269559	0.134443	37	3	PKSI
BGC_001_bron	BGC_189_sihw	0.274847	0.525847	0.588235	0.768635	0.578947	0.23963	0.176267	20	3	PKSI
BGC_001_bron	BGC_520_aspl	0.291458	0.502032	0.512821	0.770686	0.5	0.244389	0.123788	21	3	PKSI
BGC_036_neof	BGC_180_hirs	0.281123	0.516784	0.848485	0.681198	0.725	0.331752	0.146139	40	3	PKSI
BGC_001_bron	BGC_128_rhiz	0.212062	0.620846	0.764706	0.79493	0.777778	0.225321	0.124065	12	3	PKSI
BGC_209_soli	BGC_243_desu	0.178335	0.675133	0.923077	0.792879	0.8	0.217908	0.167571	11	3	PKSI
BGC_099_otit	BGC_170_aich	0.106701	0.797983	0.916667	0.888426	0.821429	0.120272	0.030395	28	3	PKSI
BGC_382_jing	BGC_458_oryz	0.25303	0.557965	0.758621	0.745711	0.666667	0.264968	0.168856	24	3	PKSI
BGC_243_desu	BGC_294_phth	0.182632	0.66809	1	0.759695	1	0.255979	0.177609	12	3	PKSI
BGC_001_bron	BGC_170_aich	0.28319	0.513817	0.625	0.746656	0.592593	0.278618	0.135398	14	3	PKSI
BGC_001_bron	BGC_119_sput	0.278803	0.520125	0.625	0.751828	0.615385	0.273493	0.130008	14	3	PKSI
BGC_001_bron	BGC_304_alka	0.20738	0.628247	0.875	0.770241	0.736842	0.238064	0.14948	29	3	PKSI
BGC_001_bron	BGC_380_pseu	0.290347	0.503607	0.730769	0.705238	0.645161	0.309307	0.192953	21	3	PKSI
BGC_001_bron	BGC_501_hank	0.275978	0.524207	0.710526	0.730458	0.627907	0.289591	0.082419	28	3	PKSI
BGC_243_desu	BGC_382_jing	0.163862	0.699127	1	0.784392	1	0.227515	0.167978	12	3	PKSI
BGC_426_jaco	BGC_455_oryz	0.264724	0.54063	0.921053	0.678536	0.847826	0.338822	0.240462	56	12	PKSI
BGC_046_shan	BGC_180_hirs	0.206432	0.62975	0.965517	0.740683	0.911765	0.266754	0.170076	36	3	PKSI
BGC_046_shan	BGC_342_zhao	0.178177	0.675393	0.9375	0.786494	0.891892	0.219683	0.137324	37	3	PKSI
BGC_148_nami	BGC_458_oryz	0.282466	0.514856	0.724138	0.717964	0.628571	0.296119	0.174064	23	3	PKSI
BGC_180_hirs	BGC_272_spum	0.222628	0.604307	0.848485	0.757676	0.74359	0.249849	0.157049	34	3	PKSI

BGC_1	BGC_2	Raw distance	Squared similarity	Jaccard index	DSS index	Adjacency index	raw DSS non-anchor	raw DSS anchor	Non-anchor domains	Anchor domains	Network
BGC_170_aich	BGC_434_jaco	0.095787	0.817601	1	0.874939	0.962963	0.134869	0.026981	30	3	PKSI
BGC_209_soli	BGC_380_pseu	0.284496	0.511946	0.882353	0.665985	0.761905	0.35268	0.184703	24	3	PKSI
BGC_189_sihw	BGC_294_phth	0.236506	0.582924	0.763158	0.767665	0.608696	0.242611	0.095321	40	3	PKSI
BGC_189_sihw	BGC_209_soli	0.274309	0.526628	0.705882	0.735485	0.571429	0.291678	0.155863	12	3	PKSI
BGC_189_sihw	BGC_317_iter	0.250139	0.562291	0.647059	0.783158	0.615385	0.226486	0.139692	24	3	PKSI
BGC_082_effu	BGC_380_pseu	0.293479	0.499171	0.76	0.689895	0.75	0.322813	0.221147	21	3	PKSI
BGC_382_jing	BGC_434_jaco	0.243567	0.572191	0.657143	0.788633	0.625	0.217433	0.162832	24	3	PKSI
BGC_099_otit	BGC_243_desu	0.172255	0.685162	1	0.773349	1	0.246192	0.148484	12	3	PKSI
BGC_128_rhiz	BGC_243_desu	0.17818	0.675389	1	0.765553	1	0.254221	0.155348	12	3	PKSI
BGC_243_desu	BGC_380_pseu	0.258718	0.549499	0.923077	0.687112	0.8	0.338034	0.212307	12	3	PKSI
BGC_336_insu	BGC_513_para	0.218211	0.611195	0.848485	0.764163	0.717949	0.246997	0.131678	28	3	PKSI
BGC_243_desu	BGC_342_zhao	0.253946	0.556596	0.923077	0.693391	0.8	0.346406	0.147423	12	3	PKSI
BGC_243_desu	BGC_304_alka	0.163743	0.699326	1	0.784549	1	0.224218	0.180382	12	3	PKSI
BGC_036_neof	BGC_220_mala	0.198341	0.642657	0.882353	0.780141	0.731707	0.228145	0.117663	37	3	PKSI
BGC_252_hydr	BGC_294_phth	0.138614	0.741986	0.911765	0.84776	0.825	0.1577	0.084895	37	3	PKSI
BGC_046_shan	BGC_128_rhiz	0.236184	0.583414	0.705882	0.784066	0.631579	0.225093	0.182353	11	3	PKSI
BGC_148_nami	BGC_434_jaco	0.274625	0.526169	0.628571	0.756967	0.589744	0.255188	0.149849	23	3	PKSI
BGC_148_nami	BGC_170_aich	0.293882	0.498603	0.541667	0.758659	0.518519	0.263629	0.152189	12	3	PKSI
BGC_220_mala	BGC_342_zhao	0.248219	0.565174	0.805556	0.737201	0.714286	0.274691	0.108206	39	3	PKSI
BGC_189_sihw	BGC_220_mala	0.299313	0.490962	0.756757	0.687105	0.6	0.328151	0.109477	40	3	PKSI
BGC_180_hirs	BGC_317_iter	0.167995	0.692232	0.933333	0.801259	0.885714	0.207097	0.098463	36	3	PKSI
BGC_036_neof	BGC_189_sihw	0.111962	0.788612	0.852941	0.901173	0.775	0.101994	0.060828	36	3	PKSI
BGC_046_shan	BGC_252_hydr	0.193469	0.650492	0.666667	0.853398	0.564103	0.148591	0.132019	22	3	PKSI
BGC_001_bron	BGC_013_poly	0.259338	0.54858	0.818182	0.721025	0.634146	0.298723	0.088081	29	3	PKSI

BGC_1	BGC_2	Raw distance	Squared similarity	Jaccard index	DSS index	Adjacency index	raw DSS non-anchor	raw DSS anchor	Non-anchor domains	Anchor domains	Network
BGC_243_desu	BGC_513_para	0.167207	0.693543	1	0.77999	1	0.233896	0.164464	12	3	PKSI
BGC_336_insu	BGC_380_pseu	0.291451	0.502042	0.730769	0.703785	0.645161	0.310143	0.198715	21	3	PKSI
BGC_243_desu	BGC_252_hydr	0.190412	0.655433	1	0.749458	1	0.269158	0.176077	12	3	PKSI
BGC_013_poly	BGC_243_desu	0.153869	0.715938	0.923077	0.825071	0.8	0.200245	0.082104	11	3	PKSI
BGC_119_sput	BGC_170_aich	0.102558	0.805402	1	0.86603	0.962963	0.144751	0.026164	30	3	PKSI
BGC_099_otit	BGC_434_jaco	0.065244	0.873769	0.942857	0.933326	0.9	0.069305	0.031599	40	3	PKSI
BGC_119_sput	BGC_243_desu	0.180116	0.672209	1	0.763005	1	0.256277	0.159867	12	3	PKSI
BGC_097_otit	BGC_426_jaco	0.195889	0.646594	0.947368	0.761535	0.846154	0.277389	0.121693	36	12	PKSI
BGC_058_amic	BGC_401_rubr	0.111658	0.789152	1	0.878545	0.923077	0.123833	0.07745	74	4	PKS-NRP_Hybrids
BGC_058_amic	BGC_415_west	0.16118	0.703619	0.862069	0.868837	0.732394	0.130676	0.139795	71	4	PKS-NRP_Hybrids
BGC_401_rubr	BGC_415_west	0.145112	0.730834	0.862069	0.881493	0.760563	0.118322	0.121794	71	4	PKS-NRP_Hybrids
BGC_436_lacu	BGC_481_terr	0.101256	0.80774	0.933333	0.916549	0.835616	0.087183	0.014408	74	4	PKS-NRP_Hybrids
BGC_401_rubr	BGC_415_west	0.200884	0.638586	0.862069	0.881039	0.760563	0.118322	0.121794	71	4	PKSother
BGC_436_lacu	BGC_481_terr	0.135539	0.747292	0.933333	0.925755	0.835616	0.087183	0.014408	74	4	PKSother
BGC_058_amic	BGC_401_rubr	0.089296	0.829382	1	0.884413	0.923077	0.123833	0.07745	74	4	PKSother
BGC_361_amar	BGC_368_pseu	0.146835	0.72789	0.906977	0.830912	0.863636	0.172639	0.156953	41	3	PKSother
BGC_058_amic	BGC_415_west	0.224325	0.601672	0.862069	0.867647	0.732394	0.130676	0.139795	71	4	PKSother
BGC_351_zhao	BGC_399_rubr	0.299709	0.490407	0.833333	0.650644	0.5	0.349356	0	8	0	RiPPs
BGC_334_insu	BGC_495_terr	0.130663	0.755747	1	0.815968	1	0.184032	0	9	0	RiPPs
BGC_107_otit	BGC_231_desu	0.230769	0.591716	0.75	0.780607	0.5	0.219393	0	6	0	RiPPs
BGC_156_nami	BGC_433_jaco	0.271965	0.530035	0.833333	0.685495	0.8	0.314505	0	8	0	RiPPs
BGC_156_nami	BGC_352_amar	0.267025	0.537253	0.833333	0.692454	0.8	0.307546	0	8	0	RiPPs

BGC_1	BGC_2	Raw distance	Squared similarity	Jaccard index	DSS index	Adjacency index	raw DSS non-anchor	raw DSS anchor	Non-anchor domains	Anchor domains	Network
BGC_416_west	BGC_442_lacu	0.203041	0.635144	0.833333	0.782572	0.8	0.217428	0	8	0	RiPPs
BGC_060_amic	BGC_495_terr	0.208774	0.626039	0.833333	0.774497	0.8	0.225503	0	8	0	RiPPs
BGC_179_hirs	BGC_195_soli	0.296851	0.494419	0.833333	0.65467	0.5	0.34533	0	8	0	RiPPs
BGC_060_amic	BGC_352_amar	0.269766	0.533241	0.833333	0.688592	0.8	0.311408	0	8	0	RiPPs
BGC_011_bron	BGC_195_soli	0.249877	0.562684	0.833333	0.72083	0.5	0.27917	0	8	0	RiPPs
BGC_351_zhao	BGC_499_hank	0.294179	0.498183	0.833333	0.658433	0.5	0.341567	0	8	0	RiPPs
BGC_258_croc	BGC_316_iter	0.263787	0.54201	0.833333	0.701239	0.5	0.298761	0	7	0	RiPPs
BGC_011_bron	BGC_086_effu	0.299481	0.490727	0.714286	0.701436	0.25	0.298564	0	8	0	RiPPs
BGC_086_effu	BGC_156_nami	0.272599	0.529112	0.857143	0.678432	0.571429	0.321568	0	9	0	RiPPs
BGC_316_iter	BGC_465_humi	0.296513	0.494894	0.833333	0.65092	0.8	0.34908	0	8	0	RiPPs
BGC_011_bron	BGC_393_jing	0.23675	0.582551	0.833333	0.73932	0.5	0.26068	0	8	0	RiPPs
BGC_393_jing	BGC_399_rubr	0.079263	0.847757	1	0.888362	1	0.111638	0	8	0	RiPPs
BGC_156_nami	BGC_399_rubr	0.02151	0.957443	1	0.969704	1	0.030296	0	8	0	RiPPs
BGC_044_shan	BGC_316_iter	0.177837	0.675951	1	0.749525	1	0.250475	0	8	0	RiPPs
BGC_179_hirs	BGC_393_jing	0.294371	0.497912	0.833333	0.658163	0.5	0.341837	0	8	0	RiPPs
BGC_044_shan	BGC_179_hirs	0.182257	0.668704	1	0.7433	1	0.2567	0	8	0	RiPPs
BGC_156_nami	BGC_195_soli	0.260517	0.546835	0.833333	0.701619	0.8	0.298381	0	8	0	RiPPs
BGC_352_amar	BGC_393_jing	0.262385	0.544076	0.833333	0.698988	0.8	0.301012	0	8	0	RiPPs
BGC_060_amic	BGC_086_effu	0.205607	0.63106	0.857143	0.772786	0.571429	0.227214	0	8	0	RiPPs
BGC_188_sihw	BGC_365_pseu	0.249716	0.562925	0.833333	0.721057	0.5	0.278943	0	7	0	RiPPs
BGC_035_neof	BGC_302_phth	0.116254	0.781007	1	0.836262	1	0.158643	0.209596	9	1	RiPPs
BGC_086_effu	BGC_416_west	0.273857	0.527284	0.857143	0.676661	0.571429	0.323339	0	9	0	RiPPs
BGC_094_otit	BGC_132_rhiz	0.041596	0.918538	1	0.941414	1	0.058586	0	11	0	RiPPs
BGC_044_shan	BGC_442_lacu	0.295312	0.496586	0.833333	0.656838	0.5	0.343162	0	8	0	RiPPs

BGC_1	BGC_2	Raw distance	Squared similarity	Jaccard index	DSS index	Adjacency index	raw DSS non-anchor	raw DSS anchor	Non-anchor domains	Anchor domains	Network
BGC_044_shan	BGC_305_alka	0.29876	0.491738	0.833333	0.651981	0.5	0.348019	0	8	0	RiPPs
BGC_218_mala	BGC_465_humi	0.247032	0.566961	0.857143	0.710753	0.833333	0.289247	0	9	0	RiPPs
BGC_132_rhiz	BGC_462_oryz	0.037257	0.926875	1	0.947526	1	0.052474	0	11	0	RiPPs
BGC_011_bron	BGC_365_pseu	0.26508	0.540108	0.833333	0.699418	0.5	0.300582	0	8	0	RiPPs
BGC_023_poly	BGC_113_sput	0.114605	0.783924	1	0.838584	1	0.161416	0	14	0	RiPPs
BGC_334_insu	BGC_351_zhao	0.234563	0.585893	0.833333	0.742399	0.5	0.257601	0	7	0	RiPPs
BGC_107_otit	BGC_416_west	0.268958	0.534423	0.833333	0.689731	0.8	0.310269	0	8	0	RiPPs
BGC_365_pseu	BGC_399_rubr	0.261372	0.545571	0.833333	0.700415	0.8	0.299585	0	8	0	RiPPs
BGC_499_hank	BGC_515_para	0.180481	0.671611	1	0.745801	1	0.254199	0	8	0	RiPPs
BGC_107_otit	BGC_442_lacu	0.260525	0.546823	0.833333	0.701608	0.8	0.298392	0	8	0	RiPPs
BGC_060_amic	BGC_107_otit	0.266162	0.538518	0.833333	0.693668	0.8	0.306332	0	8	0	RiPPs
BGC_060_amic	BGC_530_aspl	0.23218	0.589548	0.857143	0.73536	0.571429	0.26464	0	8	0	RiPPs
BGC_086_effu	BGC_530_aspl	0.119978	0.774439	1	0.831017	1	0.168983	0	9	0	RiPPs
BGC_352_amar	BGC_416_west	0.266694	0.537738	0.833333	0.692919	0.8	0.307081	0	8	0	RiPPs
BGC_156_nami	BGC_316_iter	0.236249	0.583316	0.833333	0.740025	0.5	0.259975	0	7	0	RiPPs
BGC_011_bron	BGC_495_terr	0.245552	0.569191	0.833333	0.726922	0.5	0.273078	0	8	0	RiPPs
BGC_011_bron	BGC_060_amic	0.237413	0.581538	0.833333	0.738385	0.5	0.261615	0	8	0	RiPPs
BGC_168_aich	BGC_433_jaco	0.044031	0.913877	1	0.937985	1	0.062015	0	8	0	RiPPs
BGC_305_alka	BGC_365_pseu	0.259801	0.547895	0.833333	0.702628	0.8	0.297372	0	8	0	RiPPs
BGC_168_aich	BGC_399_rubr	0.267233	0.536947	0.833333	0.69216	0.8	0.30784	0	8	0	RiPPs
BGC_258_croc	BGC_393_jing	0.294054	0.49836	0.833333	0.654385	0.8	0.345615	0	8	0	RiPPs
BGC_365_pseu	BGC_442_lacu	0.262789	0.54348	0.833333	0.698419	0.8	0.301581	0	8	0	RiPPs
BGC_156_nami	BGC_305_alka	0.030392	0.94014	1	0.957195	1	0.042806	0	8	0	RiPPs
BGC_060_amic	BGC_393_jing	0.084777	0.837632	1	0.880595	1	0.119405	0	8	0	RiPPs

BGC_1	BGC_2	Raw distance	Squared similarity	Jaccard index	DSS index	Adjacency index	raw DSS non-anchor	raw DSS anchor	Non-anchor domains	Anchor domains	Network
BGC_107_otit	BGC_365_pseu	0.178504	0.674856	1	0.748586	1	0.251414	0	8	0	RiPPs
BGC_305_alka	BGC_352_amar	0.264075	0.541585	0.833333	0.696608	0.8	0.303392	0	8	0	RiPPs
BGC_416_west	BGC_499_hank	0.136232	0.746096	1	0.808124	1	0.191876	0	8	0	RiPPs
BGC_035_neof	BGC_107_otit	0.241171	0.575821	0.833333	0.733092	0.5	0.266908	0	7	0	RiPPs
BGC_276_spum	BGC_365_pseu	0.256224	0.553203	0.833333	0.711891	0.5	0.288109	0	7	0	RiPPs
BGC_365_pseu	BGC_433_jaco	0.17778	0.676046	1	0.749606	1	0.250394	0	8	0	RiPPs
BGC_393_jing	BGC_433_jaco	0.26658	0.537906	0.833333	0.69308	0.8	0.30692	0	8	0	RiPPs
BGC_107_otit	BGC_334_insu	0.269833	0.533144	0.833333	0.688499	0.8	0.311501	0	8	0	RiPPs
BGC_107_otit	BGC_495_terr	0.265217	0.539906	0.833333	0.695	0.8	0.305	0	8	0	RiPPs
BGC_107_otit	BGC_393_jing	0.263566	0.542335	0.833333	0.697325	0.8	0.302675	0	8	0	RiPPs
BGC_011_bron	BGC_334_insu	0.238765	0.579478	0.833333	0.736481	0.5	0.263519	0	8	0	RiPPs
BGC_156_nami	BGC_499_hank	0.136602	0.745456	1	0.807603	1	0.192397	0	8	0	RiPPs
BGC_195_soli	BGC_442_lacu	0.144196	0.732401	1	0.796908	1	0.203092	0	9	0	RiPPs
BGC_179_hirs	BGC_334_insu	0.299418	0.490816	0.833333	0.651055	0.5	0.348945	0	8	0	RiPPs
BGC_060_amic	BGC_316_iter	0.228562	0.595116	0.833333	0.750852	0.5	0.249148	0	7	0	RiPPs
BGC_060_amic	BGC_179_hirs	0.233947	0.586838	0.833333	0.743268	0.5	0.256732	0	7	0	RiPPs
BGC_305_alka	BGC_316_iter	0.233408	0.587663	0.833333	0.744026	0.5	0.255974	0	7	0	RiPPs
BGC_258_croc	BGC_351_zhao	0.25208	0.559384	0.833333	0.717728	0.5	0.282272	0	7	0	RiPPs
BGC_156_nami	BGC_168_aich	0.270816	0.531709	0.833333	0.687113	0.8	0.312887	0	8	0	RiPPs
BGC_365_pseu	BGC_499_hank	0.273984	0.527099	0.833333	0.682651	0.8	0.317349	0	8	0	RiPPs
BGC_305_alka	BGC_334_insu	0.24385	0.571763	0.833333	0.725094	0.8	0.274906	0	8	0	RiPPs
BGC_365_pseu	BGC_393_jing	0.259783	0.547921	0.833333	0.702652	0.8	0.297348	0	8	0	RiPPs
BGC_060_amic	BGC_442_lacu	0.208917	0.625813	0.833333	0.774296	0.8	0.225704	0	8	0	RiPPs
BGC_060_amic	BGC_305_alka	0.039739	0.922101	1	0.94403	1	0.05597	0	8	0	RiPPs

BGC_1	BGC_2	Raw distance	Squared similarity	Jaccard index	DSS index	Adjacency index	raw DSS non-anchor	raw DSS anchor	Non-anchor domains	Anchor domains	Network
BGC_060_amic	BGC_416_west	0.042577	0.916659	1	0.940032	1	0.059968	0	8	0	RiPPs
BGC_305_alka	BGC_442_lacu	0.201711	0.637266	0.833333	0.784445	0.8	0.215555	0	8	0	RiPPs
BGC_035_neof	BGC_188_sihw	0.078353	0.849434	1	0.889644	1	0.102927	0.177215	9	1	RiPPs
BGC_179_hirs	BGC_351_zhao	0.178366	0.675083	1	0.748781	1	0.251219	0	8	0	RiPPs
BGC_305_alka	BGC_416_west	0.033821	0.933501	1	0.952364	1	0.047636	0	8	0	RiPPs
BGC_023_poly	BGC_429_jaco	0.140824	0.738184	0.923077	0.835011	0.785714	0.164989	0	14	0	RiPPs
BGC_258_croc	BGC_499_hank	0.299428	0.490801	0.833333	0.646815	0.8	0.353185	0	8	0	RiPPs
BGC_316_iter	BGC_393_jing	0.297904	0.492938	0.833333	0.653186	0.5	0.346814	0	8	0	RiPPs
BGC_168_aich	BGC_393_jing	0.26286	0.543376	0.833333	0.698319	0.8	0.301681	0	8	0	RiPPs
BGC_044_shan	BGC_351_zhao	0.253754	0.556883	0.875	0.697178	0.625	0.302822	0	10	0	RiPPs
BGC_393_jing	BGC_495_terr	0.18401	0.66584	0.833333	0.809376	0.8	0.190624	0	8	0	RiPPs
BGC_094_otit	BGC_113_sput	0.05725	0.888777	0.916667	0.95548	0.769231	0.04452	0	13	0	RiPPs
BGC_305_alka	BGC_433_jaco	0.268541	0.535032	0.833333	0.690318	0.8	0.309682	0	8	0	RiPPs
BGC_132_rhiz	BGC_429_jaco	0.037261	0.926867	1	0.94752	1	0.05248	0	11	0	RiPPs
BGC_393_jing	BGC_515_para	0.145901	0.729485	1	0.794506	1	0.205494	0	8	0	RiPPs
BGC_246_hydr	BGC_352_amar	0.243734	0.571938	0.833333	0.729483	0.5	0.270517	0	7	0	RiPPs
BGC_044_shan	BGC_156_nami	0.296363	0.495104	0.833333	0.655357	0.5	0.344643	0	8	0	RiPPs
BGC_168_aich	BGC_305_alka	0.26737	0.536746	0.833333	0.691967	0.8	0.308033	0	8	0	RiPPs
BGC_399_rubr	BGC_495_terr	0.204948	0.632108	0.833333	0.779886	0.8	0.220114	0	8	0	RiPPs
BGC_168_aich	BGC_416_west	0.269967	0.532948	0.833333	0.68831	0.8	0.31169	0	8	0	RiPPs
BGC_416_west	BGC_515_para	0.120034	0.774341	1	0.830938	1	0.169062	0	8	0	RiPPs
BGC_433_jaco	BGC_442_lacu	0.259477	0.548374	0.833333	0.703083	0.8	0.296917	0	8	0	RiPPs
BGC_044_shan	BGC_499_hank	0.288799	0.505807	0.833333	0.666011	0.5	0.333989	0	8	0	RiPPs
BGC_316_iter	BGC_499_hank	0.295877	0.495789	0.833333	0.656041	0.5	0.343959	0	8	0	RiPPs

BGC_1	BGC_2	Raw distance	Squared similarity	Jaccard index	DSS index	Adjacency index	raw DSS non-anchor	raw DSS anchor	Non-anchor domains	Anchor domains	Network
BGC_258_croc	BGC_442_lacu	0.299134	0.491213	0.833333	0.647229	0.8	0.352771	0	8	0	RiPPs
BGC_044_shan	BGC_393_jing	0.296696	0.494636	0.833333	0.654888	0.5	0.345112	0	8	0	RiPPs
BGC_035_neof	BGC_168_aich	0.251735	0.5599	0.833333	0.718213	0.5	0.281787	0	7	0	RiPPs
BGC_433_jaco	BGC_515_para	0.294853	0.497232	0.833333	0.653259	0.8	0.346741	0	8	0	RiPPs
BGC_246_hydr	BGC_365_pseu	0.25268	0.558487	0.833333	0.716883	0.5	0.283117	0	7	0	RiPPs
BGC_168_aich	BGC_515_para	0.294871	0.497207	0.833333	0.653234	0.8	0.346766	0	8	0	RiPPs
BGC_352_amar	BGC_495_terr	0.268039	0.535767	0.833333	0.691025	0.8	0.308975	0	8	0	RiPPs
BGC_168_aich	BGC_499_hank	0.275379	0.525076	0.833333	0.680687	0.8	0.319313	0	8	0	RiPPs
BGC_416_west	BGC_530_aspl	0.230705	0.591815	0.857143	0.737438	0.571429	0.262562	0	8	0	RiPPs
BGC_246_hydr	BGC_433_jaco	0.268012	0.535806	0.833333	0.695288	0.5	0.304712	0	7	0	RiPPs
BGC_107_otit	BGC_515_para	0.294542	0.497671	0.833333	0.653697	0.8	0.346303	0	8	0	RiPPs
BGC_393_jing	BGC_442_lacu	0.182487	0.668327	0.833333	0.81152	0.8	0.18848	0	8	0	RiPPs
BGC_393_jing	BGC_416_west	0.081094	0.844388	1	0.885783	1	0.114217	0	8	0	RiPPs
BGC_276_spum	BGC_433_jaco	0.256334	0.553039	0.833333	0.711736	0.5	0.288264	0	7	0	RiPPs
BGC_156_nami	BGC_530_aspl	0.22797	0.59603	0.857143	0.741289	0.571429	0.258711	0	8	0	RiPPs
BGC_035_neof	BGC_365_pseu	0.260173	0.547343	0.833333	0.706329	0.5	0.293671	0	7	0	RiPPs
BGC_305_alka	BGC_515_para	0.124171	0.767076	1	0.825111	1	0.174889	0	8	0	RiPPs
BGC_188_sihw	BGC_352_amar	0.236904	0.582315	0.833333	0.739102	0.5	0.260898	0	7	0	RiPPs
BGC_044_shan	BGC_302_phth	0.248778	0.564335	0.857143	0.708294	0.833333	0.291706	0	9	0	RiPPs
BGC_086_effu	BGC_399_rubr	0.268993	0.534371	0.857143	0.683511	0.571429	0.316489	0	9	0	RiPPs
BGC_393_jing	BGC_499_hank	0.134542	0.749017	1	0.810504	1	0.189496	0	8	0	RiPPs
BGC_442_lacu	BGC_495_terr	0.019692	0.961004	1	0.972265	1	0.027735	0	9	0	RiPPs
BGC_302_phth	BGC_352_amar	0.249644	0.563034	0.833333	0.721159	0.5	0.278841	0	7	0	RiPPs
BGC_060_amic	BGC_433_jaco	0.267449	0.536631	0.833333	0.691856	0.8	0.308144	0	8	0	RiPPs

BGC_1	BGC_2	Raw distance	Squared similarity	Jaccard index	DSS index	Adjacency index	raw DSS non-anchor	raw DSS anchor	Non-anchor domains	Anchor domains	Network
BGC_365_pseu	BGC_416_west	0.25962	0.548163	0.833333	0.702883	0.8	0.297117	0	8	0	RiPPs
BGC_060_amic	BGC_195_soli	0.253942	0.556603	0.833333	0.710881	0.8	0.289119	0	8	0	RiPPs
BGC_011_bron	BGC_515_para	0.2921	0.501122	0.833333	0.661361	0.5	0.338639	0	8	0	RiPPs
BGC_011_bron	BGC_305_alka	0.237065	0.58207	0.833333	0.738876	0.5	0.261124	0	8	0	RiPPs
BGC_113_sput	BGC_132_rhiz	0.037217	0.926951	1	0.947582	1	0.052418	0	11	0	RiPPs
BGC_218_mala	BGC_352_amar	0.251931	0.559608	0.833333	0.717938	0.5	0.282062	0	7	0	RiPPs
BGC_305_alka	BGC_393_jing	0.079927	0.846534	1	0.887426	1	0.112574	0	8	0	RiPPs
BGC_107_otit	BGC_123_rhiz	0.226344	0.598544	0.75	0.78684	0.5	0.21316	0	6	0	RiPPs
BGC_305_alka	BGC_495_terr	0.204846	0.63227	0.833333	0.780029	0.8	0.219971	0	8	0	RiPPs
BGC_399_rubr	BGC_416_west	0.039295	0.922954	1	0.944655	1	0.055345	0	8	0	RiPPs
BGC_023_poly	BGC_462_oryz	0.108673	0.794464	0.916667	0.883054	0.769231	0.116946	0	14	0	RiPPs
BGC_393_jing	BGC_530_aspl	0.233575	0.587408	0.857143	0.733396	0.571429	0.266604	0	8	0	RiPPs
BGC_113_sput	BGC_429_jaco	0.035814	0.929655	0.923077	0.982912	0.785714	0.017088	0	14	0	RiPPs
BGC_060_amic	BGC_499_hank	0.138962	0.741387	1	0.804279	1	0.195721	0	8	0	RiPPs
BGC_179_hirs	BGC_316_iter	0.135554	0.747267	1	0.809079	1	0.190921	0	8	0	RiPPs
BGC_218_mala	BGC_365_pseu	0.245582	0.569146	0.833333	0.72688	0.5	0.27312	0	7	0	RiPPs
BGC_188_sihw	BGC_433_jaco	0.242303	0.574105	0.833333	0.731498	0.5	0.268502	0	7	0	RiPPs
BGC_168_aich	BGC_195_soli	0.262514	0.543885	0.833333	0.698806	0.8	0.301194	0	8	0	RiPPs
BGC_334_insu	BGC_442_lacu	0.136751	0.745198	1	0.807393	1	0.192607	0	9	0	RiPPs
BGC_044_shan	BGC_246_hydr	0.248031	0.565457	0.857143	0.709346	0.833333	0.290654	0	9	0	RiPPs
BGC_433_jaco	BGC_495_terr	0.265054	0.540146	0.833333	0.695229	0.8	0.304771	0	8	0	RiPPs
BGC_113_sput	BGC_462_oryz	0.134717	0.748715	0.916667	0.846372	0.769231	0.153628	0	14	0	RiPPs
BGC_276_spum	BGC_352_amar	0.25626	0.55315	0.833333	0.711841	0.5	0.288159	0	7	0	RiPPs
BGC_302_phth	BGC_433_jaco	0.265759	0.53911	0.833333	0.698462	0.5	0.301538	0	7	0	RiPPs

BGC_1	BGC_2	Raw distance	Squared similarity	Jaccard index	DSS index	Adjacency index	raw DSS non-anchor	raw DSS anchor	Non-anchor domains	Anchor domains	Network
BGC_156_nami	BGC_351_zhao	0.236968	0.582218	0.833333	0.739013	0.5	0.260987	0	7	0	RiPPs
BGC_305_alka	BGC_499_hank	0.135815	0.746816	1	0.808711	1	0.191289	0	8	0	RiPPs
BGC_365_pseu	BGC_495_terr	0.267596	0.536416	0.833333	0.691649	0.8	0.308351	0	8	0	RiPPs
BGC_060_amic	BGC_351_zhao	0.233151	0.588057	0.833333	0.744388	0.5	0.255612	0	7	0	RiPPs
BGC_011_bron	BGC_399_rubr	0.236659	0.582689	0.833333	0.739447	0.5	0.260553	0	8	0	RiPPs
BGC_156_nami	BGC_393_jing	0.084336	0.838441	1	0.881217	1	0.118783	0	8	0	RiPPs
BGC_060_amic	BGC_156_nami	0.045555	0.910964	1	0.935837	1	0.064163	0	8	0	RiPPs
BGC_258_croc	BGC_334_insu	0.290945	0.502759	0.833333	0.658763	0.8	0.341237	0	8	0	RiPPs
BGC_011_bron	BGC_499_hank	0.249775	0.562837	0.833333	0.720974	0.5	0.279026	0	8	0	RiPPs
BGC_156_nami	BGC_334_insu	0.246024	0.56848	0.833333	0.722032	0.8	0.277968	0	8	0	RiPPs
BGC_188_sihw	BGC_302_phth	0.117884	0.778129	1	0.833966	1	0.157057	0.246819	9	1	RiPPs
BGC_094_otit	BGC_462_oryz	0.067341	0.869852	1	0.905153	1	0.094847	0	13	0	RiPPs
BGC_035_neof	BGC_352_amar	0.24747	0.566302	0.833333	0.724221	0.5	0.275779	0	7	0	RiPPs
BGC_305_alka	BGC_351_zhao	0.235924	0.583813	0.833333	0.740483	0.5	0.259517	0	7	0	RiPPs
BGC_499_hank	BGC_530_aspl	0.2152	0.615911	0.857143	0.759276	0.571429	0.240724	0	8	0	RiPPs
BGC_060_amic	BGC_334_insu	0.243816	0.571814	0.833333	0.725142	0.8	0.274858	0	8	0	RiPPs
BGC_168_aich	BGC_352_amar	0.18044	0.671678	1	0.745859	1	0.254141	0	8	0	RiPPs
BGC_060_amic	BGC_515_para	0.117509	0.77879	1	0.834494	1	0.165506	0	8	0	RiPPs
BGC_433_jaco	BGC_499_hank	0.277557	0.521923	0.833333	0.677619	0.8	0.322381	0	8	0	RiPPs
BGC_060_amic	BGC_399_rubr	0.040899	0.919874	1	0.942396	1	0.057604	0	8	0	RiPPs
BGC_011_bron	BGC_168_aich	0.260863	0.546323	0.833333	0.705357	0.5	0.294643	0	8	0	RiPPs
BGC_399_rubr	BGC_499_hank	0.135679	0.747051	1	0.808903	1	0.191097	0	8	0	RiPPs
BGC_107_otit	BGC_168_aich	0.025248	0.950142	1	0.96444	1	0.03556	0	8	0	RiPPs
BGC_035_neof	BGC_246_hydr	0.119475	0.775324	1	0.831725	1	0.155305	0.285	9	1	RiPPs

BGC_1	BGC_2	Raw distance	Squared similarity	Jaccard index	DSS index	Adjacency index	raw DSS non-anchor	raw DSS anchor	Non-anchor domains	Anchor domains	Network
BGC_060_amic	BGC_365_pseu	0.262661	0.543669	0.833333	0.6986	0.8	0.3014	0	8	0	RiPPs
BGC_218_mala	BGC_276_spum	0.104097	0.802643	1	0.853385	1	0.146615	0	9	0	RiPPs
BGC_011_bron	BGC_433_jaco	0.256549	0.55272	0.833333	0.711434	0.5	0.288566	0	8	0	RiPPs
BGC_334_insu	BGC_499_hank	0.266821	0.537552	0.833333	0.694618	0.666667	0.305382	0	9	0	RiPPs
BGC_168_aich	BGC_231_desu	0.229452	0.593744	0.75	0.782462	0.5	0.217538	0	6	0	RiPPs
BGC_107_otit	BGC_499_hank	0.276741	0.523104	0.833333	0.678769	0.8	0.321231	0	8	0	RiPPs
BGC_044_shan	BGC_060_amic	0.293402	0.499281	0.833333	0.659528	0.5	0.340472	0	8	0	RiPPs
BGC_168_aich	BGC_495_terr	0.26592	0.538873	0.833333	0.694009	0.8	0.305991	0	8	0	RiPPs
BGC_399_rubr	BGC_433_jaco	0.267122	0.537111	0.833333	0.692317	0.8	0.307683	0	8	0	RiPPs
BGC_156_nami	BGC_495_terr	0.206349	0.629882	0.833333	0.777913	0.8	0.222087	0	8	0	RiPPs
BGC_168_aich	BGC_334_insu	0.267216	0.536973	0.833333	0.692184	0.8	0.307816	0	8	0	RiPPs
BGC_316_iter	BGC_351_zhao	0.175291	0.680144	1	0.753111	1	0.246889	0	8	0	RiPPs
BGC_156_nami	BGC_416_west	0.037901	0.925635	1	0.946619	1	0.053381	0	8	0	RiPPs
BGC_123_rhiz	BGC_231_desu	0.168642	0.691157	1	0.762476	1	0.237524	0	8	0	RiPPs
BGC_246_hydr	BGC_302_phth	0.101259	0.807736	1	0.857382	1	0.128161	0.272727	9	1	RiPPs
BGC_352_amar	BGC_365_pseu	0.062234	0.879405	1	0.912346	1	0.087653	0	8	0	RiPPs
BGC_351_zhao	BGC_442_lacu	0.299866	0.490187	0.833333	0.650423	0.5	0.349577	0	8	0	RiPPs
BGC_168_aich	BGC_365_pseu	0.172856	0.684167	1	0.756541	1	0.243459	0	8	0	RiPPs
BGC_156_nami	BGC_365_pseu	0.260275	0.547193	0.833333	0.70196	0.8	0.29804	0	8	0	RiPPs
BGC_416_west	BGC_495_terr	0.29807	0.492705	0.714286	0.697553	0.666667	0.302447	0	9	0	RiPPs
BGC_107_otit	BGC_195_soli	0.260685	0.546587	0.833333	0.701383	0.8	0.298617	0	8	0	RiPPs
BGC_218_mala	BGC_433_jaco	0.258686	0.549546	0.833333	0.708423	0.5	0.291577	0	7	0	RiPPs
BGC_107_otit	BGC_305_alka	0.268595	0.534953	0.833333	0.690241	0.8	0.309759	0	8	0	RiPPs
BGC_060_amic	BGC_168_aich	0.266272	0.538357	0.833333	0.693514	0.8	0.306486	0	8	0	RiPPs

BGC_1	BGC_2	Raw distance	Squared similarity	Jaccard index	DSS index	Adjacency index	raw DSS non-anchor	raw DSS anchor	Non-anchor domains	Anchor domains	Network
BGC_316_iter	BGC_399_rubr	0.29907	0.491302	0.833333	0.651544	0.5	0.348456	0	8	0	RiPPs
BGC_156_nami	BGC_179_hirs	0.241932	0.574667	0.833333	0.73202	0.5	0.26798	0	7	0	RiPPs
BGC_258_croc	BGC_495_terr	0.290845	0.502901	0.833333	0.658904	0.8	0.341096	0	8	0	RiPPs
BGC_107_otit	BGC_399_rubr	0.268458	0.535153	0.833333	0.690434	0.8	0.309566	0	8	0	RiPPs
BGC_515_para	BGC_530_aspl	0.261739	0.545029	0.857143	0.693728	0.571429	0.306272	0	8	0	RiPPs
BGC_086_effu	BGC_305_alka	0.269968	0.532947	0.857143	0.682138	0.571429	0.317862	0	9	0	RiPPs
BGC_399_rubr	BGC_530_aspl	0.230536	0.592075	0.857143	0.737676	0.571429	0.262324	0	8	0	RiPPs
BGC_188_sihw	BGC_246_hydr	0.118006	0.777914	1	0.833795	1	0.151761	0.296203	9	1	RiPPs
BGC_429_jaco	BGC_462_oryz	0.19788	0.643397	0.846154	0.785992	0.714286	0.214008	0	15	0	RiPPs
BGC_156_nami	BGC_442_lacu	0.202891	0.635384	0.833333	0.782783	0.8	0.217217	0	8	0	RiPPs
BGC_352_amar	BGC_433_jaco	0.18292	0.66762	1	0.742367	1	0.257633	0	8	0	RiPPs
BGC_107_otit	BGC_156_nami	0.272696	0.52897	0.833333	0.684465	0.8	0.315535	0	8	0	RiPPs
BGC_044_shan	BGC_334_insu	0.292438	0.500643	0.833333	0.660885	0.5	0.339115	0	8	0	RiPPs
BGC_305_alka	BGC_530_aspl	0.230879	0.591547	0.857143	0.737192	0.571429	0.262808	0	8	0	RiPPs
BGC_218_mala	BGC_316_iter	0.29557	0.496222	0.833333	0.652249	0.8	0.347751	0	8	0	RiPPs
BGC_044_shan	BGC_399_rubr	0.293935	0.498528	0.833333	0.658777	0.5	0.341223	0	8	0	RiPPs
BGC_351_zhao	BGC_393_jing	0.294399	0.497873	0.833333	0.658124	0.5	0.341876	0	8	0	RiPPs
BGC_094_otit	BGC_429_jaco	0.080847	0.844843	0.846154	0.950827	0.714286	0.049173	0	13	0	RiPPs
BGC_011_bron	BGC_258_croc	0.299798	0.490283	0.833333	0.65052	0.5	0.34948	0	8	0	RiPPs
BGC_352_amar	BGC_442_lacu	0.262999	0.543171	0.833333	0.698124	0.8	0.301876	0	8	0	RiPPs
BGC_399_rubr	BGC_515_para	0.124646	0.766244	1	0.824442	1	0.175558	0	8	0	RiPPs
BGC_107_otit	BGC_352_amar	0.176491	0.678167	1	0.751421	1	0.248579	0	8	0	RiPPs
BGC_011_bron	BGC_352_amar	0.266716	0.537705	0.833333	0.697114	0.5	0.302886	0	8	0	RiPPs
BGC_035_neof	BGC_433_jaco	0.250738	0.561393	0.833333	0.719618	0.5	0.280382	0	7	0	RiPPs

BGC_1	BGC_2	Raw distance	Squared similarity	Jaccard index	DSS index	Adjacency index	raw DSS non-anchor	raw DSS anchor	Non-anchor domains	Anchor domains	Network
BGC_352_amar	BGC_499_hank	0.272251	0.529619	0.833333	0.685093	0.8	0.314907	0	8	0	RiPPs
BGC_011_bron	BGC_442_lacu	0.243287	0.572615	0.833333	0.730113	0.5	0.269887	0	8	0	RiPPs
BGC_276_spum	BGC_465_humi	0.258177	0.550301	0.857143	0.695056	0.833333	0.304944	0	9	0	RiPPs
BGC_195_soli	BGC_334_insu	0.135163	0.747944	1	0.80963	1	0.19037	0	9	0	RiPPs
BGC_011_bron	BGC_156_nami	0.244651	0.570552	0.833333	0.728191	0.5	0.271809	0	8	0	RiPPs
BGC_044_shan	BGC_188_sihw	0.218667	0.610482	0.875	0.743326	0.857143	0.256674	0	10	0	RiPPs
BGC_195_soli	BGC_495_terr	0.243069	0.572945	0.857143	0.715999	0.857143	0.284001	0	10	0	RiPPs
BGC_011_bron	BGC_107_otit	0.260834	0.546366	0.833333	0.705397	0.5	0.294603	0	8	0	RiPPs
BGC_399_rubr	BGC_442_lacu	0.200827	0.638677	0.833333	0.785689	0.8	0.214311	0	8	0	RiPPs
BGC_195_soli	BGC_316_iter	0.231765	0.590185	0.833333	0.746341	0.5	0.25366	0	7	0	RiPPs
BGC_086_effu	BGC_393_jing	0.268405	0.535231	0.857143	0.684339	0.571429	0.315661	0	9	0	RiPPs
BGC_086_effu	BGC_499_hank	0.268584	0.534969	0.857143	0.684086	0.571429	0.315914	0	9	0	RiPPs
BGC_023_poly	BGC_094_otit	0.145975	0.729358	0.916667	0.830515	0.769231	0.169485	0	14	0	RiPPs
BGC_156_nami	BGC_515_para	0.121304	0.772106	1	0.829149	1	0.170851	0	8	0	RiPPs
BGC_107_otit	BGC_433_jaco	0.045415	0.911232	1	0.936035	1	0.063965	0	8	0	RiPPs
BGC_352_amar	BGC_399_rubr	0.267335	0.536797	0.833333	0.692016	0.8	0.307984	0	8	0	RiPPs
BGC_305_alka	BGC_399_rubr	0.020818	0.958798	1	0.970679	1	0.029321	0	8	0	RiPPs
BGC_011_bron	BGC_416_west	0.246832	0.567263	0.833333	0.72512	0.5	0.27488	0	8	0	RiPPs
BGC_023_poly	BGC_132_rhiz	0.036454	0.92842	1	0.948656	1	0.051344	0	11	0	RiPPs
BGC_168_aich	BGC_442_lacu	0.260567	0.546761	0.833333	0.701549	0.8	0.298451	0	8	0	RiPPs
BGC_416_west	BGC_433_jaco	0.27007	0.532798	0.833333	0.688164	0.8	0.311836	0	8	0	RiPPs
BGC_302_phth	BGC_365_pseu	0.26058	0.546743	0.833333	0.705756	0.5	0.294244	0	7	0	RiPPs
BGC_171_hirs	BGC_244_hydr	0.269613	0.533465	1	0.640516	1	0.356443	0.371648	8	1	Terpene
BGC_359_amar	BGC_376_pseu	0.226362	0.598516	0.866667	0.743264	0.857143	0.266281	0.189922	14	1	Terpene

BGC_1	BGC_2	Raw distance	Squared similarity	Jaccard index	DSS index	Adjacency index	raw DSS non-anchor	raw DSS anchor	Non-anchor domains	Anchor domains	Network
BGC_062_amic	BGC_516_para	0.202733	0.635634	0.882353	0.775876	0.777778	0.239415	0.101796	16	1	Terpene
BGC_387_jing	BGC_454_oryz	0.155452	0.713262	0.933333	0.822273	0.823529	0.165598	0.274752	16	1	Terpene
BGC_312_alka	BGC_405_rubr	0.146103	0.72914	0.882353	0.850604	0.789474	0.159333	0.055	19	1	Terpene
BGC_392_jing	BGC_494_terr	0.2501	0.562351	0.928571	0.698915	0.8	0.285874	0.407563	14	1	Terpene
BGC_050_amic	BGC_410_west	0.214905	0.616374	0.714286	0.821023	0.529412	0.178977	0	15	0	Terpene
BGC_050_amic	BGC_485_terr	0.194854	0.64826	0.833333	0.803687	0.714286	0.196313	0	15	0	Terpene
BGC_145_nami	BGC_312_alka	0.170218	0.688538	0.736842	0.870491	0.590909	0.136141	0.069825	18	1	Terpene
BGC_398_rubr	BGC_417_west	0.113571	0.785756	0.875	0.894405	0.8125	0.113668	0.04908	14	1	Terpene
BGC_244_hydr	BGC_475_humi	0.260228	0.547263	0.842105	0.717357	0.666667	0.270194	0.388462	17	1	Terpene
BGC_145_nami	BGC_508_hank	0.158643	0.707881	0.833333	0.843177	0.846154	0.156823	0	13	0	Terpene
BGC_138_rhiz	BGC_508_hank	0.226544	0.598234	0.785714	0.775918	0.6875	0.224082	0	16	0	Terpene
BGC_157_nami	BGC_306_alka	0.114043	0.784919	0.882353	0.89413	0.777778	0.116037	0.02454	16	1	Terpene
BGC_138_rhiz	BGC_405_rubr	0.288101	0.506801	0.7	0.724851	0.565217	0.280968	0.222772	18	1	Terpene
BGC_183_sihw	BGC_297_phth	0.273325	0.528056	0.863636	0.700135	0.576923	0.304791	0.248139	21	1	Terpene
BGC_357_amar	BGC_374_pseu	0.205591	0.631086	0.88	0.773565	0.764706	0.220678	0.321429	33	1	Terpene
BGC_030_neof	BGC_299_phth	0.26421	0.541387	0.75	0.742958	0.571429	0.259479	0.237548	16	1	Terpene
BGC_424_jaco	BGC_508_hank	0.141974	0.736209	0.916667	0.838052	0.923077	0.161948	0	14	0	Terpene
BGC_062_amic	BGC_417_west	0.113788	0.785372	0.875	0.894116	0.8125	0.108962	0.084337	14	1	Terpene
BGC_312_alka	BGC_508_hank	0.263629	0.542242	0.769231	0.732255	0.666667	0.267745	0	15	0	Terpene
BGC_184_sihw	BGC_282_spum	0.248138	0.565297	0.833333	0.738991	0.619048	0.250999	0.341085	16	1	Terpene
BGC_145_nami	BGC_410_west	0.203785	0.633958	0.684211	0.8428	0.545455	0.16893	0.0575	17	1	Terpene
BGC_112_sput	BGC_432_jaco	0.243249	0.572672	0.75	0.764557	0.666667	0.255341	0.046414	19	1	Terpene
BGC_443_lacu	BGC_508_hank	0.15369	0.716241	0.916667	0.822431	0.923077	0.177569	0	14	0	Terpene
BGC_346_zhao	BGC_508_hank	0.220011	0.608383	0.75	0.797128	0.642857	0.202872	0	12	0	Terpene

BGC_1	BGC_2	Raw distance	Squared similarity	Jaccard index	DSS index	Adjacency index	raw DSS non-anchor	raw DSS anchor	Non-anchor domains	Anchor domains	Network
BGC_062_amic	BGC_157_nami	0.157758	0.709372	0.882353	0.835844	0.777778	0.170369	0.114458	16	1	Terpene
BGC_157_nami	BGC_398_rubr	0.131012	0.755141	0.882353	0.871505	0.777778	0.141489	0.02454	16	1	Terpene
BGC_050_amic	BGC_145_nami	0.106465	0.798404	0.916667	0.894554	0.785714	0.105446	0	15	0	Terpene
BGC_183_sihw	BGC_253_hydr	0.244821	0.570296	0.909091	0.718328	0.692308	0.262142	0.506266	23	1	Terpene
BGC_050_amic	BGC_138_rhiz	0.219392	0.609349	0.846154	0.766281	0.733333	0.233719	0	15	0	Terpene
BGC_145_nami	BGC_509_para	0.073328	0.858721	1	0.902229	1	0.094745	0.125	18	1	Terpene
BGC_030_neof	BGC_326_insu	0.294451	0.497799	0.941176	0.634849	0.823529	0.357133	0.425287	15	1	Terpene
BGC_485_terr	BGC_508_hank	0.147688	0.726436	0.916667	0.830433	0.923077	0.169567	0	14	0	Terpene
BGC_253_hydr	BGC_297_phth	0.180129	0.672188	0.952381	0.781617	0.863636	0.198741	0.424623	21	1	Terpene
BGC_312_alka	BGC_410_west	0.222692	0.604208	0.736842	0.805137	0.521739	0.207237	0.077307	19	1	Terpene
BGC_030_neof	BGC_475_humi	0.244482	0.570807	0.894737	0.715427	0.8	0.272344	0.394636	18	1	Terpene
BGC_216_mala	BGC_282_spum	0.189048	0.657643	0.9	0.799603	0.625	0.200515	0.199219	20	1	Terpene
BGC_184_sihw	BGC_478_hank	0.296898	0.494353	1	0.615247	0.833333	0.380244	0.423077	17	1	Terpene
BGC_184_sihw	BGC_475_humi	0.189959	0.656166	1	0.746721	1	0.241095	0.362934	18	1	Terpene
BGC_410_west	BGC_508_hank	0.271059	0.531354	0.785714	0.716564	0.6875	0.283436	0	16	0	Terpene
BGC_443_lacu	BGC_485_terr	0.035542	0.930178	1	0.95261	1	0.045491	0.067332	21	1	Terpene
BGC_410_west	BGC_509_para	0.292851	0.50006	0.684211	0.724046	0.545455	0.289553	0.146766	19	1	Terpene
BGC_410_west	BGC_454_oryz	0.289765	0.504434	0.777778	0.698303	0.619048	0.305054	0.269802	19	1	Terpene
BGC_050_amic	BGC_387_jing	0.2568	0.552346	0.75	0.748076	0.642857	0.251924	0	15	0	Terpene
BGC_387_jing	BGC_405_rubr	0.092887	0.822853	0.888889	0.919113	0.8	0.077683	0.109726	18	1	Terpene
BGC_387_jing	BGC_509_para	0.164656	0.697799	0.823529	0.850851	0.65	0.1488	0.15212	17	1	Terpene
BGC_171_hirs	BGC_321_iter	0.165832	0.695836	1	0.778891	1	0.206194	0.280769	8	1	Terpene
BGC_050_amic	BGC_424_jaco	0.257581	0.551187	0.833333	0.720051	0.714286	0.279949	0	15	0	Terpene
BGC_405_rubr	BGC_509_para	0.218076	0.611405	0.833333	0.775899	0.666667	0.231798	0.147132	20	1	Terpene

BGC_1	BGC_2	Raw distance	Squared similarity	Jaccard index	DSS index	Adjacency index	raw DSS non-anchor	raw DSS anchor	Non-anchor domains	Anchor domains	Network
BGC_405_rubr	BGC_508_hank	0.210636	0.623096	0.846154	0.777956	0.733333	0.222044	0	15	0	Terpene
BGC_090_effu	BGC_253_hydr	0.282859	0.514291	0.846154	0.703096	0.411765	0.296904	0	13	0	Terpene
BGC_157_nami	BGC_516_para	0.135603	0.747181	1	0.819195	1	0.184693	0.149701	16	1	Terpene
BGC_030_neof	BGC_184_sihw	0.171495	0.686421	0.894737	0.812744	0.8	0.176134	0.287356	18	1	Terpene
BGC_424_jaco	BGC_509_para	0.299049	0.491333	0.722222	0.698676	0.65	0.298962	0.322581	18	1	Terpene
BGC_454_oryz	BGC_508_hank	0.166862	0.694119	0.833333	0.84101	0.714286	0.15899	0	13	0	Terpene
BGC_306_alka	BGC_398_rubr	0.06521	0.873833	1	0.913054	1	0.094747	0.02454	16	1	Terpene
BGC_405_rubr	BGC_454_oryz	0.256013	0.553516	0.823529	0.729041	0.65	0.271564	0.265509	18	1	Terpene
BGC_145_nami	BGC_454_oryz	0.232315	0.58934	0.75	0.784984	0.578947	0.209032	0.259901	15	1	Terpene
BGC_312_alka	BGC_509_para	0.247388	0.566425	0.736842	0.767598	0.590909	0.241417	0.146766	19	1	Terpene
BGC_392_jing	BGC_446_lacu	0.22729	0.59708	0.928571	0.729327	0.8	0.283593	0.180233	14	1	Terpene
BGC_417_west	BGC_516_para	0.276536	0.5234	0.75	0.718785	0.6875	0.30329	0.137725	13	1	Terpene
BGC_244_hydr	BGC_348_zhao	0.278334	0.520802	0.888889	0.68074	0.666667	0.311571	0.388462	18	1	Terpene
BGC_050_amic	BGC_443_lacu	0.200158	0.639748	0.833333	0.796615	0.714286	0.203385	0	15	0	Terpene
BGC_326_insu	BGC_478_hank	0.282204	0.515232	0.823529	0.683287	0.8125	0.323	0.266409	16	1	Terpene
BGC_073_arai	BGC_266_croc	0.218932	0.610067	1	0.726609	0.722222	0.279297	0.217284	19	1	Terpene
BGC_030_neof	BGC_048_shan	0.243594	0.57215	0.875	0.725208	0.75	0.264787	0.344828	14	1	Terpene
BGC_062_amic	BGC_392_jing	0.297016	0.494187	0.533333	0.761757	0.5	0.258882	0.155689	8	1	Terpene
BGC_306_alka	BGC_516_para	0.207233	0.628479	0.882353	0.769877	0.777778	0.240176	0.149701	16	1	Terpene
BGC_312_alka	BGC_387_jing	0.271599	0.530568	0.684211	0.752381	0.545455	0.263184	0.099751	19	1	Terpene
BGC_084_effu	BGC_523_aspl	0.257355	0.551521	0.866667	0.718341	0.611111	0.287931	0.234615	15	1	Terpene
BGC_050_amic	BGC_508_hank	0.248946	0.564083	0.833333	0.731565	0.714286	0.268435	0	15	0	Terpene
BGC_050_amic	BGC_509_para	0.140883	0.738082	0.916667	0.848664	0.785714	0.151336	0	15	0	Terpene
BGC_405_rubr	BGC_424_jaco	0.270921	0.531556	0.777778	0.728334	0.545455	0.264814	0.333333	18	1	Terpene

BGC_1	BGC_2	Raw distance	Squared similarity	Jaccard index	DSS index	Adjacency index	raw DSS non-anchor	raw DSS anchor	Non-anchor domains	Anchor domains	Network
BGC_167_aich	BGC_432_jaco	0.234386	0.586165	0.888889	0.73115	0.789474	0.279114	0.176471	18	1	Terpene
BGC_062_amic	BGC_446_lacu	0.281106	0.516809	0.823529	0.710346	0.428571	0.302693	0.19186	15	1	Terpene
BGC_050_amic	BGC_312_alka	0.048515	0.905324	1	0.935313	1	0.064687	0	15	0	Terpene
BGC_178_hirs	BGC_320_iter	0.283942	0.512739	0.5625	0.776674	0.421053	0.223326	0	13	0	Terpene
BGC_398_rubr	BGC_516_para	0.212607	0.619987	0.882353	0.762711	0.777778	0.248238	0.149701	16	1	Terpene
BGC_090_effu	BGC_297_phth	0.29741	0.493633	0.785714	0.701337	0.388889	0.298663	0	13	0	Terpene
BGC_410_west	BGC_443_lacu	0.279451	0.519192	0.52381	0.789166	0.478261	0.210834	0	16	0	Terpene
BGC_062_amic	BGC_398_rubr	0.086475	0.834528	1	0.8847	1	0.116108	0.108434	17	1	Terpene
BGC_446_lacu	BGC_494_terr	0.111353	0.789694	0.941176	0.878981	0.823529	0.098381	0.302128	16	1	Terpene
BGC_237_desu	BGC_454_oryz	0.286218	0.509485	0.8	0.689891	0.727273	0.310109	0	11	0	Terpene
BGC_184_sihw	BGC_299_phth	0.222523	0.60447	0.842105	0.768741	0.65	0.227348	0.262548	16	1	Terpene
BGC_184_sihw	BGC_348_zhao	0.261817	0.544914	0.944444	0.684773	0.714286	0.311546	0.350195	19	1	Terpene
BGC_405_rubr	BGC_410_west	0.143598	0.733425	0.833333	0.875203	0.666667	0.129797	0.079801	18	1	Terpene
BGC_299_phth	BGC_475_humi	0.294626	0.497552	0.842105	0.672603	0.65	0.322761	0.366795	17	1	Terpene
BGC_306_alka	BGC_446_lacu	0.282836	0.514323	0.823529	0.708039	0.428571	0.311166	0.147929	15	1	Terpene
BGC_090_effu	BGC_183_sihw	0.261788	0.544957	0.846154	0.731191	0.411765	0.268809	0	13	0	Terpene
BGC_030_neof	BGC_244_hydr	0.240762	0.576442	0.842105	0.743311	0.666667	0.250727	0.310345	18	1	Terpene
BGC_306_alka	BGC_417_west	0.206945	0.628937	0.777778	0.801852	0.722222	0.216015	0.055215	16	1	Terpene
BGC_145_nami	BGC_405_rubr	0.078709	0.848778	0.833333	0.961722	0.666667	0.03398	0.074813	17	1	Terpene
BGC_184_sihw	BGC_244_hydr	0.249583	0.563126	0.842105	0.73155	0.666667	0.263235	0.315385	18	1	Terpene
BGC_387_jing	BGC_508_hank	0.178848	0.674291	0.833333	0.825028	0.714286	0.174972	0	13	0	Terpene
BGC_145_nami	BGC_387_jing	0.181451	0.670022	0.777778	0.84631	0.565217	0.157793	0.114713	19	1	Terpene
BGC_106_otit	BGC_167_aich	0.29144	0.502058	0.857143	0.667287	0.733333	0.354009	0.172996	15	1	Terpene
BGC_030_neof	BGC_216_mala	0.279063	0.51975	0.736842	0.731425	0.5	0.259941	0.333333	15	1	Terpene

BGC_1	BGC_2	Raw distance	Squared similarity	Jaccard index	DSS index	Adjacency index	raw DSS non-anchor	raw DSS anchor	Non-anchor domains	Anchor domains	Network
BGC_508_hank	BGC_509_para	0.23684	0.582413	0.833333	0.738914	0.846154	0.261086	0	15	0	Terpene
BGC_145_nami	BGC_202_soli	0.21131	0.622032	0.7	0.82365	0.619048	0.169273	0.229426	15	1	Terpene
BGC_184_sihw	BGC_216_mala	0.277784	0.521596	0.736842	0.73313	0.5	0.256635	0.343629	15	1	Terpene
BGC_282_spum	BGC_348_zhao	0.289395	0.50496	0.842105	0.672119	0.761905	0.327728	0.329412	20	1	Terpene
BGC_178_hirs	BGC_509_para	0.269785	0.533213	0.705882	0.748717	0.55	0.251283	0	16	0	Terpene
BGC_410_west	BGC_485_terr	0.274645	0.526141	0.52381	0.795574	0.478261	0.204426	0	16	0	Terpene
BGC_062_amic	BGC_306_alka	0.093348	0.822018	1	0.875536	1	0.125715	0.114458	16	1	Terpene
BGC_109_sput	BGC_424_jaco	0.253737	0.556909	0.761905	0.751843	0.6	0.262409	0.07	25	1	Terpene
BGC_244_hydr	BGC_321_iter	0.242673	0.573544	1	0.676436	1	0.315929	0.388462	17	1	Terpene
BGC_398_rubr	BGC_446_lacu	0.275681	0.524637	0.823529	0.717579	0.428571	0.300353	0.147929	15	1	Terpene
BGC_050_amic	BGC_405_rubr	0.117049	0.779603	0.916667	0.871285	0.923077	0.128715	0	15	0	Terpene
BGC_348_zhao	BGC_475_humi	0.20576	0.630817	0.944444	0.759516	0.714286	0.234653	0.292969	18	1	Terpene
BGC_387_jing	BGC_410_west	0.192367	0.652271	0.736842	0.835908	0.666667	0.169578	0.114713	18	1	Terpene
BGC_244_hydr	BGC_299_phth	0.196378	0.645809	0.789474	0.815356	0.684211	0.176955	0.242308	15	1	Terpene
